# Oedematic-atrophic astrocytes in hepatic encephalopathy

**DOI:** 10.1186/s40478-025-02045-5

**Published:** 2025-05-31

**Authors:** Mariusz Popek, Marta Obara-Michlewska, Łukasz Mateusz Szewczyk, Marcin Kołodziej, Karol Perlejewski, Alexei Verkhratsky, Jan Albrecht, Magdalena Zielińska

**Affiliations:** 1 Department of Neurotoxicology, Mossakowski Medical Research Institute, Polish Academy of Sciences, Pawińskiego St. 5, Warsaw, 02-106 Poland; 2 Laboratory of Molecular Neurobiology, Centre of New Technologies, The University of Warsaw, Banacha St. 2c, Warsaw, 02-097 Poland; 3 Institute of Theory of Electrical Engineering, Measurement and Information Systems, Warsaw University of Technology, Koszykowa St. 75, Warsaw, 00-662 Poland; 4 Department of Immunopathology of Infectious and Parasitic Diseases, Medical University of Warsaw, Pawinskiego St. 3C, Warsaw, 02-106 Poland; 5 Faculty of Biology, Medicine and Health, The University of Manchester, Manchester, UK; 6 Department of Neurosciences, University of the Basque Country UPV/EHU, IKERBASQUE, Basque Foundation for Science, Leioa, Bilbao, 48940 Spain; 7 Research Centre on TCM-Rehabilitation and Neural Circuit, School of Acupuncture and Tuina/Health and Rehabilitation, Chengdu University of Traditional Chinese Medicine, Chengdu, China; 8 Department of Forensic Analytical Toxicology, School of Forensic Medicine, China Medical University, Shenyang, China; 9 Department of Stem Cell Biology, State Research Institute Centre for Innovative Medicine, Vilnius, LT-01102 Lithuania

**Keywords:** Astrocyte, Acute liver failure, Aquaporin 4, Ezrin, Profilin-1

## Abstract

Hepatic encephalopathy (HE) following acute liver failure (ALF) is considered as a primary toxic astrocytopathy, but in-depth characterisation of astrocytic contribution to the pathogenesis of this disease is far from complete. Using transmission electron microscopy, confocal fluorescent microscopy, and 3D reconstruction, we found complex morphological alterations of cortical astrocytes in mice with azoxymethane-induced ALF and post-mortem cortical tissue of patients at grade IV of HE. In both mice and post-mortem human tissues astrocytic primary branches demonstrated the territory occupied by astrocytes was increased, confirming astrocytic oedema. Astrocytic primary branches demonstrated swelling, while terminal leaflets showed atrophy quantified by the reduced area occupied by astrocytes, decreased number and the length of leaflets, decreased leaflets volume fraction, and altered astrocyte-to-neurone landscape. In mice these morphological changes develop in parallel with decreased expression of proteins critical for astrocytic modelling and function: the water channel aquaporin 4 (AQP4), the phosphorylated leaflet-associated ezrin, and the actin dynamics regulator, profilin 1 (PFN1). Pathological changes in astrocytes develop in parallel, and are likely causally linked to, the HE-linked neurological decline, manifested by a reduction in EEG power and by excessive glutamate in the brain microdialysates. We propose that HE evokes disease-specific remodelling of astrocytes to a “mixed”, oedematic/atrophic phenotype. Concurrence of HE-specific phenotype with alterations in the expression of astrocytic proteins are a likely cause of aberrant astrocyte synaptic support resulting in severe, often fatal brain malfunction in HE.

**Supplementary Information:**

The online version contains supplementary material available at 10.1186/s40478-025-02045-5.

## Introduction

Hepatic encephalopathy (HE), the primary astrocytopathy [1, 2], is a complex, multi-symptomatic neurological syndrome caused by a systemic increase in ammonium [3] usually linked to an acute or chronic liver failure (ALF and CLF respectively). Ammonium circulating in excess enters the brain, where it is detoxified and converted to glutamine by astrocyte resident glutamine synthetase [4, 5]. Pathological increase of glutamine followed with dysregulation of other osmolytes leads to a multifaceted homeostatic imbalance [6], clinically manifested with delirium, hallucinations, acute psychosis, and, in a terminal phase, coma leading to death [7]. In HE astrocytes undergo multiple morphological changes [7, 8] ranging from reactive astrogliosis, swelling, prominent in the cortical gray matter that contributes to brain oedema, and the emergence of disease-specific aberrant astrocytes known as Alzheimer type II astrocytes, the term introduced in 1942 [9].

Acute liver failure (ALF) is employed as an experimental model of fulminant HE in humans in which survival rates without liver transplant range from 10 to 40% [10]. Animals subjected to ALF demonstrate prominent neurological decline and synaptic impairments [11, 12], associated with brain oedema as well as the abnormal flow of the cerebrospinal fluid [13]. Experimental observations revealed that ionic composition and acid-base balance of the interstitial fluid remain well controlled [13], a feature possibly contributing to the partial brain function recovery observed in human patients [14, 15].

Protoplasmic astrocytes are characterised by a complex morphology defined by an extensive arborisation comprised of primary processes known as branches and distal exceedingly thin processes defined as leaflets [16, 17]. Leaflets, often associated with synapses, demonstrate remarkable morphological plasticity relevant to synaptic function [18]. Morphological plasticity of astrocytic processes is regulated by the leaflet-resident ezrin, a linker protein, connecting plasmalemma with the actin cytoskeleton [19, 20]. Astrocytic arborisation may also be regulated by profilins linked to actin dynamics. In particular, profilin 1 (PFN1) was shown to regulate astrocyte stellation [21] and Ca^2+^-induced extension of leaflets in cultured astrocytes [22]. Another astrocyte-specific protein associated with the regulation of astrocyte volume and morphology is the water channel aquaporin 4 (AQP4) [23], which, in the healthy brain, is primarily localised to astrocytic endfeet plastering blood vessels [24]. Two classical isoforms, AQP4a (M1) and AQP4c (M23) are alternatively spliced transcripts of the *AQP4* gene that participate in the cell volume regulation of astrocytes [25]. The M23 isoform is also involved in the remodelling of the astrocytic processes, especially of those in the vicinity of the glutamatergic synapses [26].

An in-depth analysis of astrocytic branches and leaflets and synaptic landscape in the HE, however, has not been performed. Here we present data indicating complex changes in morphology of protoplasmic astrocytes in the HE induced by ALF in mice and extend them to human post-mortem brain tissue. Microscopic studies of preparations from mice and humans presented similar unique changes in astrocyte morphology. While astrocytic principal branches and soma increase their volume, terminal leaflets show prominent atrophy leading to decreased synaptic astrocytic coverage and altered astrocyte-synaptic landscape. These morphological aberrations were paralleled by substantial changes in the astrocytic content of phosphorylated ezrin, PFN1, and AQP4. Morphology and protein changes correlate with overall inhibition of the brain activity documented by EEG, reflecting loss of astrocytic homeostatic function and pathological astrocyte-neuron communications that may be directly associated with HE neuropathology.

## Materials and methods

### Animal models

Experiments were performed on 10–12 weeks male C57Bl mice from the colony of the Mossakowski Medical Research Institute, Polish Academy of Sciences, and on Aldh1l1_Cre_ERT2:TdTomatoWT/fl mouse line Control TdT+], form the colony of Centre of New Technology. All experimental procedures conformed EC Directive 86/609/EEC, with approval and under surveillance of the IV^th^ Local Ethical Committee in Warsaw, Poland.

### Azoxymethane model of ALF in mice

The induction of ALF was performed as in [12] (details in Additional file 1).

### Human cortical tissue

The specimens of cortical tissue were obtained from 8 donors (5 Controls and 3 HE patients) of both sexes in the range of ages from 25 to 63 years. Basic characteristics are presented in Table 1, included in the Additional materials. Samples were obtained from Department of Transplantation and Liver Surgery UCC MUW and Department of Immunopathology of Infectious and Parasitic Diseases MUW (Warsaw, Poland). Brain cortex samples were stored in a deep-freeze room at constant − 80 °C avoiding freeze-thaw cycles, and then underwent further procedures.

### Transmission electron microscopy of human and mouse brain tissue

Details on transmission electron microscopy are found in Additional file 1.

### Brain Preparation and immunohistochemistry of human and mouse brain tissue

The preparation of brain samples, immunostaining and confocal microscope imaging are described in Additional file 1.

### Hematoxylin and eozin mouse liver staining

The preparation of mouse liver samples, hematoxylin and eozin staining and light microscopy imaging are described in Additional file 1.

### Serum biochemical analysis

Concentration of mice serum glucose, ammonium, alanine aminotransferase (ALT), total protein, cholesterol, and alkaline phosphatase (ALP) were analysed using IDEXX Catalyst One (IDEXX Laboratories, Inc., Westbrook, Maine, U.S.A.) and described in Additional file 1.

### Real-time PCR

The RNA extraction [27], reversed transcription and real-time PCR for the relative mRNA quantification [28] of AQP4 and its isoforms, M23 and M21 [29], are described in Additional file 1.

### Western blot

The AQP4, PFN1 and ezrin protein expression was assessed by means of Western blot and described in Additional file 1.

### Astrocytic processes distribution and sholl analysis

The details of astrocytic arborisation assessment methods are provided in Additional file 1.

### Astrocytes and neurons volume quantification

The measurement of volume of astrocyte– neuron contact space is described in Additional file 1.

### EEG recording of the freely moving mice

The EEG recording was performed as described in [30] (details in Additional file 1).

### Microdialysis of the freely moving mice

The microdialysis from the cerebral cortex was performed as described in [11] (details in Additional file 1).

### High-performance liquid chromatography

The glutamate concentration in the brain microdialysates by HPLC was performed as described in [31] (details in Additional file 1).

### Statistical analysis

Details are found in Additional file 1.

### Data Availability

Data are available upon reasonable request.

## Results

### Biochemical and morphological characteristics of ALF mice

**Fig. 1:**
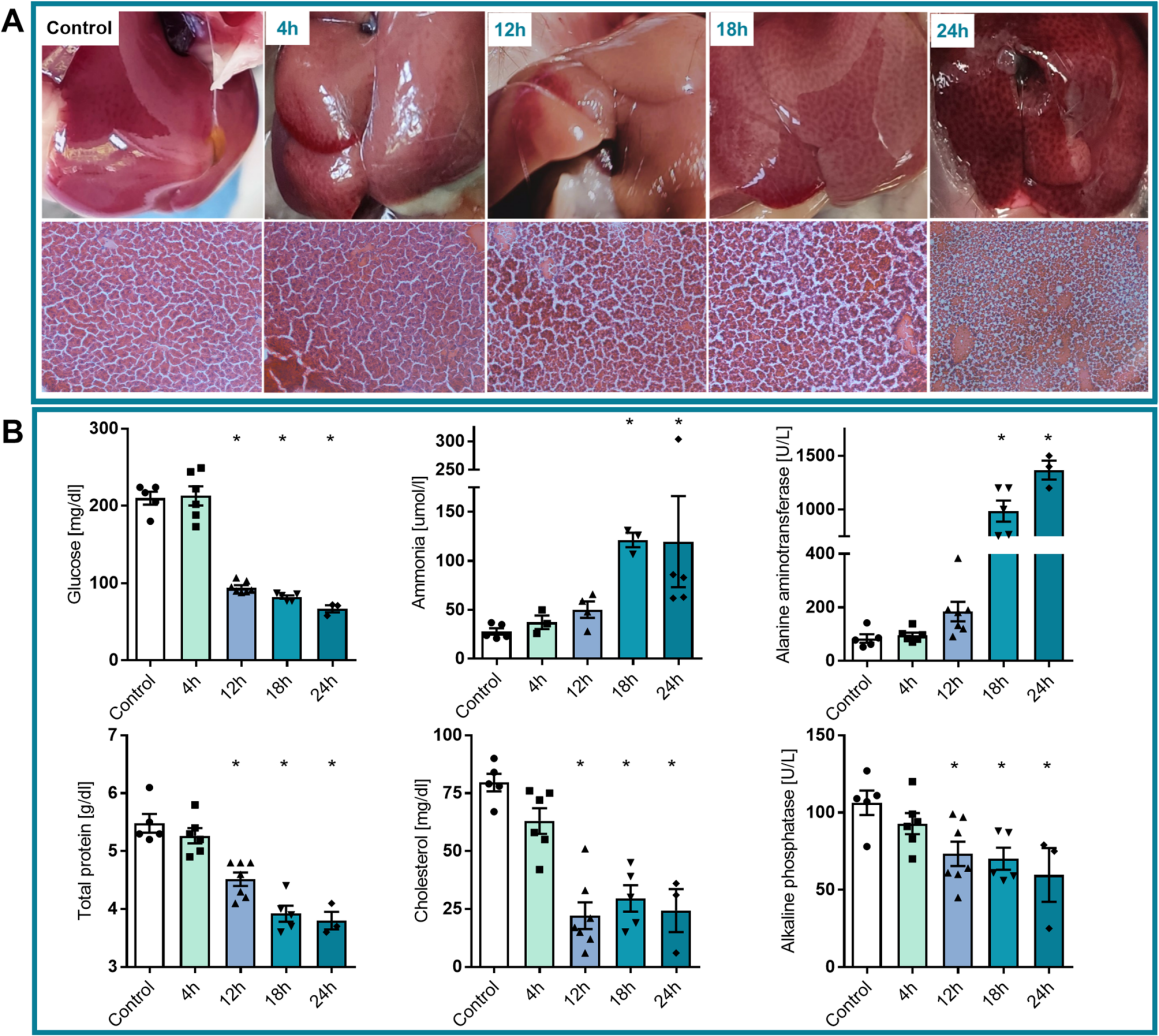
Validation of ALF mice model. **A**: Morphological and histopathological assessment of liver failure progression at 4, 12, 18, and 24 h after azoxymethane (AOM) injection **B**: Serum levels of glucose, ammonium, ALT, total protein, cholesterol, and ALP at 4, 12, 18, and 24 h after AOM injection. *n* = 4–7, *p < 0.05 ANOVA with Dunnett’s post hoc test

Progression of the ALF in mice was monitored at 4, 12, 18, and 24 h after AOM injection. Gradual deterioration of biochemical parameters was measured in blood serum (Fig. 1B), confirming the evolution of liver failure and HE symptoms. Histological examination of mice livers exposed irregular and speckled surface observed as early as 4 h, which gradually progressed through the later time points. Fine, bluish-red pattern of bleeding was observed at 12 h post-AOM; necrotic changes progress at 18 h post-AOM, and extensive necrotic spots were obvious at 24 h (Fig. 1A). Histopathological assessment with hematoxylin-eozin staining revealed tissue degeneration from 4 h, hepatocyte apoptosis, and partial hyperaemia at 12 h, followed by necrosis of hepatocytes, the fibre mesh scaffold collapse and inflammatory invasion at 18 h with damage further increasing at 24 h (Fig. 1A).

Hyperammonemia (from 28 ± 7.4 µM in control, *n* = 5, to 120 ± 12.7 µM at 18 h and 24 h, *n* = 3 and 5, respectively) and increased alanine aminotransferase (ALT) level (from 84 ± 35 U/L in control, *n* = 5, to 984 ± 220 U/L at 18 h, *n* = 5, and 1366 ± 153 U/L at 24 h, *n* = 3) were evident at 18 h after AOM injection (Fig. 1B). The serum glucose level decreased at 12 h (from 210 ± 18 mg/dl, *n* = 5 in control, to 94.7 ± 7.5 mg/dl, *n* = 7 in 12 h HE) after AOM administration (Fig. 1B). Similarly, we observed decreased total protein (from 5.5 ± 0.4, *n* = 5, to 4.5 ± 0.3 g/dl at 12 h, *n* = 7, and to 3.8 ± 0.3 g/dl at 24 h, *n* = 3), cholesterol (from 80 ± 8 mg/dl, *n* = 5, to 22 ± 15 mg/dl at 12 h, *n* = 7, and to 24 ± 16 mg/dl at 24 h, *n* = 3), and ALP (from 106 ± 18 U/L, *n* = 5, to 73 ± 21 U/L at 12 h, *n* = 7, and to 60 ± 30 U/L at 24 h, *n* = 3) (Fig. 1B).

### Electron microscope imaging of the HE mice brain cortex

**Fig. 2:**
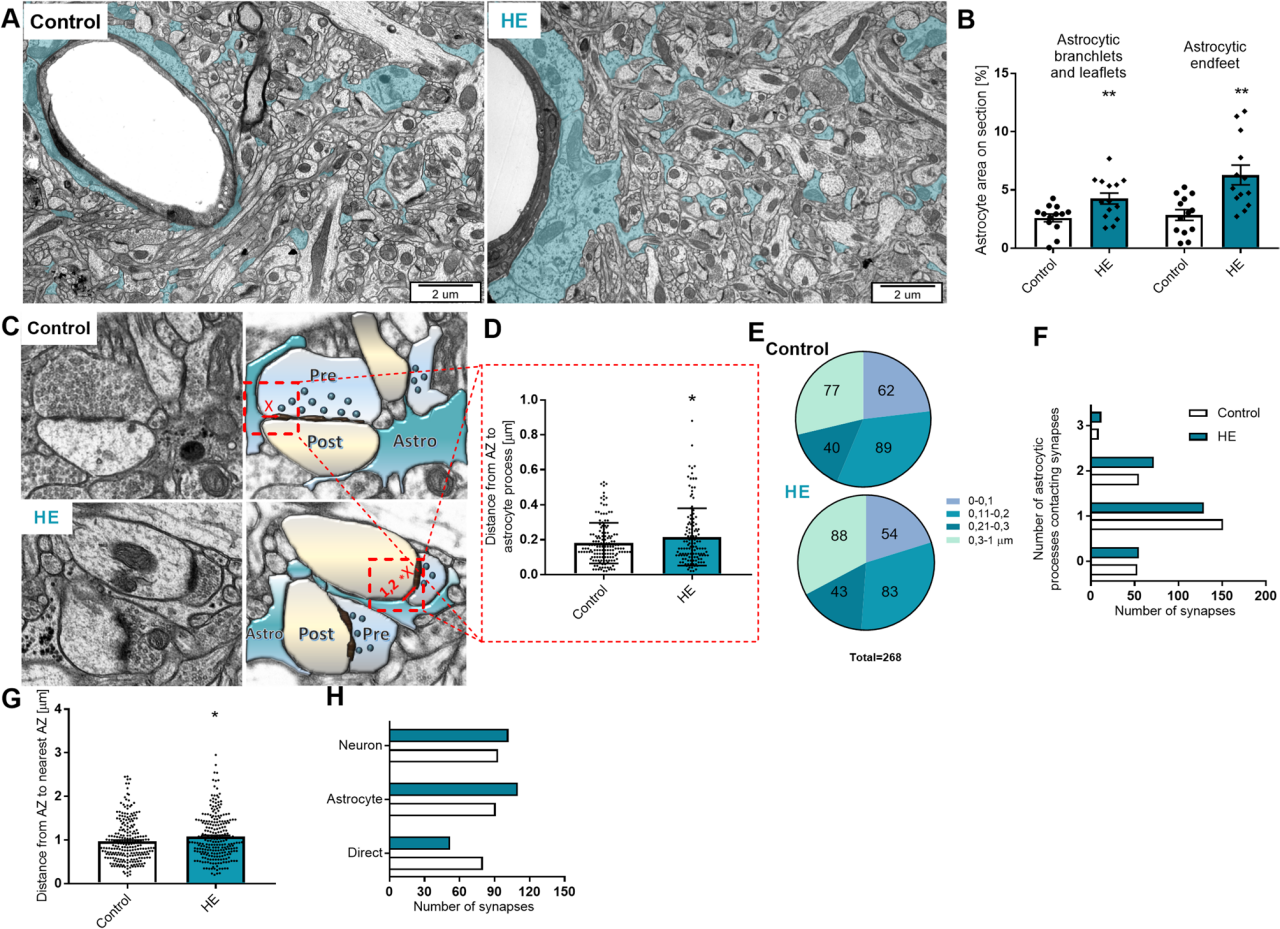
Ultrastructural pathohistology of HE astrocytes. **A**: Astrocytic profiles (navy blue) in a single thin section from control and HE (AOM 24 h) mice **B**: Area of astrocytic processes referred to the whole analysed section from control and HE mice, *n* = 3 mice for control and 4 mice for HE, 13 sections for control and 14 sections for HE, * *p* < 0.05, t-Test **C**: representative morphological interactions between astrocytic processes (Astro, navy blue) and synapses (presynaptic terminal– Pre, blue and postsynaptic bouton– Post, yellow) in control and HE mice; x denotes the distance between the active zone (AZ) edge and the nearest astrocytic membrane **D**: Distance from AZ edge to the nearest astrocytic process, analysed in synapses which contact single astrocytic process (149 for control; and 129 for HE), *n* = 3–4 mice, *p < 0.05, t-test **E**: Number of synapses in which the distance between AZ edge and astrocyte process was in the range between 0-100, 101–200, 201–300, and 300–1000 nm **F**: Distribution of the number of astrocytic processes contacting synapses; 268 synapses from control and 268 synapses from HE mice **G**: Distance from AZ edge to the nearest synapse AZ edge, 268 synapses were analysed from both groups, *n* = 3–4 mice, *p < 0.05, t-test **H**: Comparison between analysed synapses; (Directly– the nearest synapses are in direct contact, Astro– between the nearest synapses astrocytic processes is present, Neuro– neuronal component present between the nearest synapses), 268 synapses from *n* = 3–4 mice were analysed

To test whether ALF-induced HE modified astrocyte-synaptic landscape, we quantified cell ultrastructure. Astrocytic processes were identified by their irregular shape, the presence of glycogen granules and bundles of intermediate filaments in a comparatively clear cytoplasm (Fig. 2A). The astrocyte area in the section was determined by outlining and computing the area of all astrocytic processes and dividing by the total area of each section. Astrocytic endfeet in direct contact with blood vessels were counted separately from parenchymal processes classified as branches and leaflets. Sections with astrocytic cell bodies were not included in the analysis (1 section for the control group and 2 for HE). The area of astrocyte in relation to the area of section was counted; each cell body (either astrocyte with a visible nucleus or a neurone) occupying more than 20–25% of entire section introduces deviation, therefore the analysis of such section was subjectively excluded. In the control group, *n* = 4, astrocytic endfeet occupied 2.85 ± 1.5% and astrocytic branches and leaflets 2.6 ± 1.2% of the total area while in HE mice, *n* = 3, they occupied, respectively, 6.3 ± 3% and 4.3 ± 1.7% (Fig. 2B). HE, therefore, is associated with a significant increase in astrocyte occupying territory, irrespectively of separate quantification of astrocytic endfeet or branches with leaflets.

Excluding ’no contact synapses‘, the distance between the nearest astrocyte process to the synaptic cleft (the distance to the proximal end of the active zone (AZ)) increased by ~ 20% in HE group, from 0.18 ± 0.11 to 0.22 ± 0.16 μm (based on 151 and 129 synapses, *n* = 4 and 3 in control and AOM-injected, respectively) (Fig. 2C, D). The same analysis binned to < 0.10 μm, 0.10–0.20 μm, 0.20–0.30 μm and > 0.30 μm indicates decrease in the number of astrocytic leaflets within the 0–0.10 and 0.10–0.20 μm distance from synapse and slight increase at > 0.3 μm distance range in HE compared to controls (Fig. 2E). The number of synapses with no contact with astrocyte and synapses contacted by 1, 2, or 3 astrocytic leaflets did not differ between groups (Fig. 2F). Distance between the nearest synapses measured as AZ edge to nearest AZ edge path length was 0.96 ± 0.47 μm in the control group and 1.07 ± 0.51 μm in the HE group (Fig. 2G).

The synaptic landscape was also qualitatively compared between control and HE animals. The comparison between analysed synapses indicates that 80 synapses in control and 52 in HE were in direct contact, whereas 91 nearest synapses from control and 110 synapses from HE were contacting with astrocytic processes. In turn, between 93 nearest synapses from control and 102 synapses from HE, neuronal component was observed in synaptic vicinity (Fig. 2H).

### Changes in the expression of aquaporin 4, profilin 1, ezrin, and phospho-ezrin in HE

**Fig. 3:**
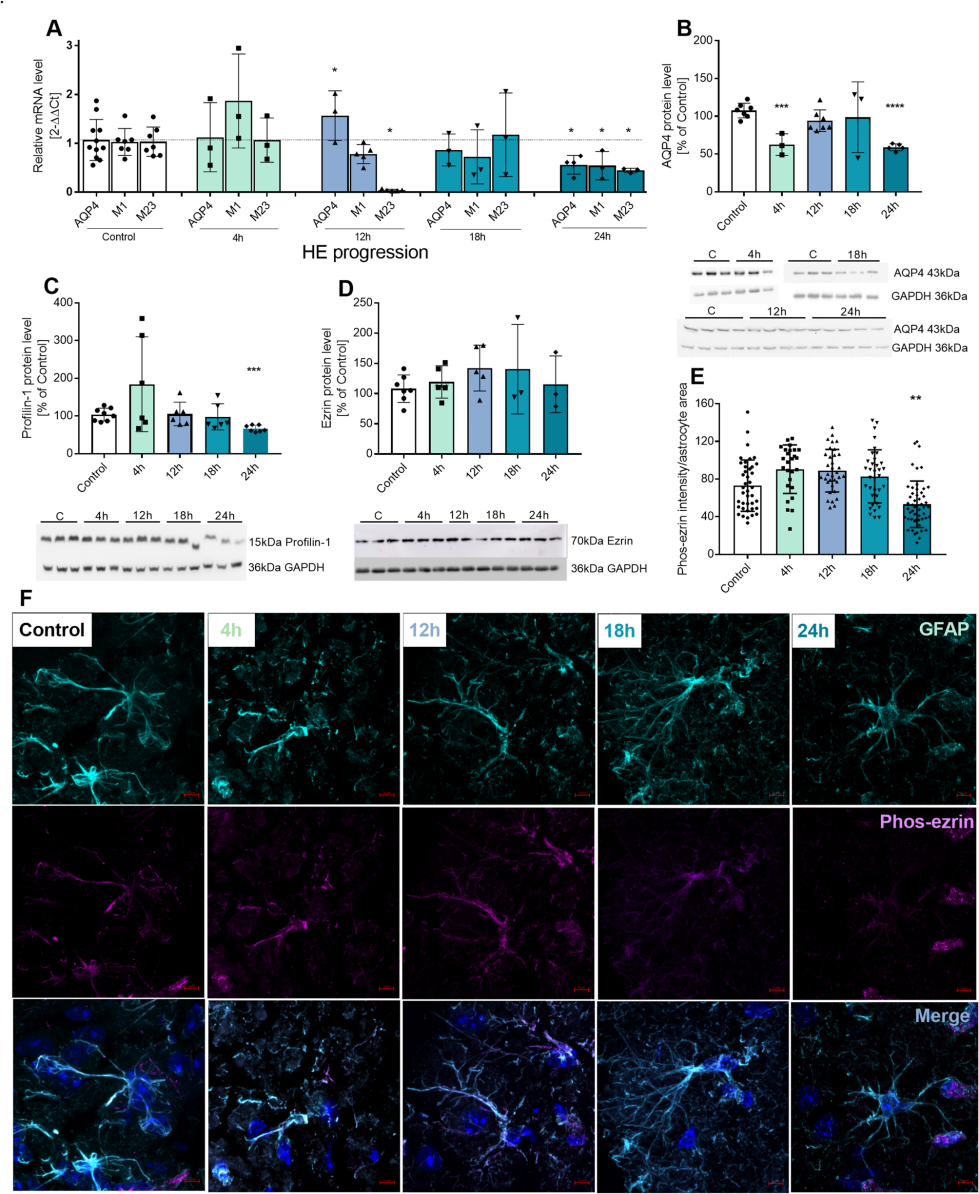
Changes of astrocytic aquaporin 4 (AQP4), profilin 1 (PFN1), ezrin, and phosphorylated-ezrin (Phos-ezrin) in the cortex of mice during HE progression. **A**: mRNA level of AQP4 and its M1 and M23 isoforms at 4, 12, 18, and 24 h after AOM injection. *n* = 4–11, * *p* < 0.05 (Mann-Whitney) **B-D**: Protein level of AQP4 (B), PFN1 (C), and ezrin (D) at 4, 12, 18, and 24 h after AOM injection *n* = 3–7, * *p* < 0.05, **p < 0.0025, *** *p* < 0.0003 (Mann-Whitney) **E-F**: Phos-ezrin (E– quantification of staining intensity, F– confocal microscopy images) in mice cerebral cortex at 4, 12, 18, and 24 h after AOM injection, *n* = 4, ** *p* < 0.01 (Mann-Whitney)

We measured the levels of AQP4 isoforms mRNA (Fig. 3A) and protein level of AQP4, ezrin, and PFN1 and analysed fluorescence intensity of phosphorylated ezrin; (*n* = 11 and 3 in control and AOM-injected, respectively; *n* = 11 and *n* = 4 in control and AOM-injected, respectively; *n* = 7 and 3 in control and AOM-injected, respectively; *n* = 7 and *n* = 5 in control and AOM-injected, respectively). Protein level of AQP4, detected with an antibody specific against the C-terminus of both M1 and M23 isoforms, decreased by 42% (from 107.6% ± 9.69 in control, to 62.55% ± 14.24) at 4 h (*n* = 7 and 3 in control and AOM-injected, respectively) and by 49% (to 58.98% ±4.52) at 24 h post-AOM injection (*n* = 7 and 5 in control and AOM-injected, respectively), the effects being separated by a return to control level at 12 h and 18 h post-AOM (Fig. 3B). The level of AQP4 M1 isoform mRNA decreased by 47% (from 1.03 ± 0.28% in control to 0.56 ± 0.19) at 24 h post-AOM (*n* = 7 and 3 in control and AOM-injected, respectively), whereas AQP4 M23 isoform mRNA dropped by 96% (from 1.035 ± 0.3 in control, to 0.04 ± 0.02) at 12 h (*n* = 7 and 5 in control and AOM-injected, respectively), rose back to the control level at 18 h (*n* = 7 and 3 in control and AOM-injected, respectively), and decreased again by 57% (to 0.44 ± 0.05) at 24 h (*n* = 7 and 3 in control and AOM-injected, respectively) (Fig. 3A). Protein level of PFN1 at 24 h post-AOM decreased by 36% (from 103.8% ± 16.95 in control to 66.75% ±8.64; *n* = 8 and 7 in control and AOM, respectively) while it was not changed at the earlier time points (Fig. 3C). The total protein level of ezrin remained unchanged throughout (Fig. 3D). However, the analysis of the active, phosphorylated ezrin, imaged in Z-stacks from GFAP-labelled astrocytes, revealed its decrease by 27.4% in 24 h post-AOM (from 73.1 ± 27.2 to 53.3 ± 24.6; 40 astrocytes for control and 53 for HE, *n* = 4), with no changes at the earlier post-AOM times (Fig. 3E-F).

### Remodelling of astrocytic arborisation in HE

**Fig. 4:**
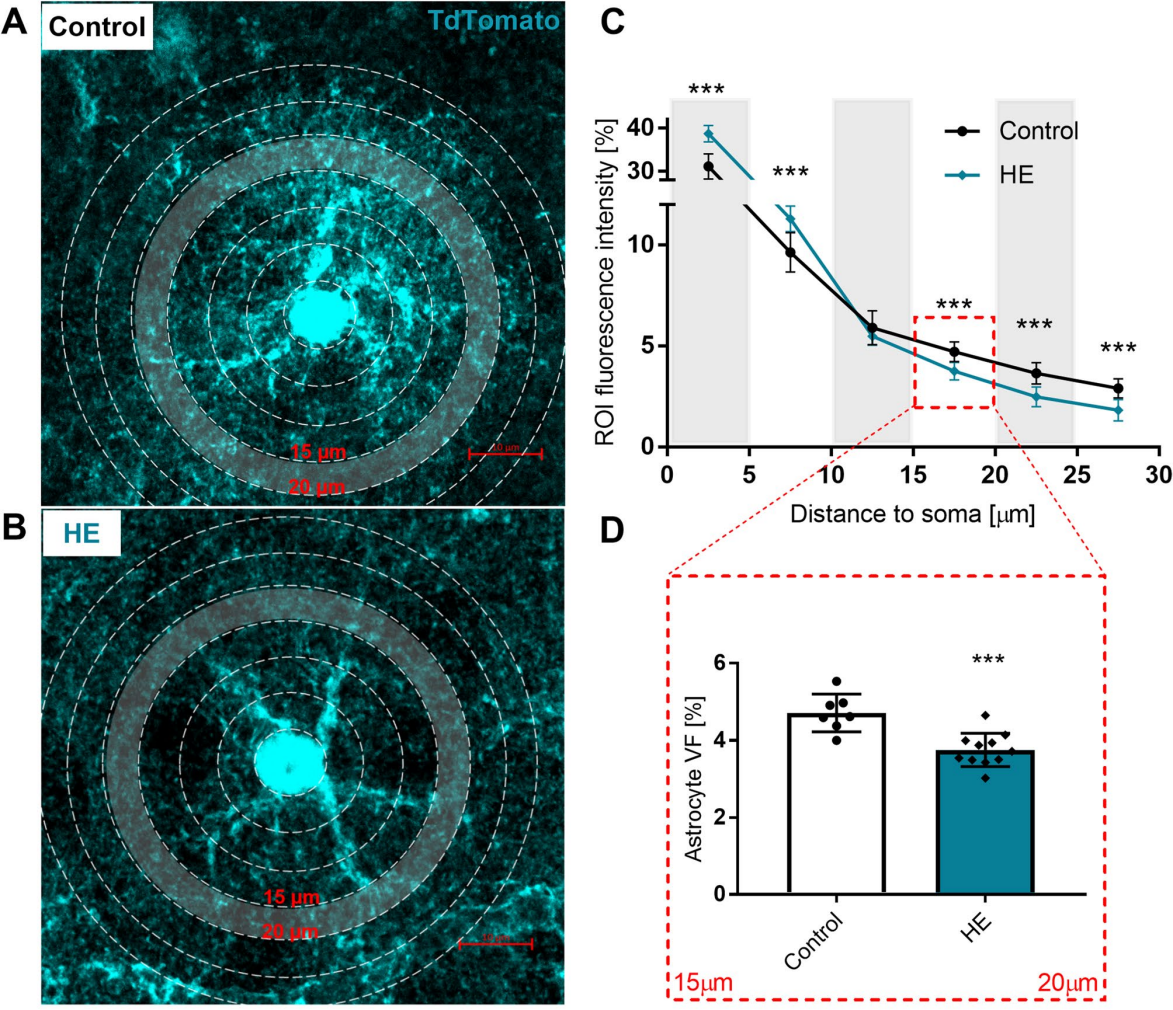
Immunofluorescent analysis of cortical astrocytic processes distribution in HE mice. **A**,** B**: Positioning of ROIs **C**: intensity of TdT fluorescence in 5 μm rings starting from the soma, **D**: Volume fraction of the area between 15–20 μm from the soma, indicating the fractional volume of the astrocyte. *n* = 4 ***p < 0.001 ANOVA with Dunnett’s post hoc test

The fluorescence intensity of TdT + astrocytic processes was measured in 5 μm ring-shaped ROIs starting from the soma. Quantification of the ROI fluorescent intensity of maximum intensity Z-projected images (7 slices, 74 astrocytes, *n* = 3 mice for control group; 11 slices, 96 astrocytes, *n* = 4 mice for HE group) revealed increased intensity in two areas nearest to cell soma (0–5 μm and 5–10 μm), by 24.4% (from 31.1 ± 2.9 to 38.7 ± 1.9) and 17.3% (from 9.6 ± 1.0 to 11.3 ± 0.6), respectively. No changes were detected in 10–15 μm area while intensity was decreased in the areas further from the soma (15–20 μm, 20–25 μm, and 25–30 μm), by 20.4% (from 4.7 ± 0.5 to 3.7 ± 0.4), 31.9% (from 3.6 ± 0.5 to 2.5 ± 0.5) and 37.5% (from 2.9 ± 0.5 to 1.8 ± 0.5), respectively (Fig. 4A, C). This pattern indicates hypertrophy of soma and primary branches and atrophy of distal astrocytic processes in HE mice.

Volume fraction (VF) of leaflets, comprising fluorescence intensity from the entire area within 15–20 μm from the soma was analysed in Z-Stack images. Mean VF was significantly reduced in the HE mice (4.71 ± 0.48%, in control; 3.75 ± 0.43% in HE; *p* < 0.001, 7 slices, 74 astrocytes, *n* = 3 mice for control group; 11 slices, 96 astrocytes, *n* = 4 mice for HE group; two-sample t-test; Fig. 4B, D), indicating atrophy of astrocytic leaflets.

**Fig. 5:**
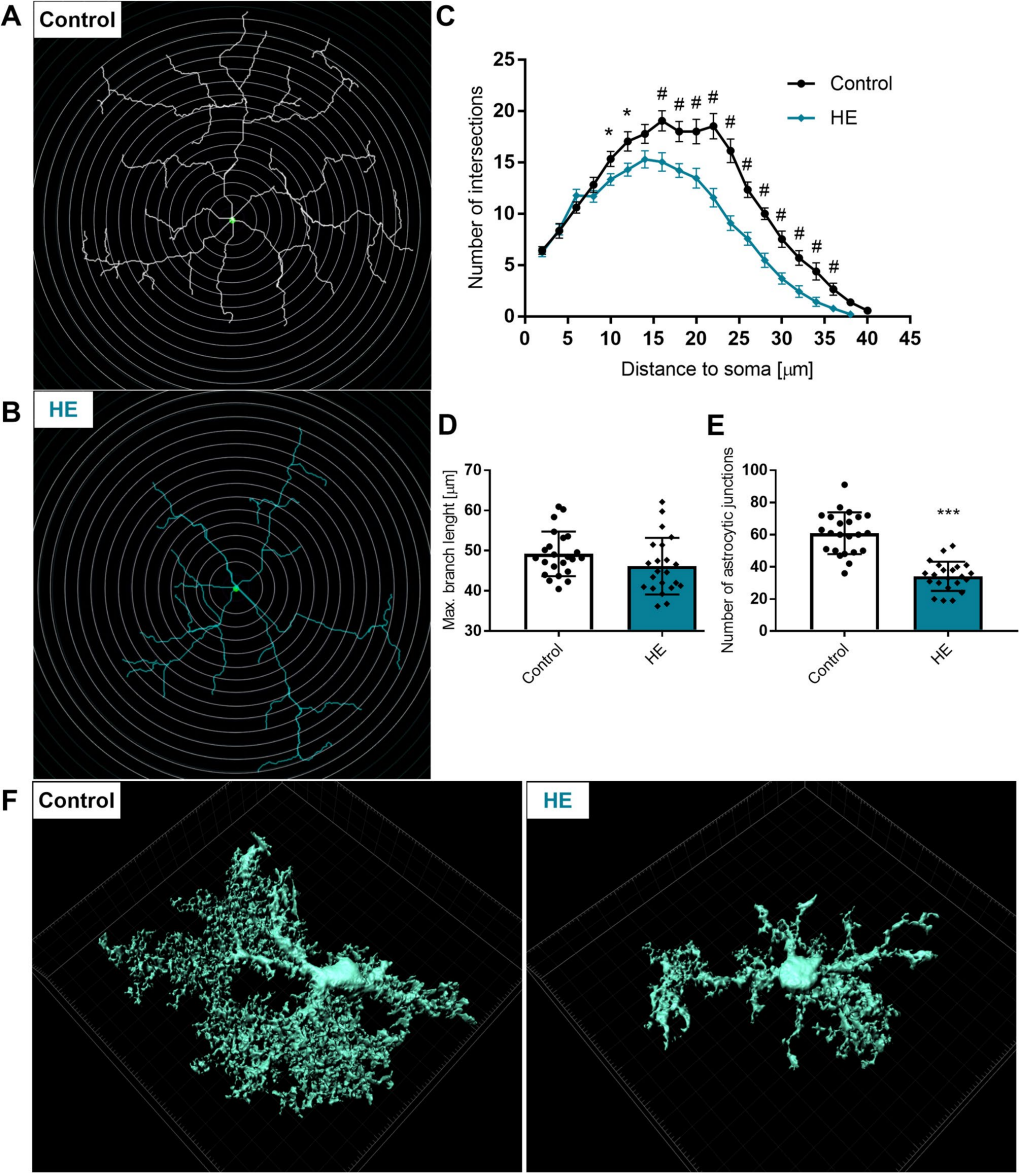
Sholl analysis of astrocytes **A-B**: Sholl plot with 2 μm interval of control and HE astrocyte **C**: Sholl plot of C and HE astrocytes, *n* = 3, * *p* < 0.05, ^**#**^*p* < 0.01, t-Test **D**: longest branch length from soma centre, **E**: number of astrocytic junctions, *n* = 3, *** *p* < 0.001, t-Test **F**: Representative 3D rendered reconstruction of control and HE astrocytes

Sholl analysis was performed on traced skeletonised TdT + astrocytes, with 2 μm intershell radii. Plot analysis (9 slices, 23 astrocytes, *n* = 3 mice from control group; 11 slices, 23 astrocytes, *n* = 3 mice from HE group) revealed decreased number of intersections between 10 μm and 36 μm from the soma in HE astrocytes; the highest number of intersections for control group was at 16 μm from the soma (19 ± 5 for control and 15 ± 4 for HE) while for HE group the highest number of intersections was detected at 14 μm from soma (18 ± 4 for control and 15 ± 4 for HE) (Fig. 5A, C). The length of the longest branch did not change (49.2 ± 5.6 for control and 46.2 ± 7.0 μm for HE) (Fig. 5B, D). Average number of intersections was significantly reduced in HE group (61 ± 13 for control and 34 ± 9 for HE) (Fig. 5E). 3D reconstructions from Z-stack also revealed substantial decrease in the complexity of arborisation in astrocytes from HE animals (Fig. 5F).

### Rearrangement of astrocyte-neuronal synaptic landscape in HE

**Fig. 6:**
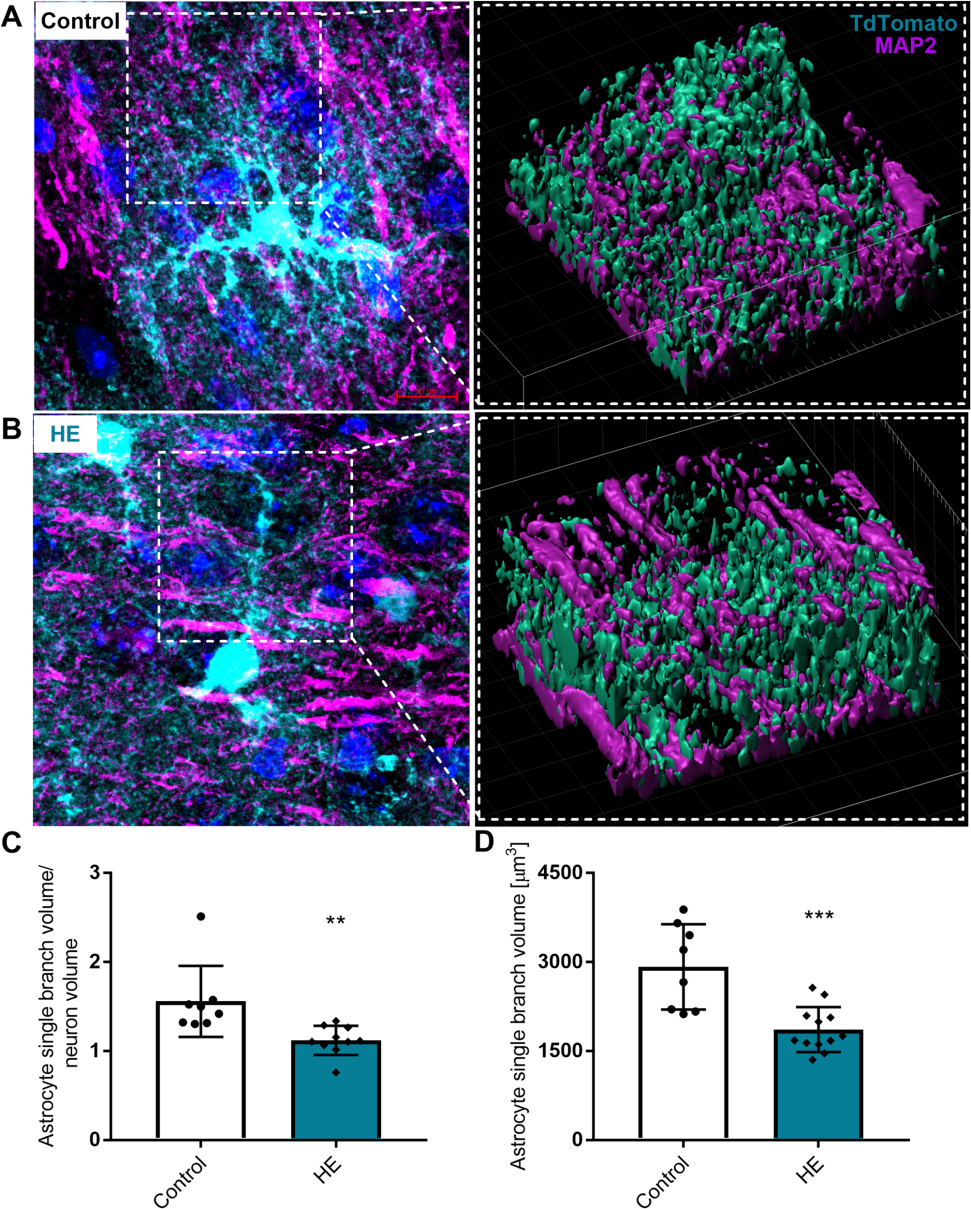
Rearrangement of astrocyte-neuronal landscape in HE. 3D reconstruction of astrocytic branch (TdT+) and neurones (MAP2+) from **A**: control and **B**: HE mice **C**: Average volume ratio of the individual astrocyte branch to neurons **D**: astrocytic individual branch volume, *n* = 4, 8 sections from the group, ** *p* < 0.01, ***p < 0.001 ANOVA with Dunnett’s post hoc test

The volume of the neurone sharing the space occupied by the astrocytic branch was quantified by analysing Z-stacks TdT + astrocyte branches and MAP2-immunostained neurons with a 3D surface rendering. Morphometric changes in the astrocyte-neuronal landscape revealed the global rearrangement of the morphological relations between astrocytic processes and neurones, with significant changes in astrocyte-neurone volume ratio of shared space. Space occupied by a single representative astrocytic arbour (including branches and leaflets) was analysed. We quantified astrocyte overlap with neighbouring neurones (MAP2-immunostaining) in control (72 analysed branches, 8 slices, *n* = 3 mice) and HE mice (99 analysed branches, 11 slices, *n* = 4 mice) (Fig. 6A, B). We observed a significant decrease in astrocyte-neurone volume ratio in the shared space by ~ 28% (1.56 ± 0.4 for control and 1.12 ± 0.16 for HE) (Fig. 6C), which was accompanied by decrease in the astrocytic volume of individual branches from 2918 ± 718 µm^3^ in control to 1861 ± 378 µm^3^ in HE mice (Fig. 6D).

### Atrophic astrocytes in HE patients

**Fig. 7:**
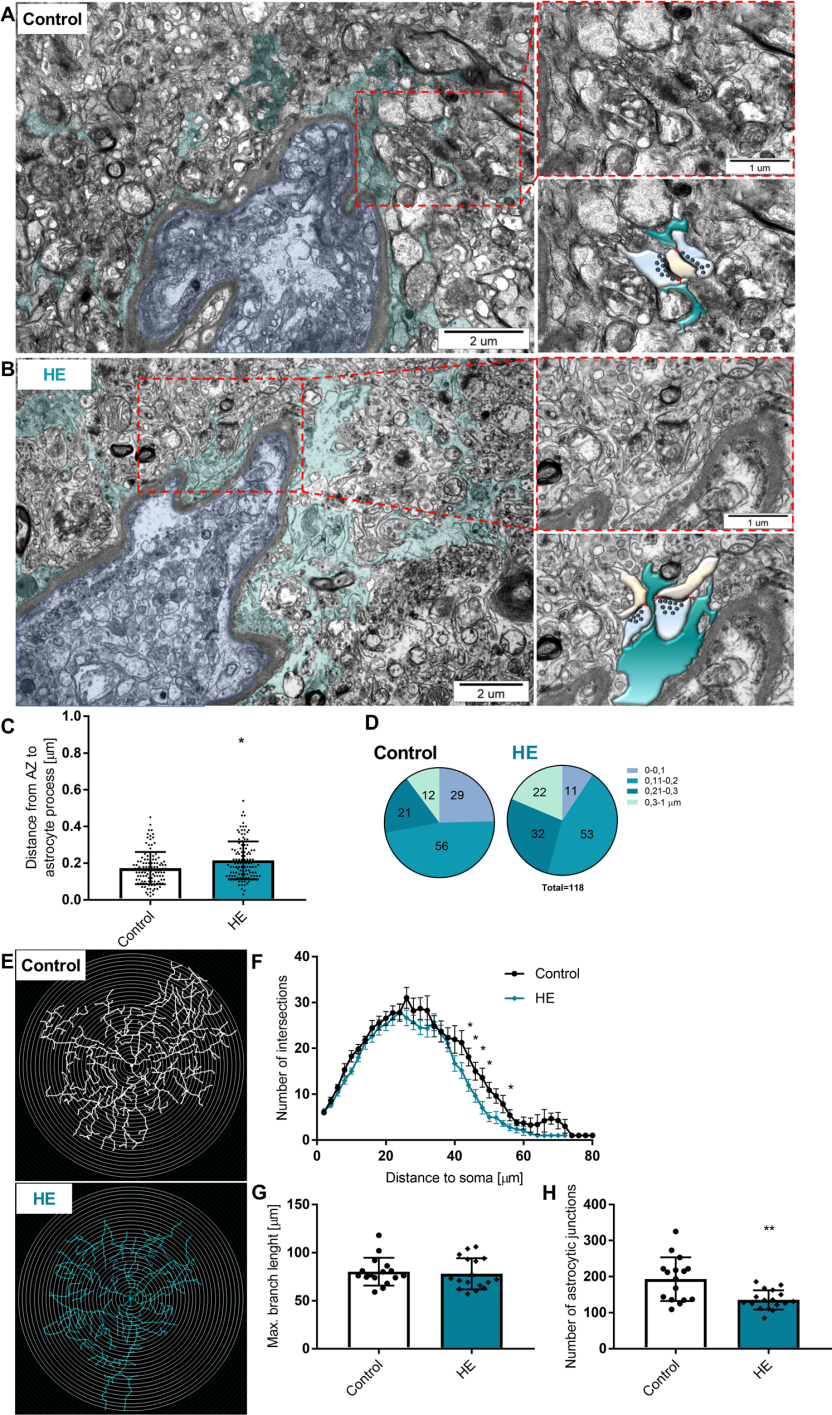
Verification of aberrant astrocytic remodelling and Scholl analysis in HE patient post mortem brain tissue. **A-B**: Ultrastructural astrocytic profiles (navy blue) in the blood vessel vicinity (blue) in a single thin section from the control and HE brain cortex of patients. Representative morphological interactions between astrocytic processes (navy blue) and synapses (presynaptic terminal–blue and postsynaptic bouton–yellow) **C**: Distance from AZ edge to the nearest astrocytic process (118 for each group), *n* = 3–4 patients, *p < 0.05, t-test **D**: Number of synapses in which the distance between AZ edge and astrocyte process was in range between 0-0.1, 0.11–0.2, 0.21–0.3, and 0.3–1 μm **E**: Sholl plot with 2 μm interval of control and HE astrocyte **F**: Sholl plot of C and HE astrocytes, *n* = 3, * *p* < 0.05, t-Test **G**: Longest branch length from soma centre point, **H**: Number of astrocytic junctions, *n* = 3, ** *p* < 0.01, t-Test

We quantified the ultrastructure of the human cerebral cortex (Table 1) to test whether HE pathology modified the astrocyte-synaptic landscape. Astrocytic processes were identified by their irregular shape, glycogen granules, and bundles of intermediate filaments in a comparatively clear cytoplasm (Fig. 7A, B). The distance between the nearest astrocyte process to the synaptic cleft (the distance to the proximal end of the active zone (AZ)) increased by ~ 24% in the HE group from 0.17 ± 0.09 to 0.22 ± 0.10 μm (based on 118 synapses from each group, *n* = 4 and 3 in control and HE patients, respectively) (Fig. 7C). The same analysis binned to < 0.10 μm, 0.10–0.20 μm, 0.20–0.30 μm, and > 0.30 μm indicates a decrease in the number of astrocytic leaflets within the 0–0.10 and 0.10–0.20 μm distance from synapse and increase at > 0.3 μm distance range in HE compared to controls (Fig. 7D).

Sholl analysis was performed on traced skeletonised GFAP-labelled astrocytes, with 2 μm inter-shell radii. Plot analysis (8 slices, 17 astrocytes, *n* = 3 patients from control group; 7 slices, 17 astrocytes, *n* = 3 patients from HE group) revealed decreased number of intersections between 44 μm and 56 μm from the soma central point in HE astrocytes; In addition, from 64 μm in the HE group, astrocytic processes disappear (only single ones were noted), whereas in the control group the number of measurable astrocytic processes is present up to 72 μm from the centre of the soma. The highest number of intersections for control group was at 26 μm from the soma centre (31 ± 9 for control and 27 ± 7 for HE) while for HE group, the highest number of intersections was detected at 24 μm from soma (28 ± 7 for control and 28 ± 7 for HE) (Fig. 7F). The length of the longest branch did not change (79.3 ± 14.4 for control and 77.9 ± 16.1 μm for HE) (Fig. 7G). An average number of intersections was significantly reduced in HE group (193 ± 60 for control and 136 ± 27 for HE) (Fig. 7H).

### Neuronal decline in acute HE in mice

**Fig. 8:**
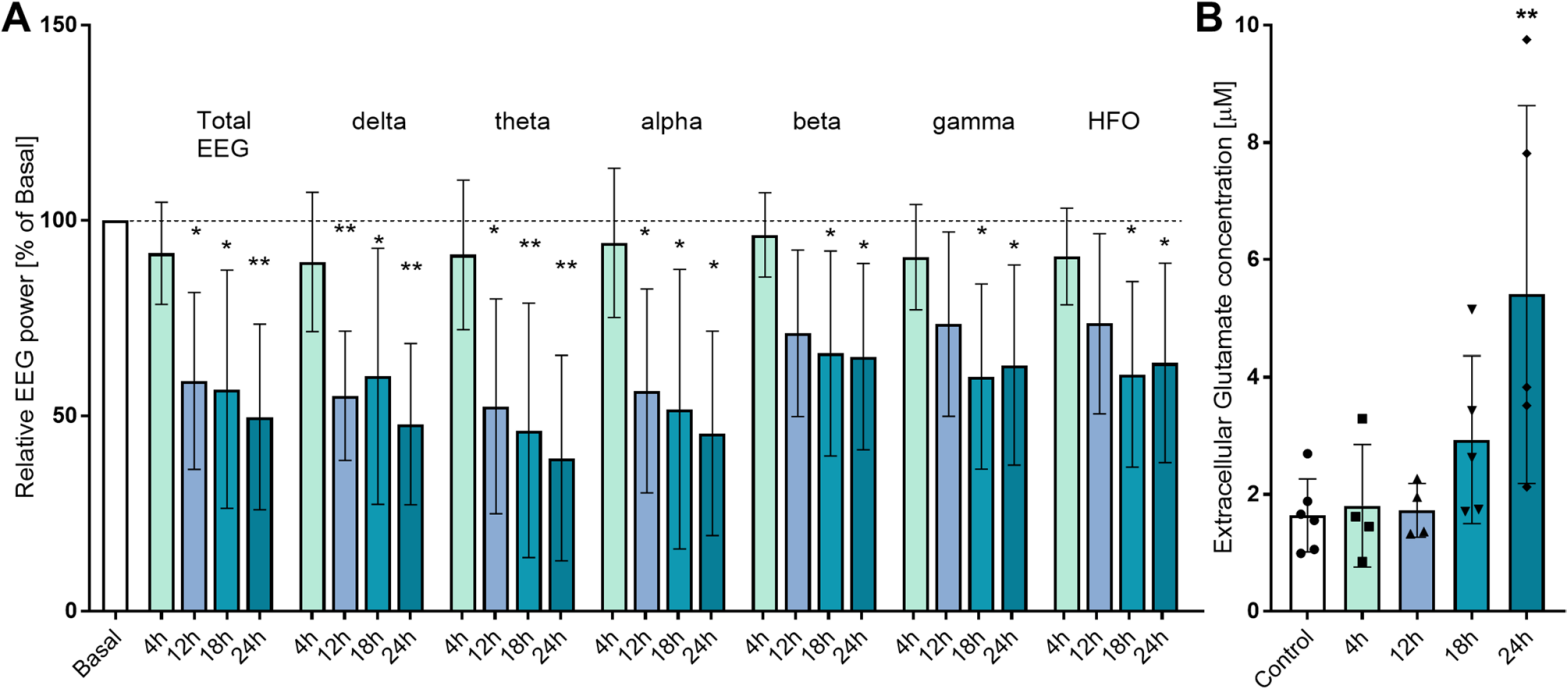
Progressive decline in neural functions in ALF mice. **A**: The relative EEG power in mice cortex at 4, 12, 18, and 24 h after AOM injection **B**: Extracellular glutamate concentration in microdialysates from the frontal cortex of mice 4, 12, 18, 24 h after AOM injection, *n* = 4–5, **p < 0.01 ANOVA with Dunnett’s post hoc test

Cortical EEG recording revealed decreased total EEG power, starting from 12 h post-AOM administration (Fig. 8A), with substantial decrease from 100% at baseline to 59% ± 22.62, 56.81% ± 30.45 and 49.71% ± 23.7 in HE mice (*n* = 5) at 12, 18, 24 h post AOM, respectively. A similar pattern of brain activity decline was observed when the EEG spectra were split into separate bands– a decrease of ʘ, α, and β waves became apparent from 12 h post-AOM, whereas γ and, HFO waves decreased starting from 18 h post-AOM (Fig. 8A).

From about 18 h after AOM administration, glutamate levels showed a tendency to increase (without statistical significance), which change persists later, in HE mice we observed substantial increase from 1.64 ± 0.62 µM in control (*n* = 6) to 5.41 ± 2.44 µM in HE mice (*n* = 5) (Fig. 8B).

## Discussion

Morphological changes of astrocytes associated with toxic encephalopathies were discovered in 1912 by Carl Von Hösslin and Alois Alzheimer [32] who analysed post-mortem tissues of a patient with Westphal-Strümpell syndrome, a toxic copper encephalopathy, known today as Wilson’s disease. Ammonium toxicity as a factor underlying HE was described in the early 20th century, after analysing the meat intoxication in Eck fistula dogs [33]. Association between ammonium accumulation in the brain and astrocytic remodelling was first established about 50 years later [34], whereas detailed morpho-functional analysis of the astrocytic changes remained incomplete until present.

In this study, we employed the AOM model of ALF, accepted in acute HE studies, as it sufficiently reproduces neurological and neurobehavioural impairments, as well as the brain oedema [12, 13, 35]. To characterise the disease progression at defined time points, we broadened the morphological and biochemical characteristics of the model and confirmed gradual liver failure after AOM injection.

Here we demonstrate, to our knowledge for the first time, morphological remodelling of astrocytes including retraction of astrocytic leaflets from synapses in the cerebral cortex of mice with HE. Studies of astrocyte architecture performed on HE human brain are similar to complex morphometrical rearrangement observed in HE-affected mouse brain: the number of intersections dropped, and astrocytic arborisation decreased. We also quantified changes in ezrin, PRF1, and AQP4 associated with morphological plasticity [20–22, 26]. Most significantly, in the HE, a substantial decline in AQP4, PFN1, and phosphorylated ezrin, paralleled (and arguably instigated) the atrophy of astrocytic perisynaptic leaflets, suggesting a causal nexus between these phenomena. We provide evidence that the astrocytic remodelling may be linked to dyshomeostasis of neurotransmission, reflected by changes in EEG, and by a substantial increase in extracellular glutamate.

Astrocytic malfunction in the HE was mainly thought to be linked to the brain oedema, which often represents the primary cause of death in this disease [36, 37]. The role of astropathology is, however, wider, as loss of astrocytic homeostatic support and impaired ability of astrocytes to control brain ions, energy, and neurotransmitter homeostasis contribute to synaptic malfunction, neuronal damage, and ultimately to psychiatric and cognitive clinical presentations [38]. Our previous studies established aberrant neurotransmission associated with abnormal calcium buffering in presynaptic terminals [11, 12]. The present study highlights, to our knowledge for the first time, the concurrence of astrocytic structural remodelling and synaptic malfunction in the progression of HE.

Astrocytes actively control neuronal function by instructing synapse formation, plasticity, and remodelling [39–41]. Their essential functions at the synapse are intimately linked to complex astrocytic morphology [42]. From the above perspective, the term primary astrogliopathy, emphasises the key role of astrocytes in the development of HE, similarly to other diseases with a malfunctional astrocyte component [2]. Alzheimer type II astrocytes, although being idiosyncratic to HE, represent only a minor subpopulation of astroglia in the normal and pathological brain.

Since the ramified morphology of astrocytes determines their close structural association with synapses [17], astrocytic morphological plasticity (physiological or pathological) influences synaptic transmission [43, 44]. Morpho-functional aberrations of astrocytes were described in neurodegenerative, neuropsychiatric, and neuromuscular diseases characterised by slow progression [38, 41]. However, in rapidly developing pathologies that primarily affect astrocytes, including HE, neither the details of the remodelling process, nor association with astrocyte dynamics at the synaptic sites of astrocyte-neurone interaction have been hitherto elucidated. In our study, by in depth quantification of astrocyte morphology, we assess the HE cortical landscape from the astrocyte processes remodelling perspective. First, we documented a significant increase in the total astrocyte area of the HE frontal cortex neuropil sections. Ultrastructural analysis agrees well with our previous reports of an increase in the astrocytic area in the HE brain, reflecting changes in astrocyte morphology associated with swelling [11]. We further quantified the areas occupied by perivascular astrocytes vs. astrocyte branches localised at a distance from the vessels (brain neuropil). Irrespective of whether we quantify astrocytic endfeet *in toto* or their branches and leaflets, HE is linked with a significant increase in the territory occupied by astrocytes. Enlarged astrocyte territory was noted in brains of HE patients as well.

Further, to address astrocyte complexity, we performed an immunofluorescence analysis of the distribution of astrocytic processes. The astrocytic volume fraction (VF) is an indirect measure of astrocyte leaflets number and average size in a tissue section. We found a noticeable decrease in the leaflets VF starting from around 15 μm distance from soma in HE mice. At the same time, the VF closer to the soma was higher compared to control.

Astrocytic structural remodelling occurs in response to different stimuli (e.g., osmotic stimulation, stress, neuronal activity, etc.). Changes in plasticity-related proteins AQP4, the phosphorylated protein ezrin, and PFN1, developed in parallel with astrocyte morphology changes. Astrocyte sensitivity to the osmotic changes results from their ability to rapidly take up excessive water and ions through AQP4 and associated ionic channels. Indeed, the involvement of AQP4 in the brain volume changes in HE was often [23], albeit inconsistently [45] reported. We found a decrease in AQP4 protein, phosphorylated ezrin, and PFN1 in HE brains. Both PRF1 and ezrin are required for the morphological plasticity of leaflets [19, 46]. Translocation of AQP4 within the astrocyte membrane near adjacent neurones may indirectly affect extracellular levels of neurotransmitters by changes in extracellular volume during synaptic activity. In line with the above, the variations in the expression of mRNA coding for the M23 isoform may reflect, or contribute to, HE-induced astrocytic adaptive change in astrocyte morphology. Substantial fluctuations in M23 isoform transcripts may arise from compensatory attempts to restore the expression of M23 clusters, engaged in glutamatergic synapse support [26]. Leaflet atrophy, observed in this study, translates into reduced synaptic coverage, which potentially may affect neurotransmission. Quantification of astrocytic leaflets surrounding the perimeter of cortical synapses demonstrated a lower number of synapses in close contact with a single astrocytic leaflet, in favour of contact with two leaflets. Further, distances between the synaptic active zone and the closest astrocytic membrane increased by ~ 25% in HE. Increase in the number of leaflets contacting synapses may be a compensatory response to aberrant neuronal activity aimed at more effective astrocyte-neuron communication or is a consequence of astrocyte failure in response to swelling. Similar astrocytic atrophy was discernible in astrocytes in post mortem from brains of HE patients. The average length of the branches was similar however, the average number of intersections was reduced in HE brain indicating limited astrocytic interaction with other components of the brain’s active milieu, including synapses. Synapses in the HE brain, therefore, have fewer astrocyte associations, which may compromise proper homeostatic support, facilitate the spillover of neurotransmitters, and affect synaptic transmission.

Reduced EEG activity is a consistent feature of advanced, symptomatic HE in human patients [47] and experimental animals [48–50], reflecting decreased synaptic transmission. Previous studies highlighted the inefficient recruitment and/or impaired trafficking of synaptic vesicles to synaptic active zone as one of the possible molecular mechanisms underlying decreased neurotransmission in the HE [12, 51].

Increase of extracellular glutamate concentration measured in the brain microdialysates of HE rats is consistent with findings in human HE patients [52] and different rodent HE models [48, 53, 54]. Reduction of EEG power with increased extracellular concentration of glutamate has been reported earlier in another animal HE models [48, 55]. Glutamate spillover frequently observed in HE is related to the deficiency of astrocytic glutamate uptake observed in hyperammonemic conditions [56]. Accumulation of extracellular glutamate due to astrocytic homeostatic failure impairs neurotransmission, affects neuronal excitability, and instigates excitotoxicity.

Gradual decrease in EEG power across all frequency bands, typical for metabolic encephalopathies, indicates a global reduction in neuronal activity and/or a decrease in the synchronization of neural oscillations. Acute symptomatic seizures, observed in one-fifth of HE patients, result not from epileptic discharges but rather from electrolyte imbalance (hyponatremia, hypocalcemia, hypomagnesemia) [57]. Previous electrophysiological recordings, revealing aberrant neuronal activity and synaptic function in the AOM model, reconcile the increased interstitial Glu level and a decreased EEG power mirrored by progressing decline of the neurological status, assessed in mice by scoring the corneal-, pinna-, vibrissae-, startle-, righting- and postural reflexes [12].

Here we demonstrated unique complex astrocytic changes in HE brain; in addition to an overall increase in the volume of astrocytes at the level of soma and primary branches, most likely reflecting astrocyte oedema, we found decreased complexity of astrocytic arborisation manifested by atrophy of distal branches and leaflets. These morphological changes, which are remarkably rapid (to compare, reactive astrogliosis in response to brain trauma is initiated 24–36 h after the insult [38], are associated with reduced synaptic coverage and arguably loss of astrocytic homeostatic support. The bimodal nature of astrocytic aberrations reflects the predicted role of these cells in the two major clinical manifestations of HE. Swollen astrocytic cell body is a prerequisite of brain oedema, while atrophy of processes contacting neurons is consistent with impaired neurotransmission.

Our study includes human cortical tissue from individuals within a range of ages corresponding to a period during which age-related astrocytic changes are well documented [58]. Given that aging is known to affect astrocyte morphology and function, it might be expected that, acting additively, both factors, ageing and HE, might synergistically potentiate changes in the morphology of astrocytes towards a more atrophic state. However, the nature of both changes might not be similar. Brain ageing is associated with impaired astrocytic morphology and mitochondrial malfunction, but not with neuronal excitability and changes in inhibitory signalling. In turn, neurotransmission changes observed during the progression of HE parallel astrocytic morphology rearrangement. Finally, astrocytic oedema not reported in physiological ageing bespeaks for distinctive astrocytic malfunction in HE, being a novel factor that requires careful interpretation.

The obvious limitation of this study is that the morphometry was performed only in one brain region whereas other brain regions could present different dynamics. We cannot exclude heterogeneity of HE effects on other cortical and subcortical regions. In addition, the brain’s active milieu consists of multiple cell types, and non-cellular elements (i.e. compromised blood-brain barrier, cerebrovascular impairment, cerebral blood flow dysregulation), which may affect (and be affected) HE by mechanisms extending beyond astrocytic morpho-functional remodelling. Since complex interaction of all components is critical for brain homeostasis, the malfunction of one element may initiate a cascade of deleterious processes. Hence, further studies evaluating the HE-related changes in particular of elements of the brain-active milieu and their causal relationships are needed.

## Conclusions

In the present study, we demonstrated that cortical astrocytes in the HE induced by ALF undergo complex morphological alterations. We described, for the first time, simultaneous volume increase (oedema) of astrocyte soma and principal branches and retraction of perisynaptic leaflets. The soma and primary branches swell, whereas complexity of distal arborisation is reduced, mainly by rapid atrophy of distal branches and leaflets. This results in the reduced astrocyte synaptic coverage that translates into decreased neuronal activity. Thus, rapid emergence of aberrant astrocytes presents the fundamental element in the pathogenesis of the HE.

## Electronic supplementary material

Below is the link to the electronic supplementary material.

Supplementary Material 1

Supplementary Material 2

Supplementary Material 3

## Data Availability

No datasets were generated or analysed during the current study.

## Declarations

### Abbreviated summary

Hepatic encephalopathy is associated with astrocyte remodelling manifested by swelling of the soma and primary branches together with atrophy of distal branches and leaflets; the latter retract from synapses, thus affecting neurotransmission and contributing to the reduced neuronal activity. Astrocyte remodelling was linked to a decrease of plasmalemma-cytoskeleton linker phosphorylated ezrin (Phos-ERM), actin modulator profilin-1 (PFN1), and water channel aquaporin 4 (AQP4).

### Additional materials

Additional materials are available at *Acta Neuropathologica Communications* online.
