# Supplementary material for: Oedematic-atrophic astrocytes in hepatic encephalopathy
Source: Acta Neuropathol Commun. 2025 May 31;13:122. doi: 10.1186/s40478-025-02045-5 (PMC12125882; doi:10.1186/s40478-025-02045-5)

Ezrin western blot (1 and 2) (~70-80kDa):

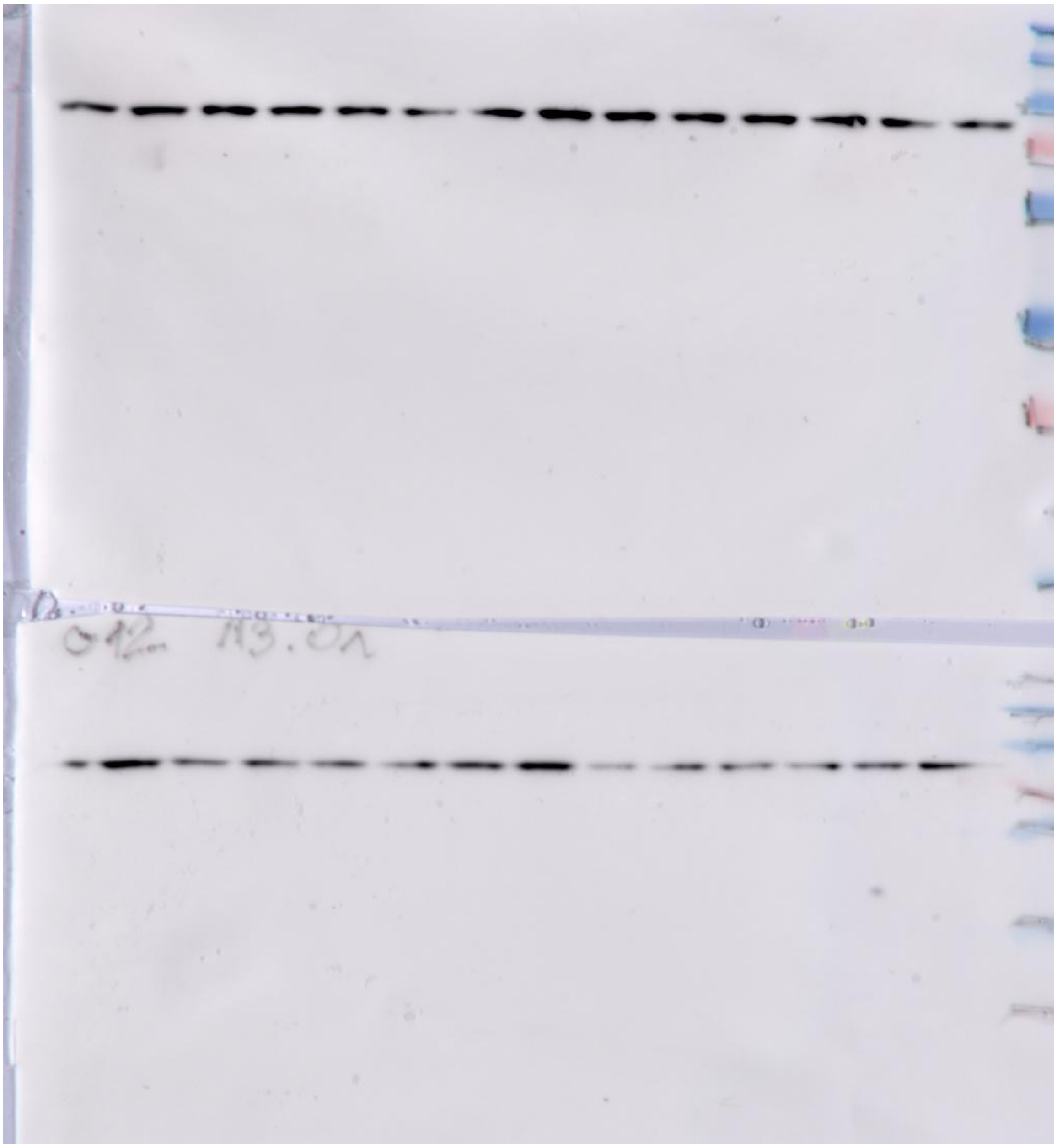

Figure:

Upper image, From right: 3 control, 3 4h HE, 2 12h HE, 3 18h HE, 3 24h HE

Below image, from right: 3 control, 3 4h AOM, 3 12h HE, 2 18h HE, 3 24h HE

GAPDH to Ezrin blots (1 and 2); marker (upper), bands (below):

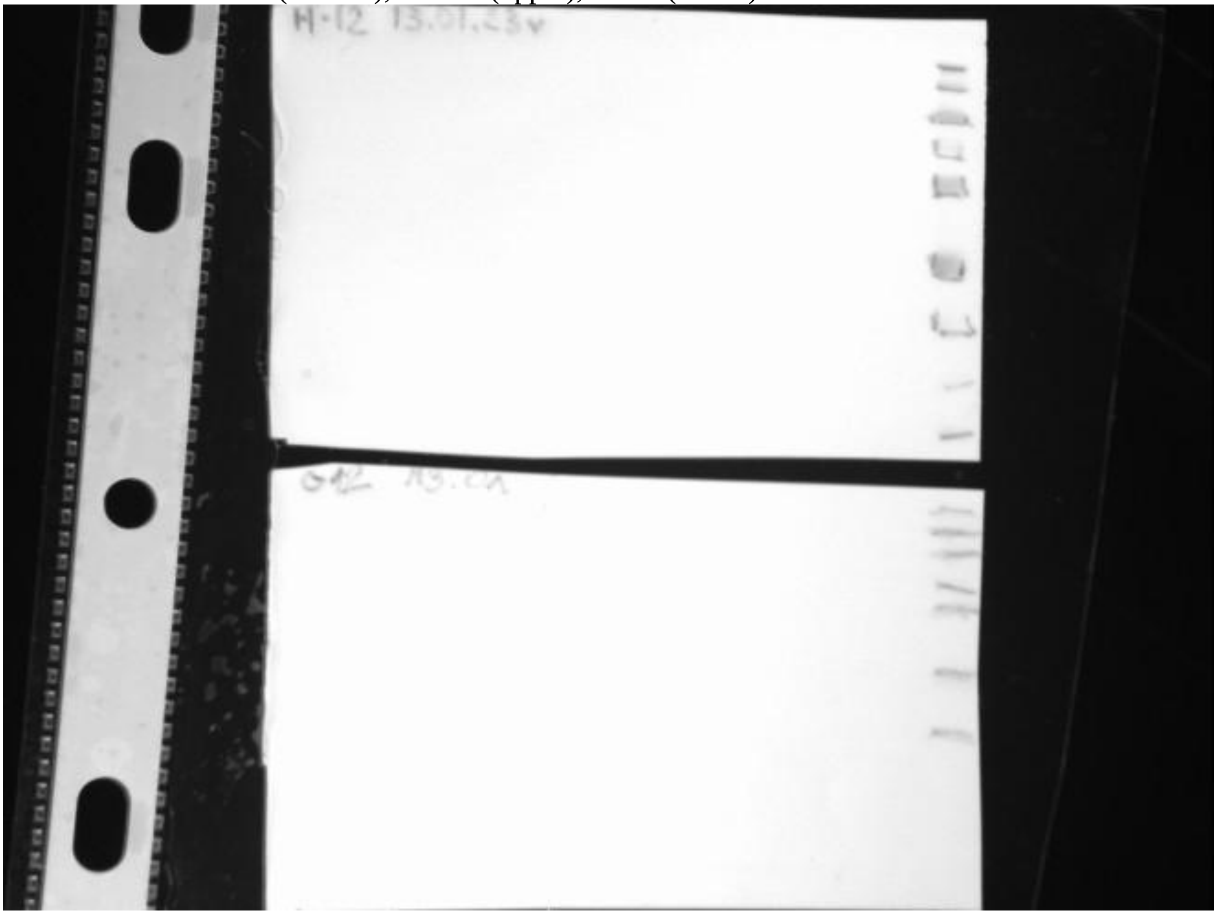

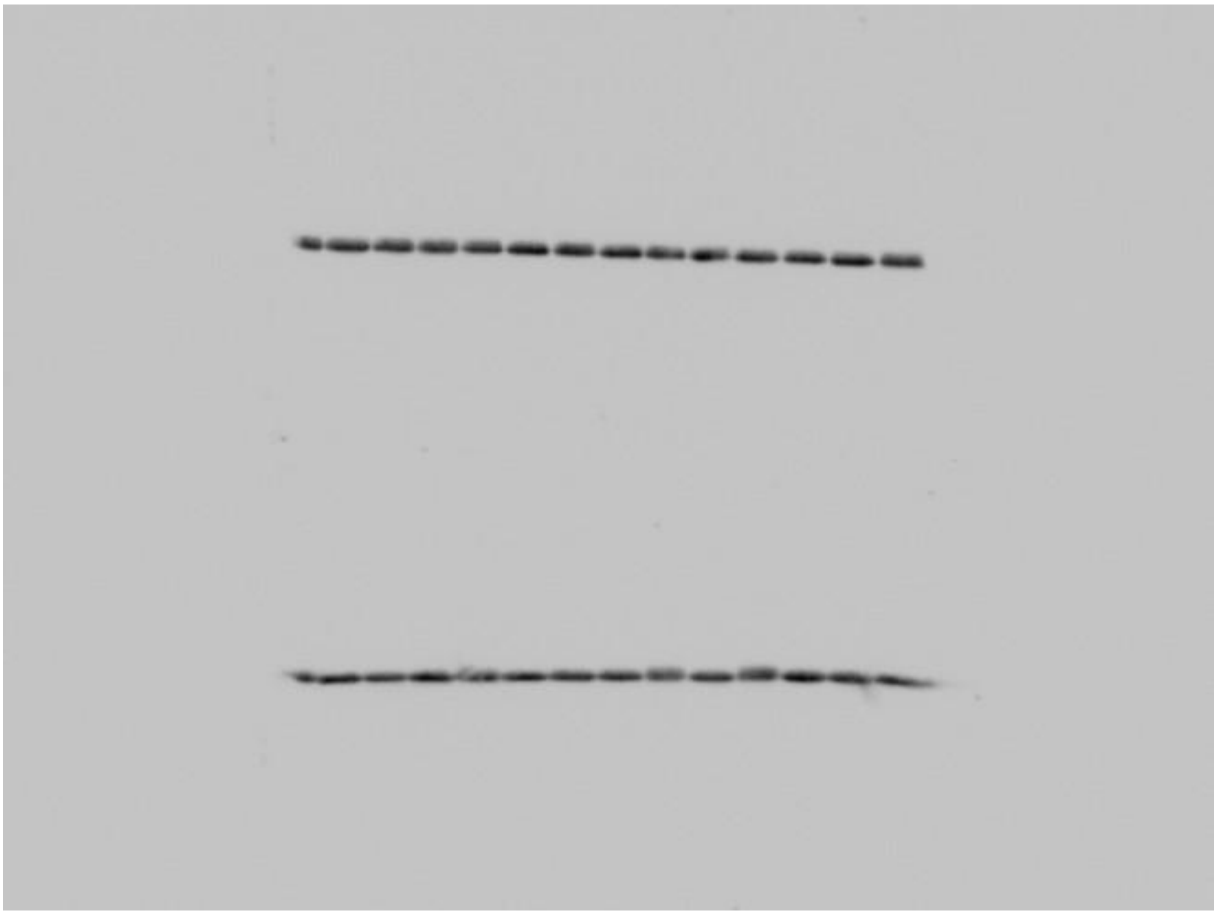

Ezrin western blot 3 (~70-80kDa); below

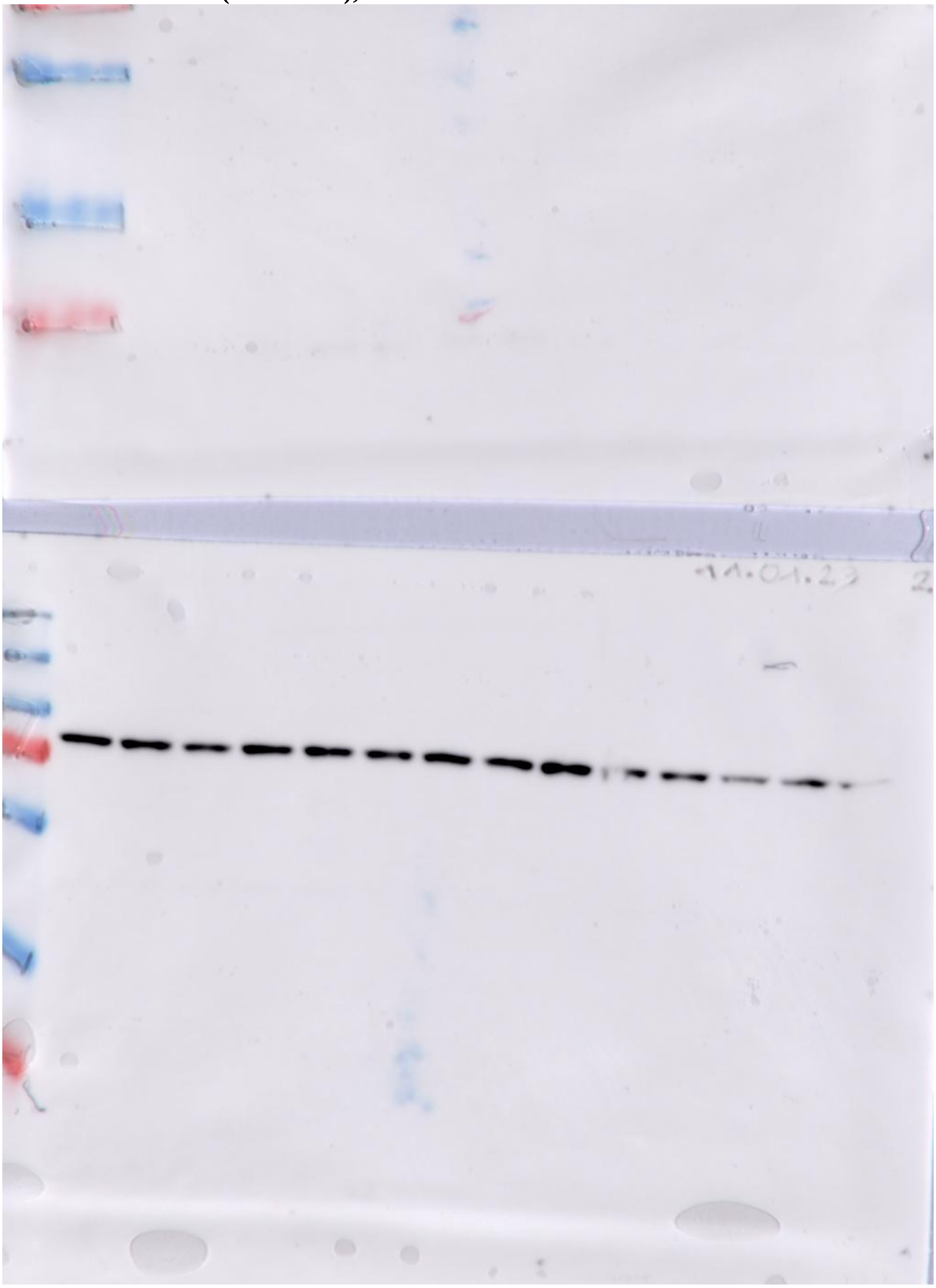

Figure:

Below image from left: 3 control, 2 4h HE, 3 12h HE, 3 18h HE, 3 24h HE

GAPDH to Ezrin blot 3; marker (upper), bands (below):

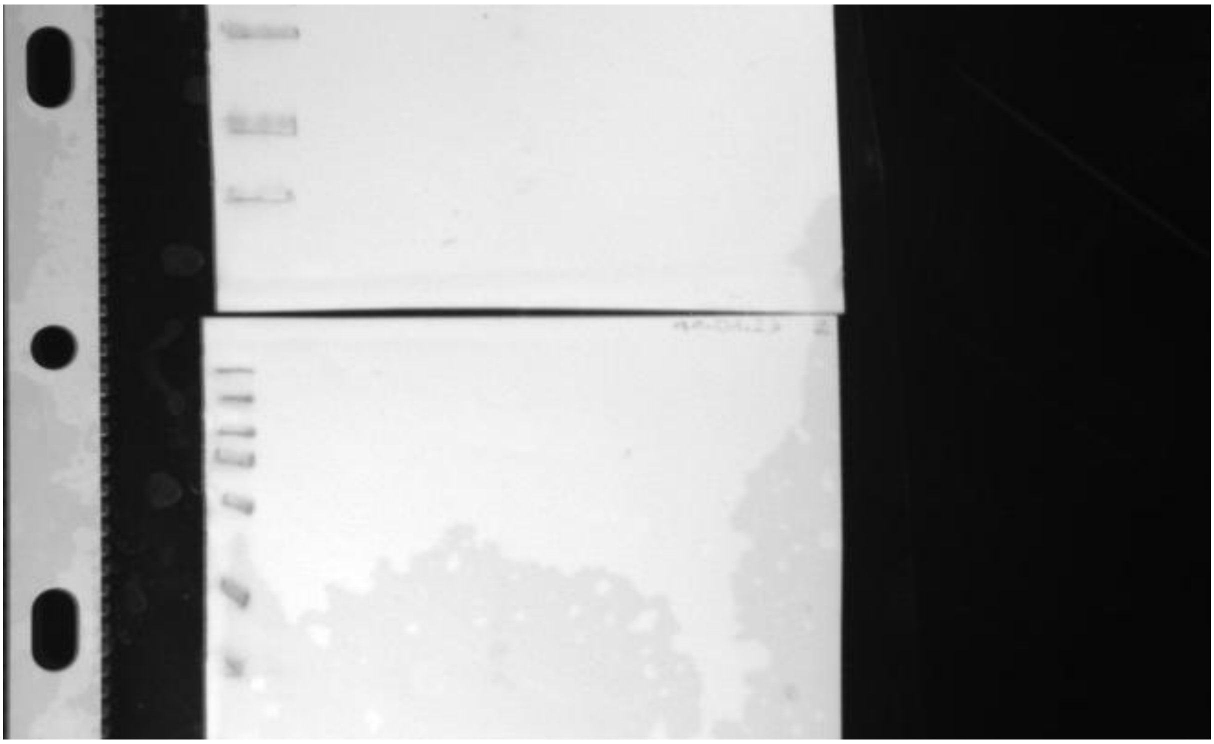

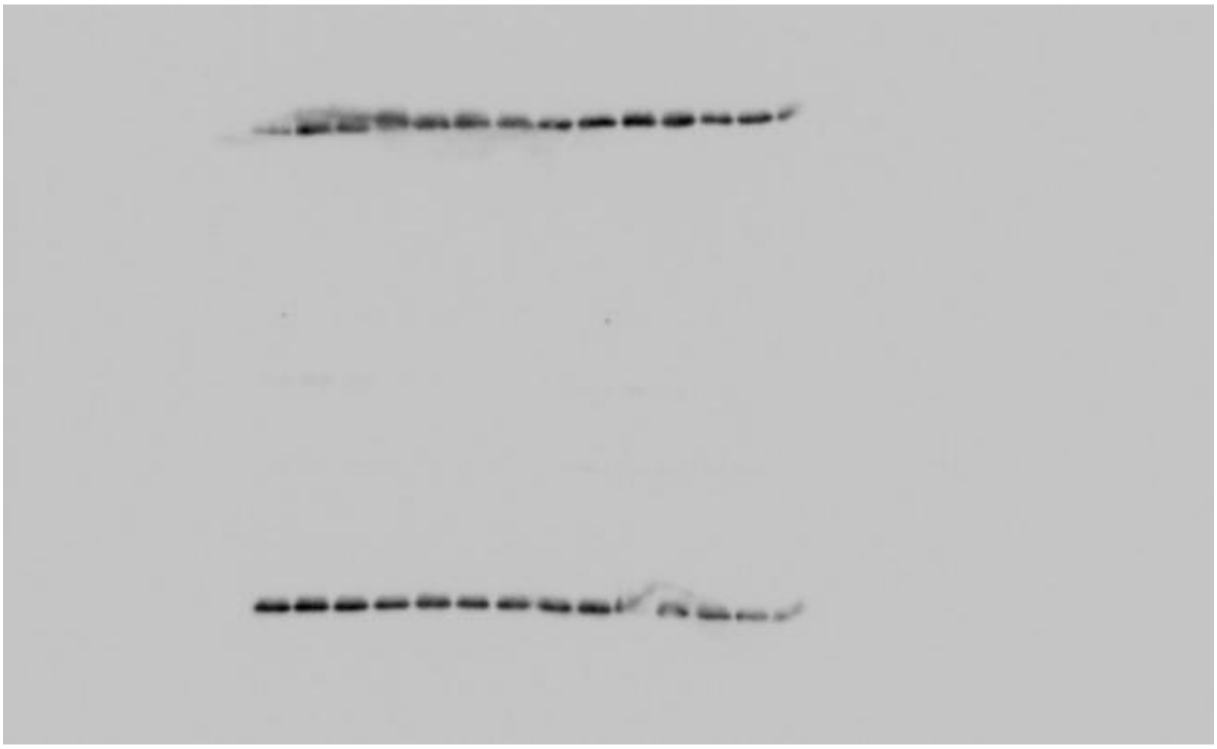

Profilin 1 (1) (~15kDa):

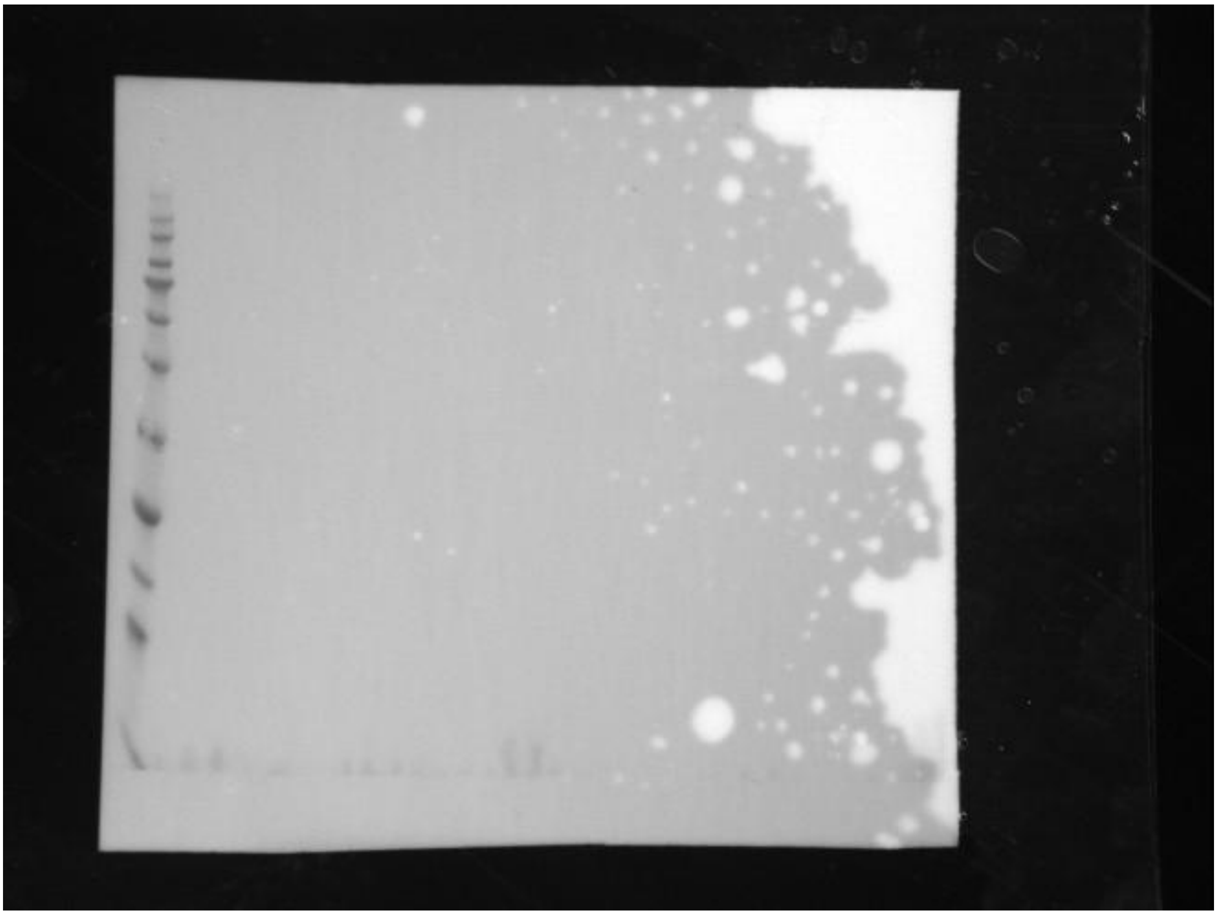

Proffilin 1 (1) signal:

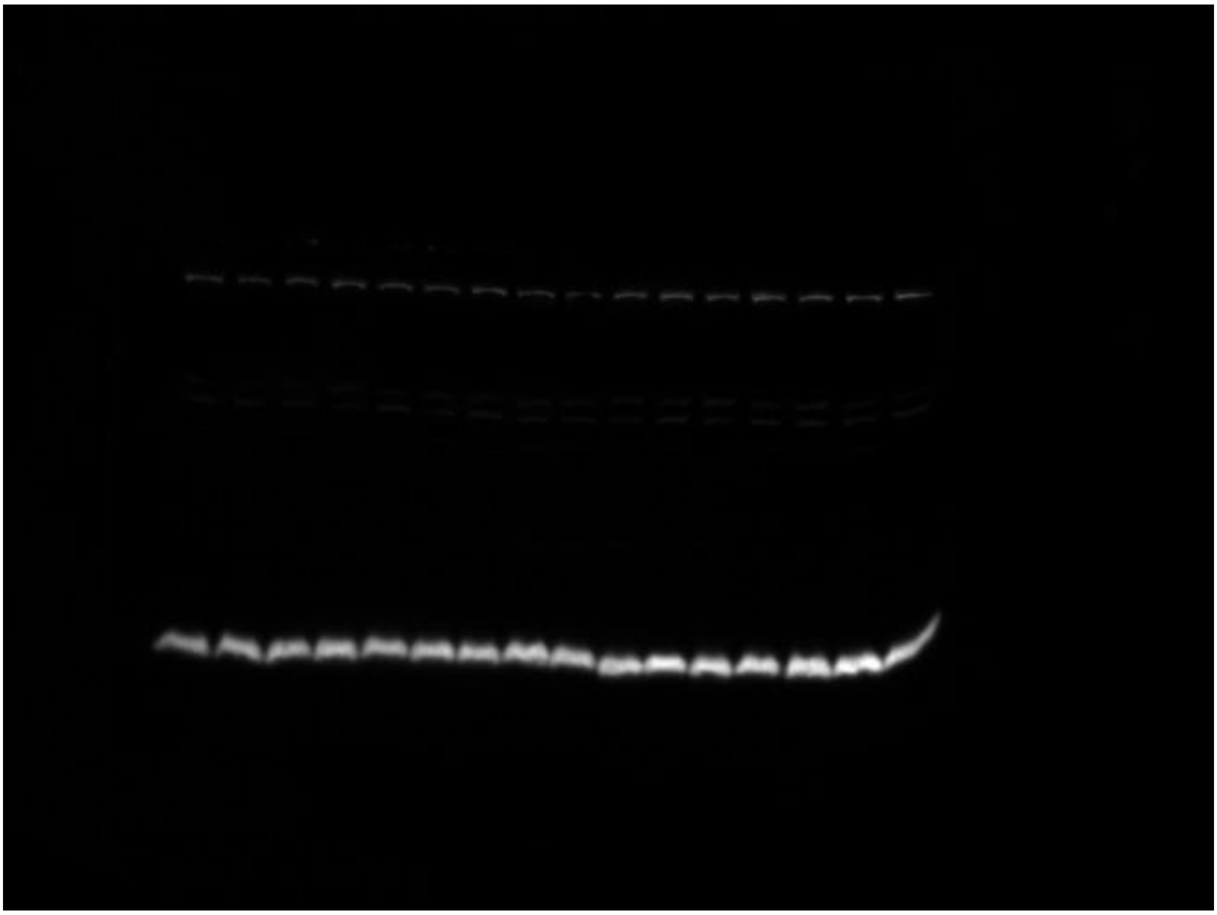

From Right: 4 control, 3 4hAOM, 3 12hAOM, 3 18hAOM, 3 24hAOM

GAPDH to Profilin 1 (1); marker (upper), bands (below):

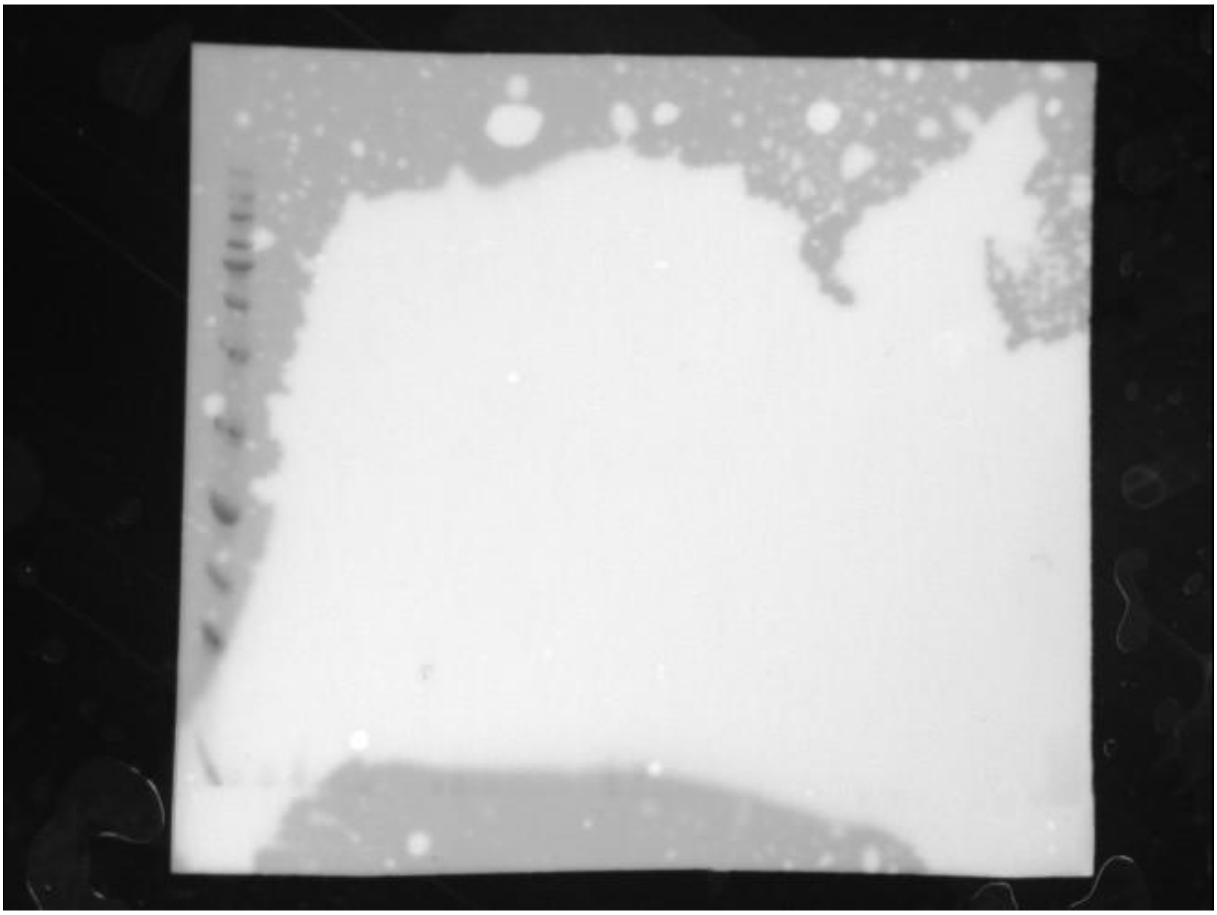

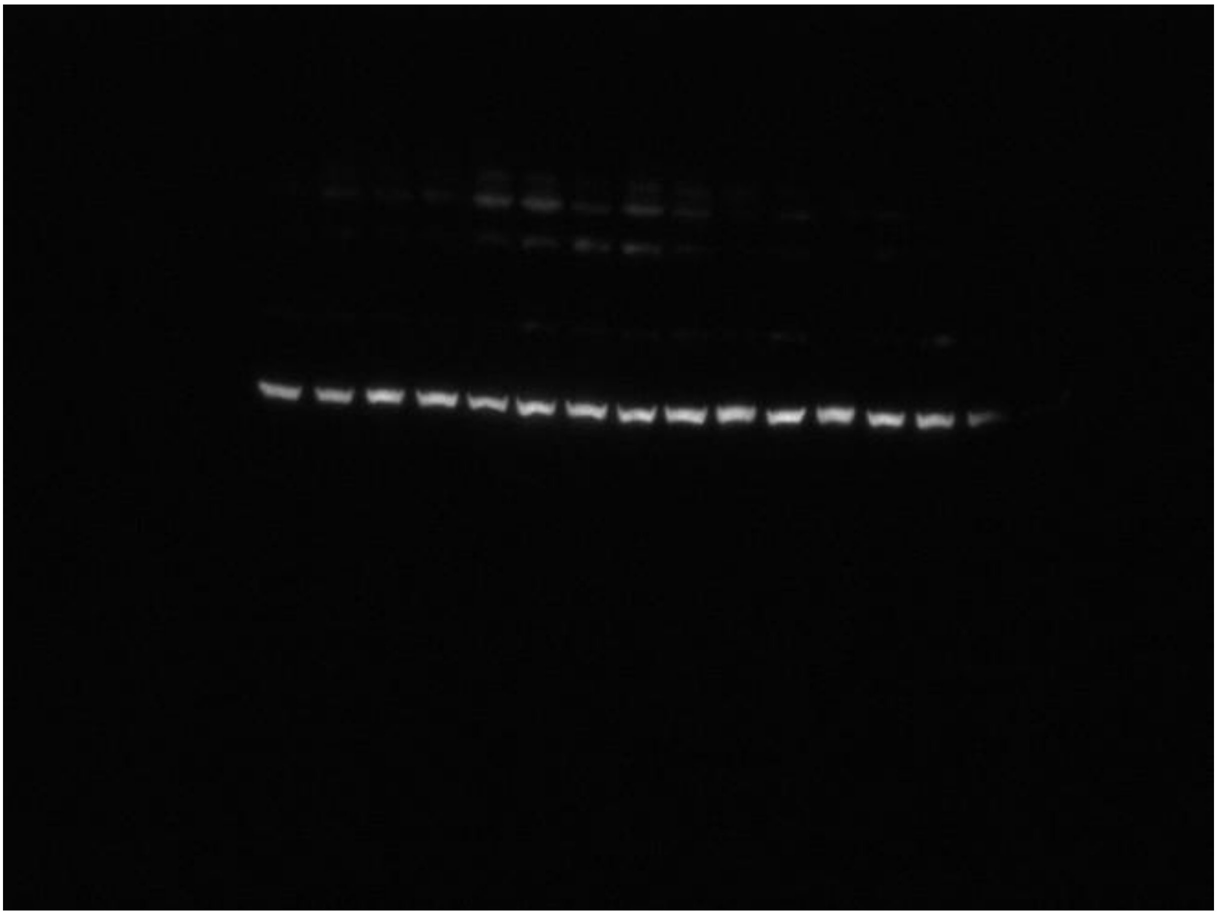

From right: From Right: 3 control (out of first control), 3 4hAOM, 3 12hAOM, 3 18hAOM, 3 24hAOM

Profilin 1 (2) (~15kDa):

Proffilin 1 (2) marker

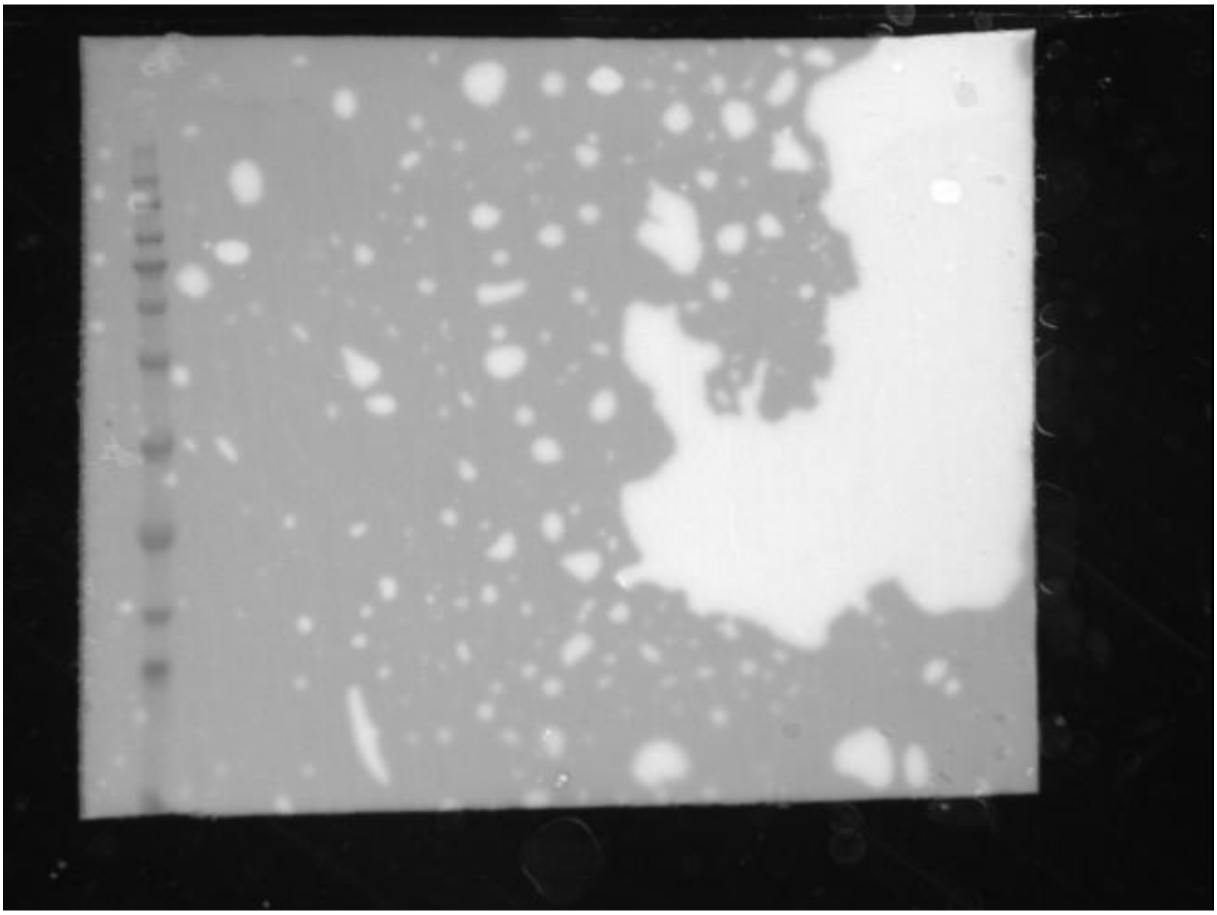

Profilin bands:

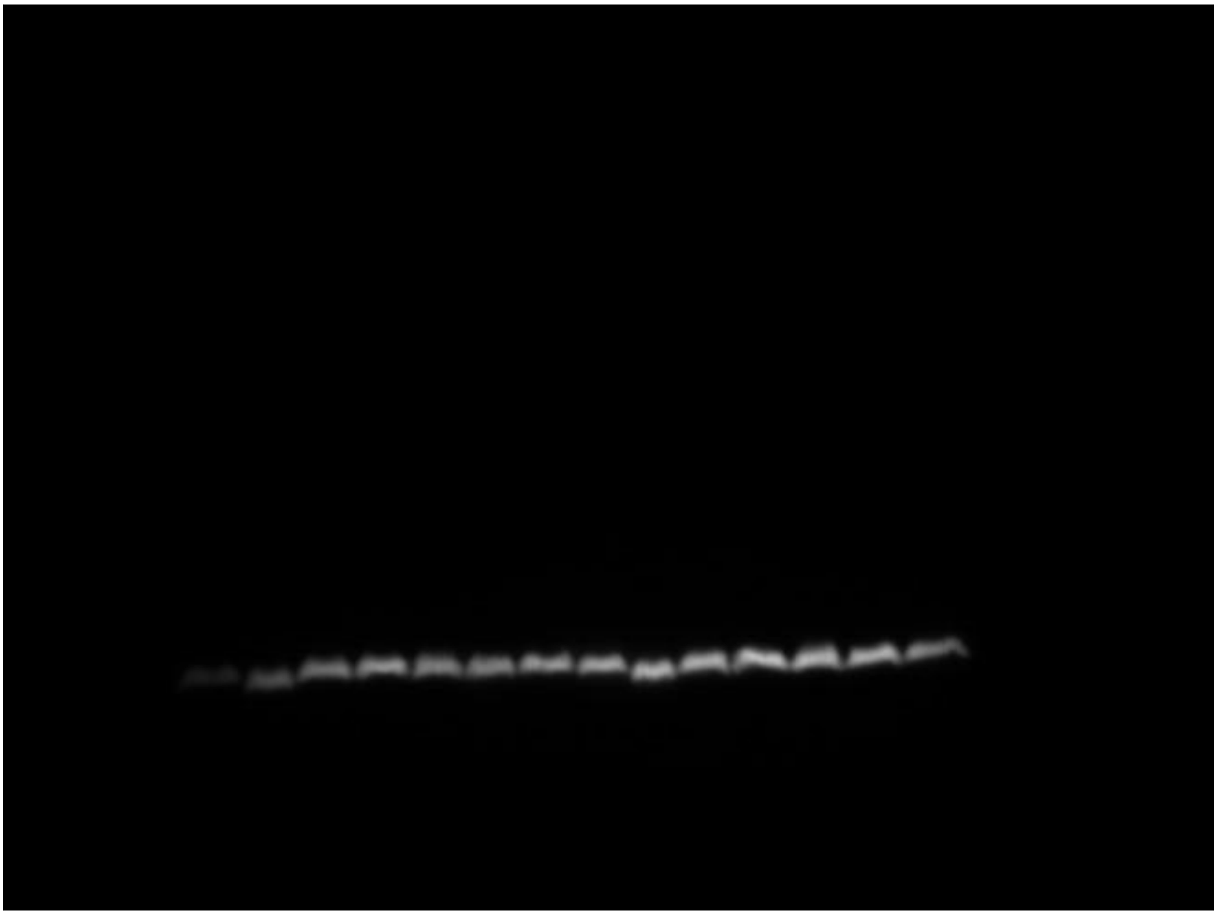

From Right: 3 control, 3 4hAOM, 2 12hAOM, 3 18hAOM, 3 24hAOM

GAPDH to Profilin 1 (2); marker (upper), bands (below):

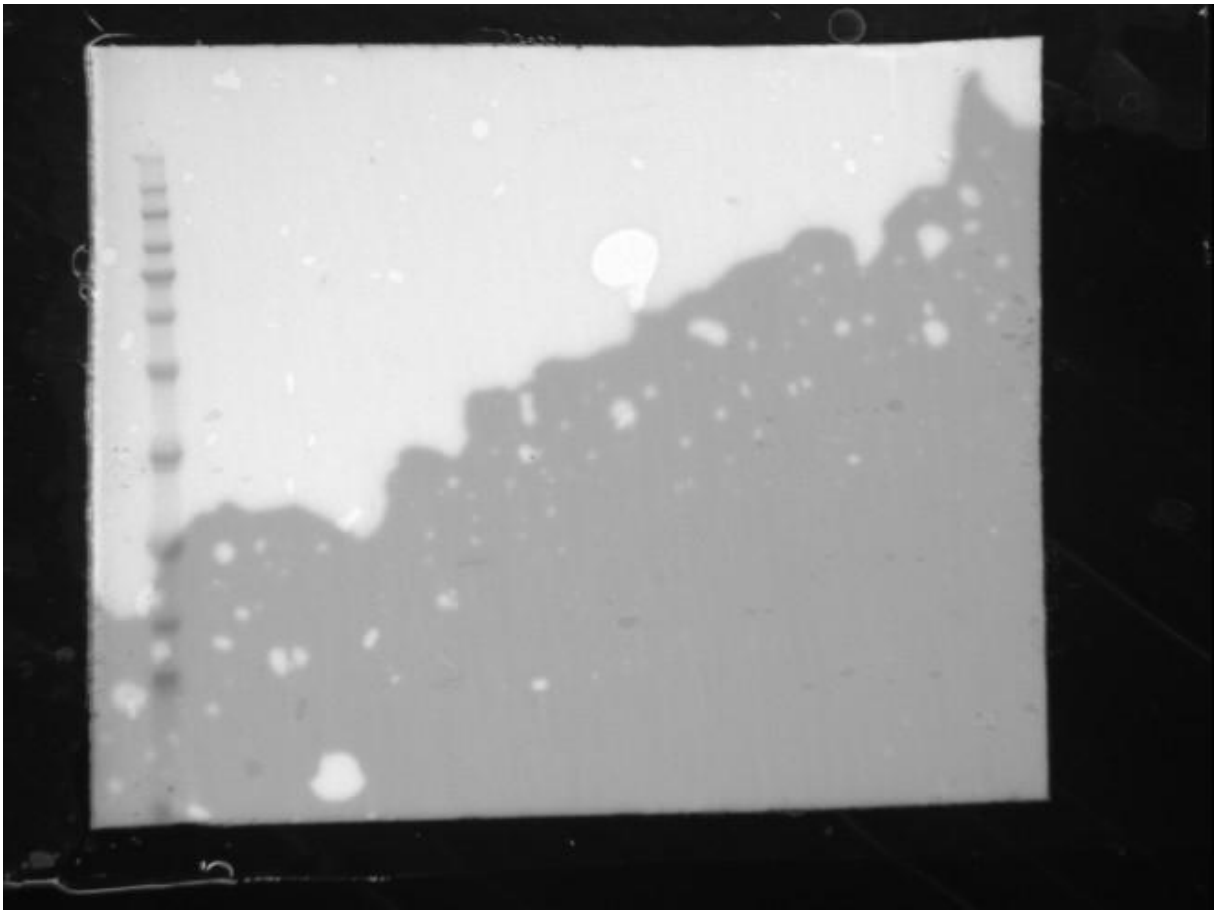

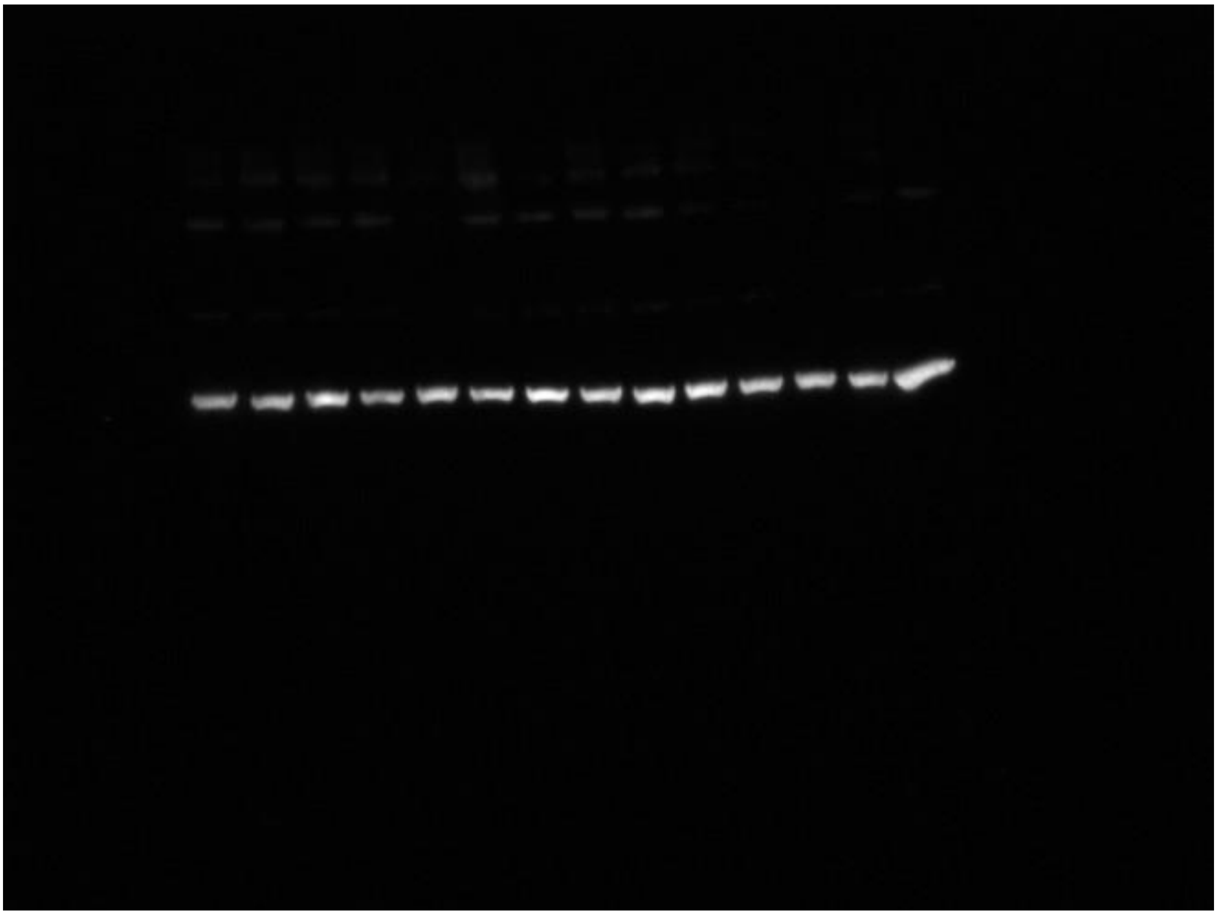

Profillin 1 (3) (~15kDa) (marker):

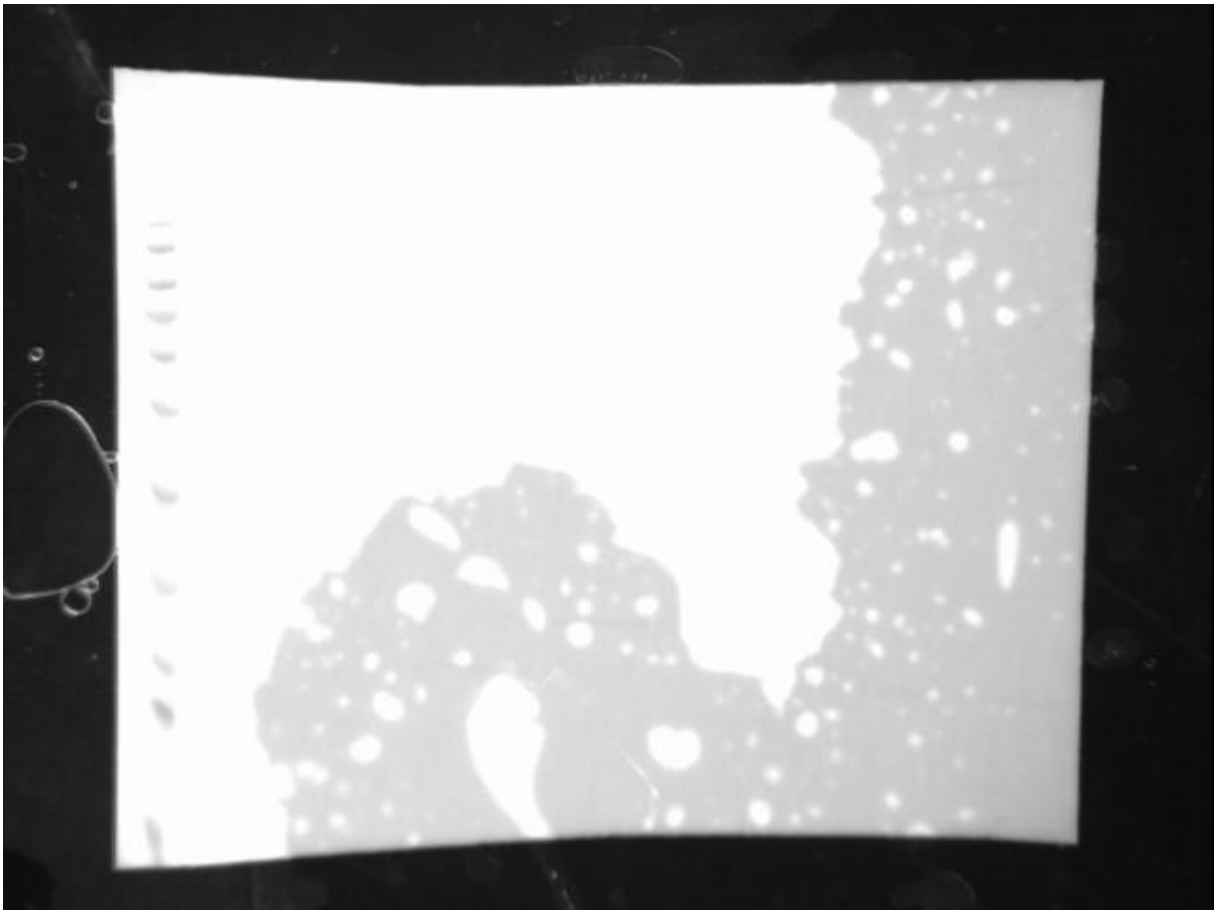

Profilin 1 (3) bands:

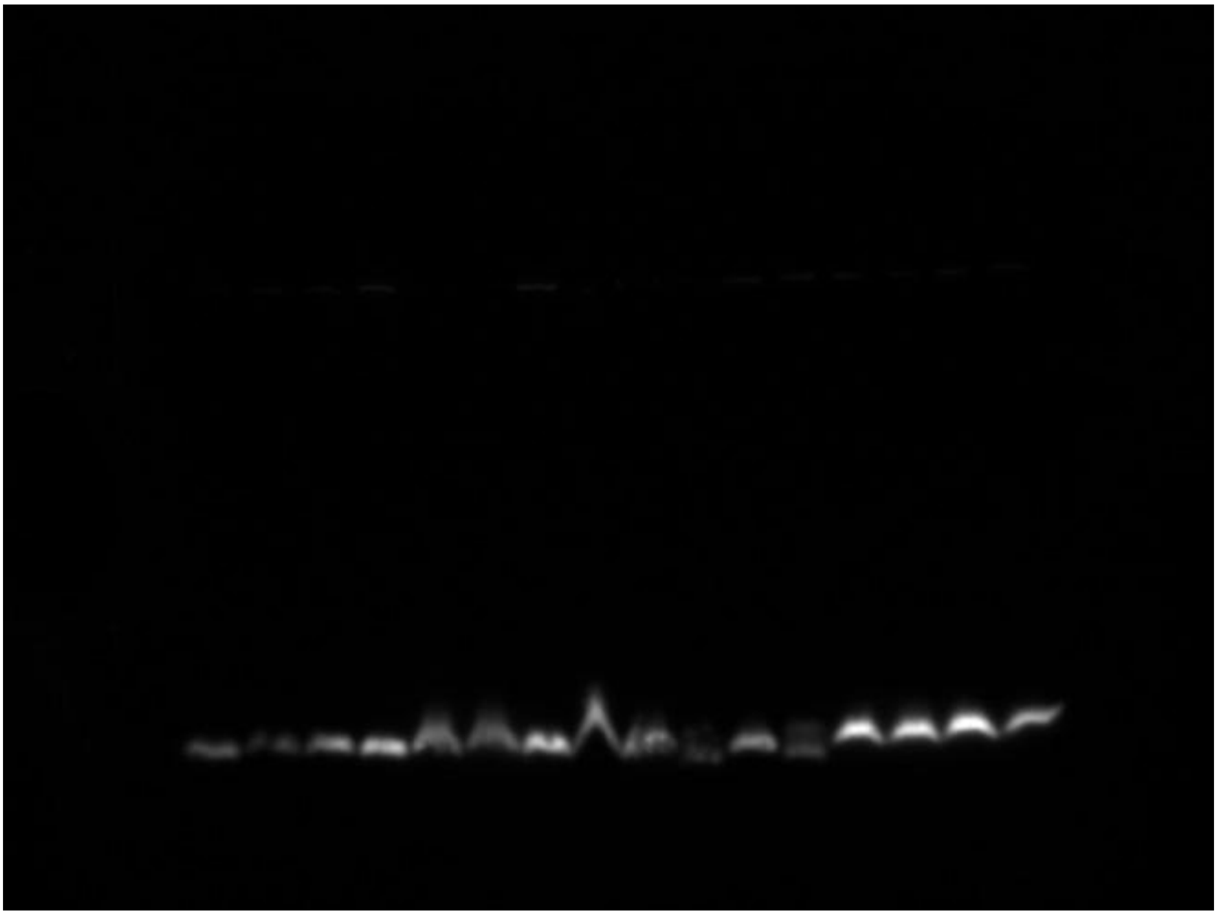

From right: 4 control, 3 4hAOM, 3 12hAOM, 3 18hAOM, 3 24hAOM

GAPDH to Profilin 1 (3); marker (upper), bands (below):

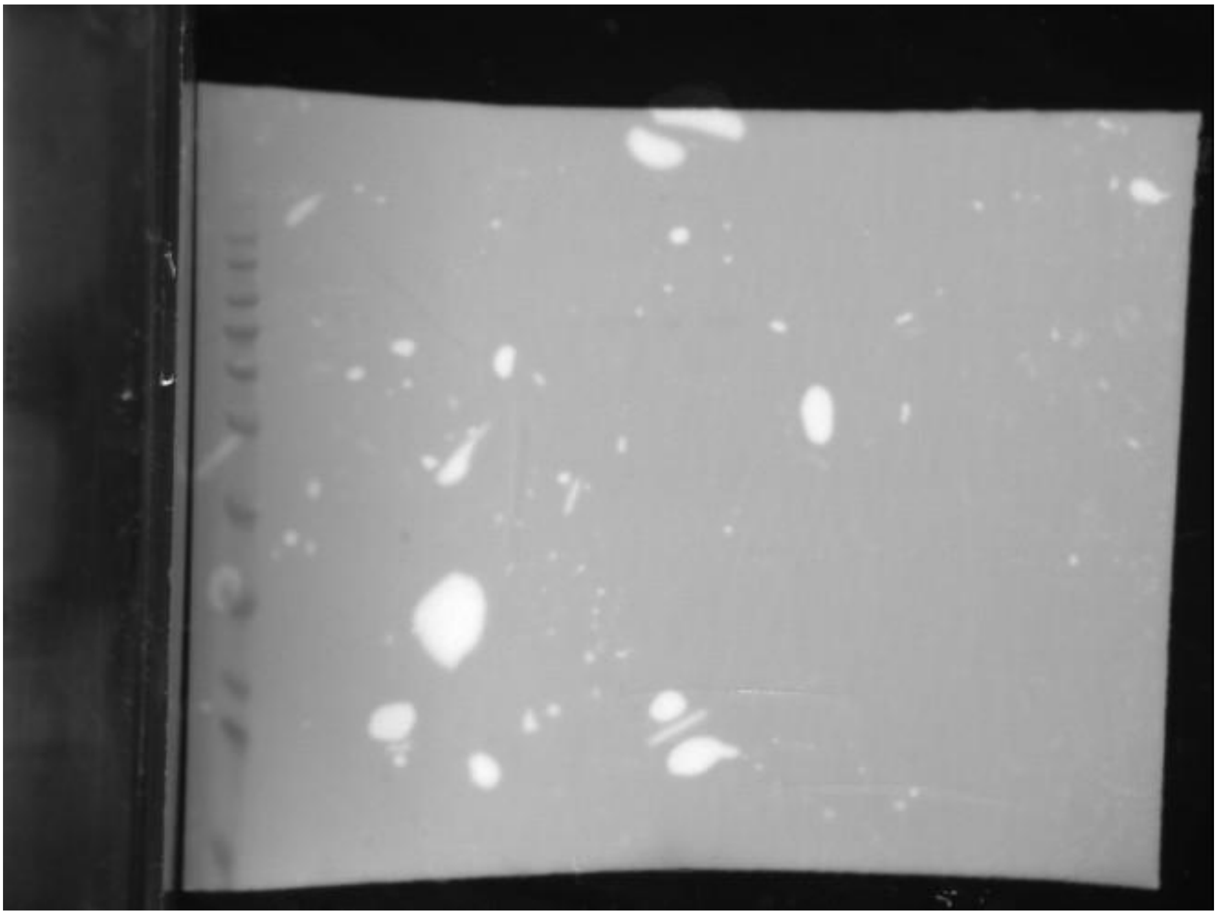

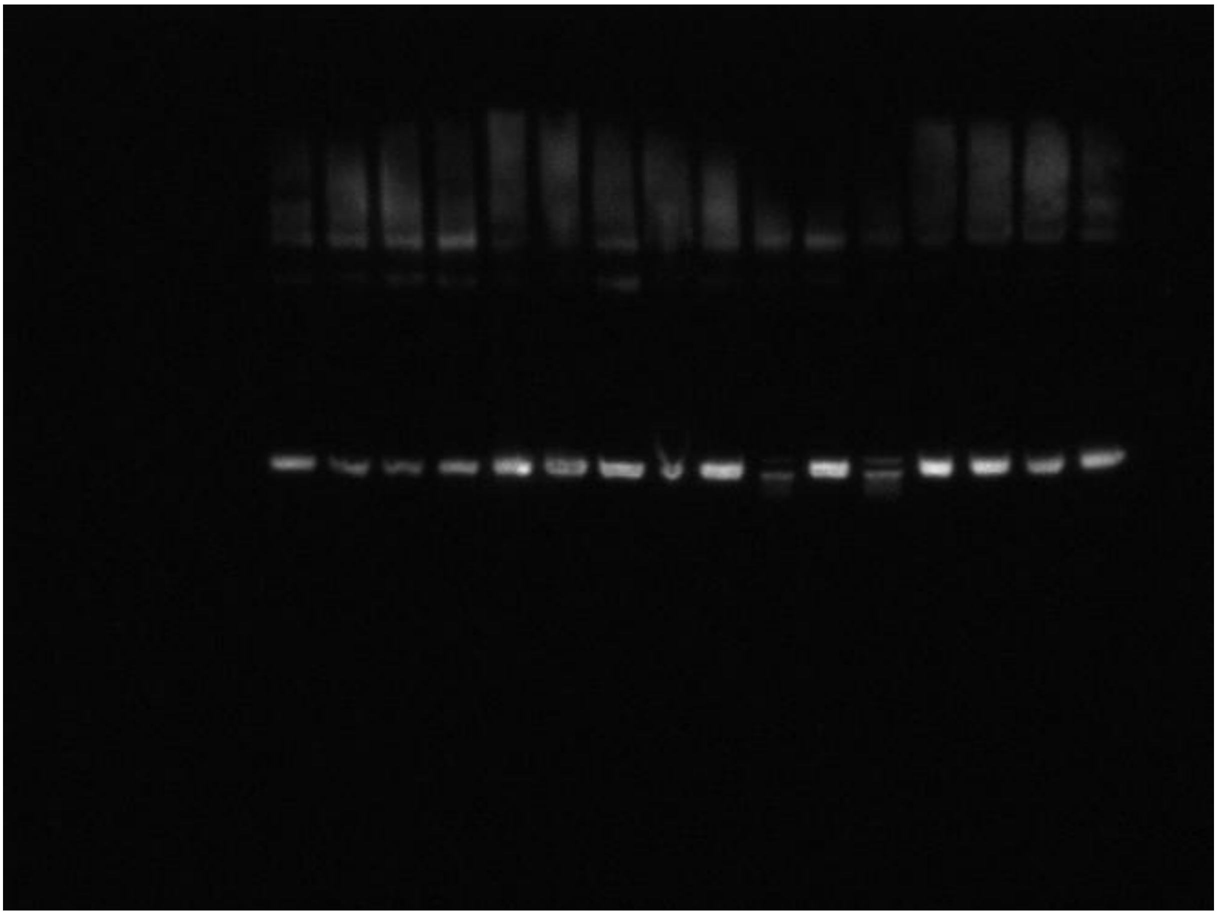

Profilin 1 (4) (~15kDa)

Profilin marker:

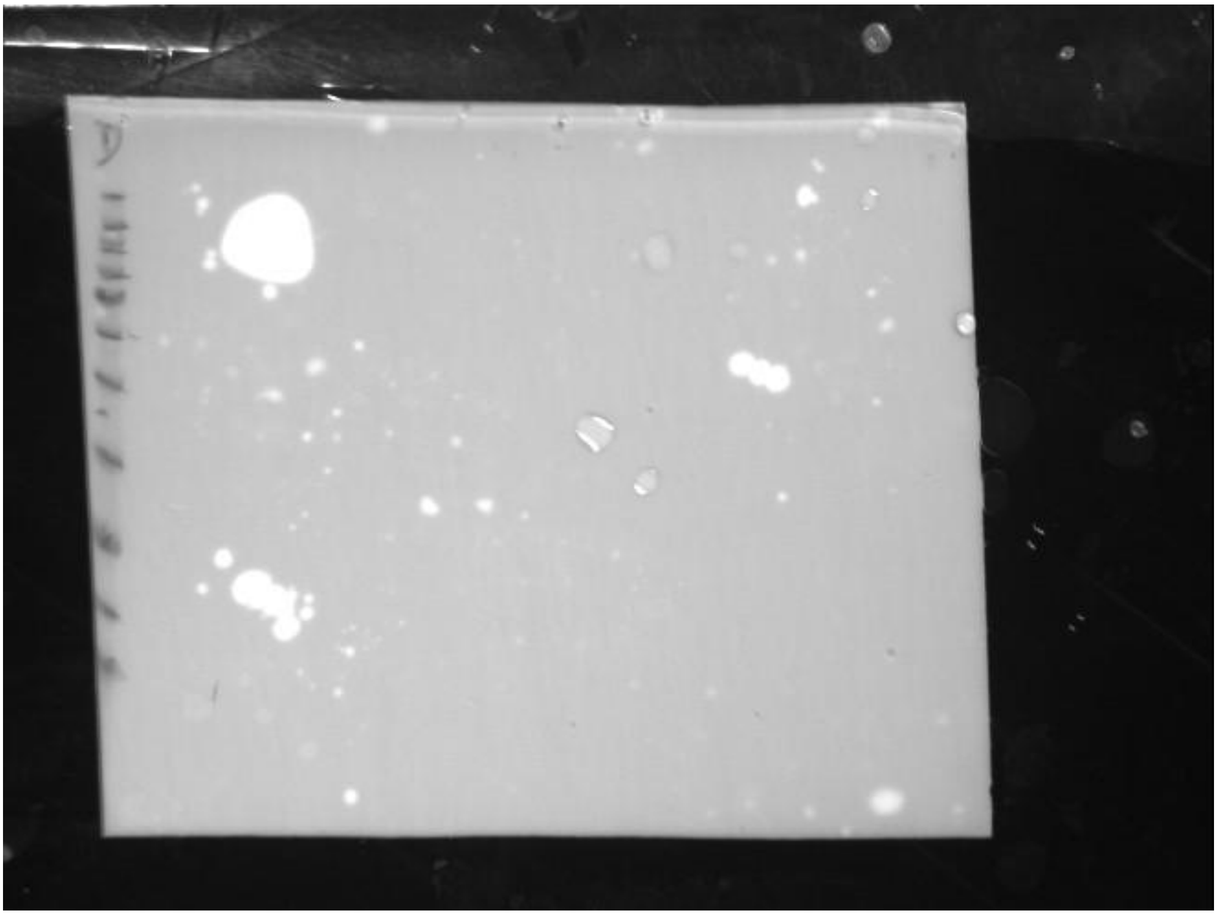

Profilin 1 (4) bands:

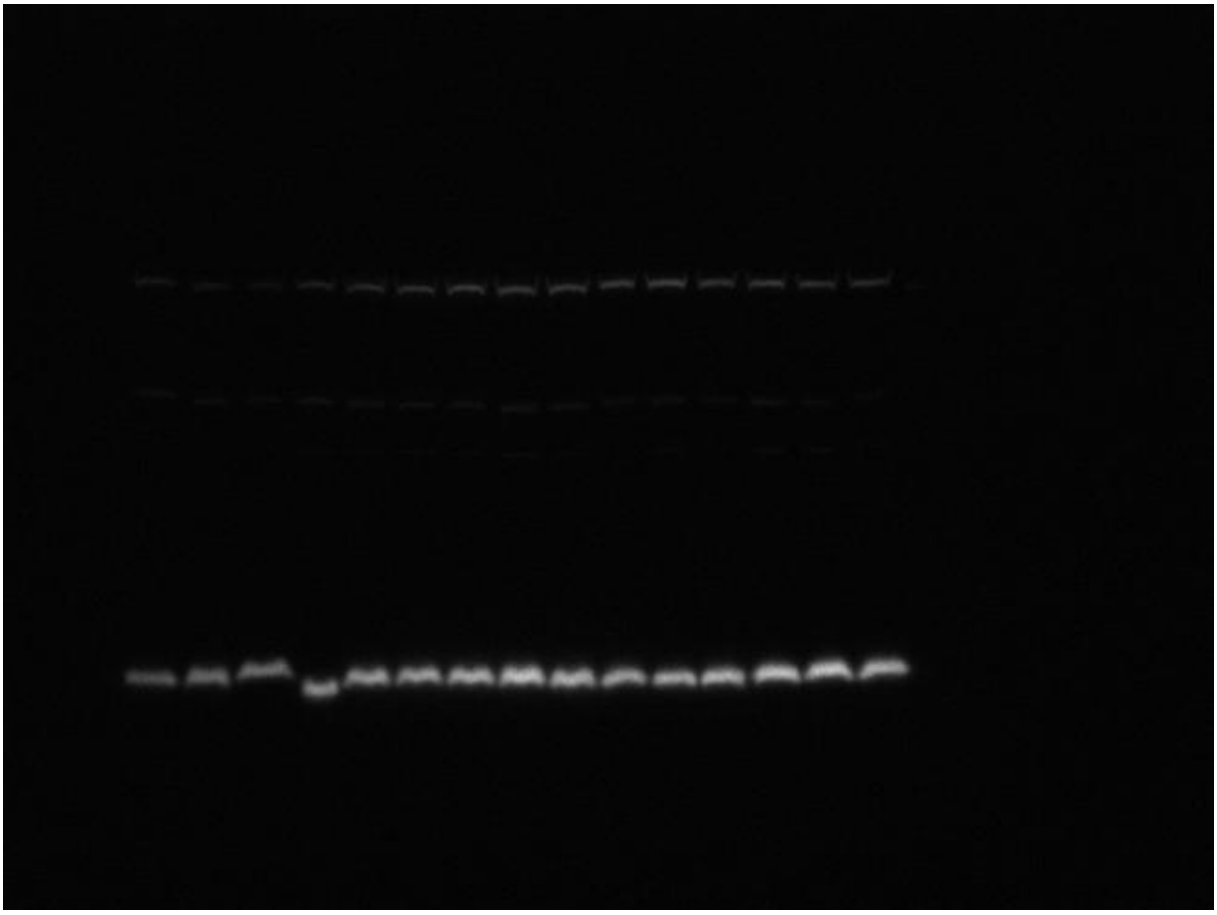

From right: 3 Control, 3 4h HE, 3 12h HE, 3 18h HE, 3 24h HE

GAPDH to Profilin 1 (4); marker (upper), bands (below):

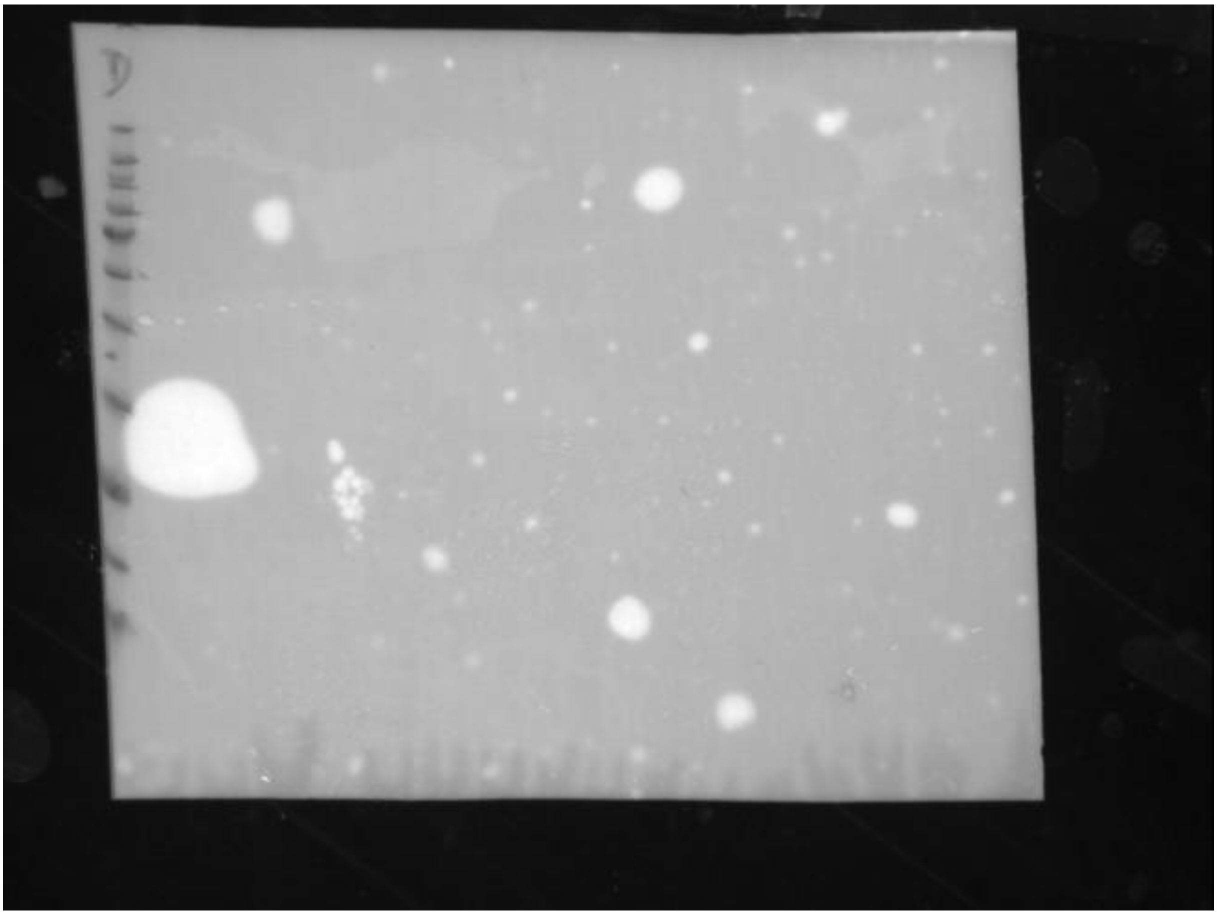

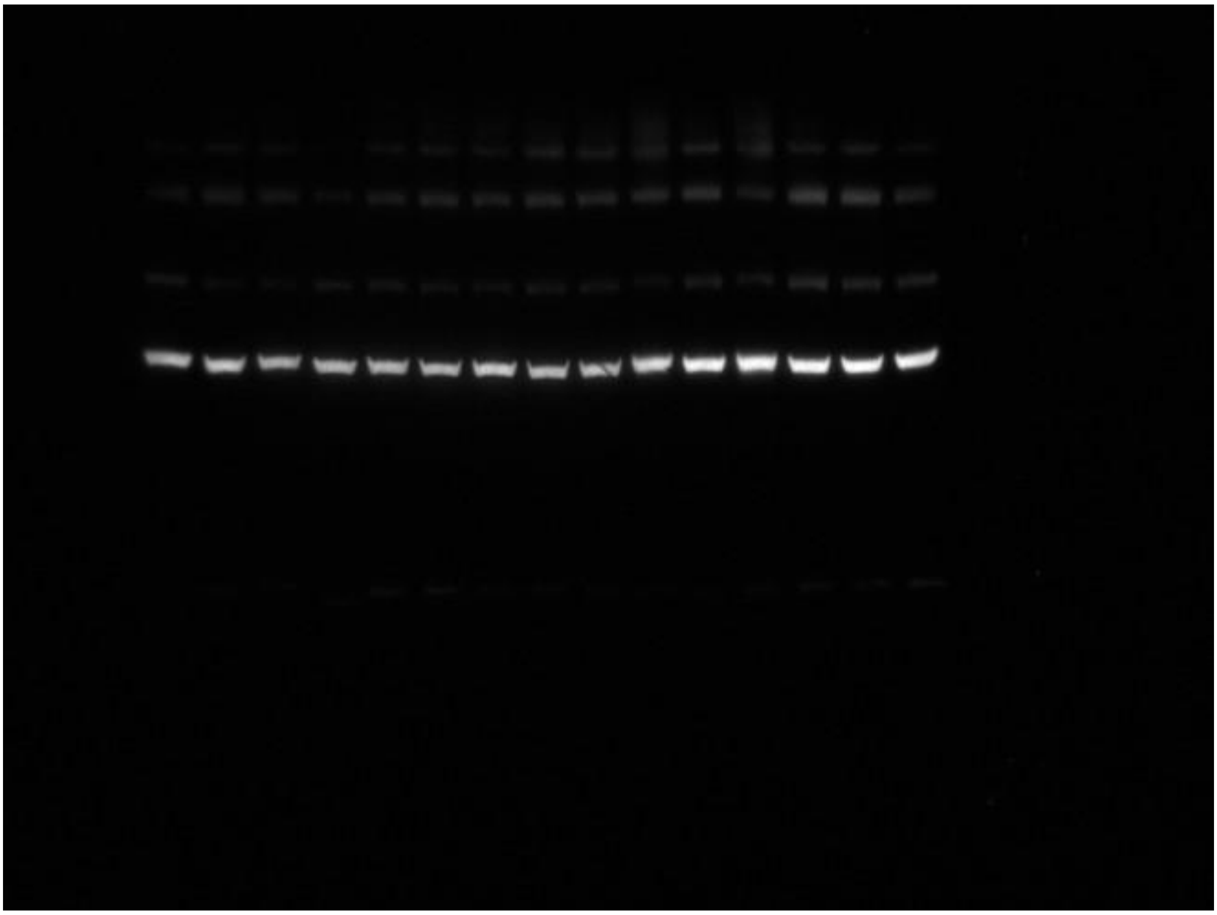

AQP4 (1) (38-43kDa) (upper) and GAPDH (below)

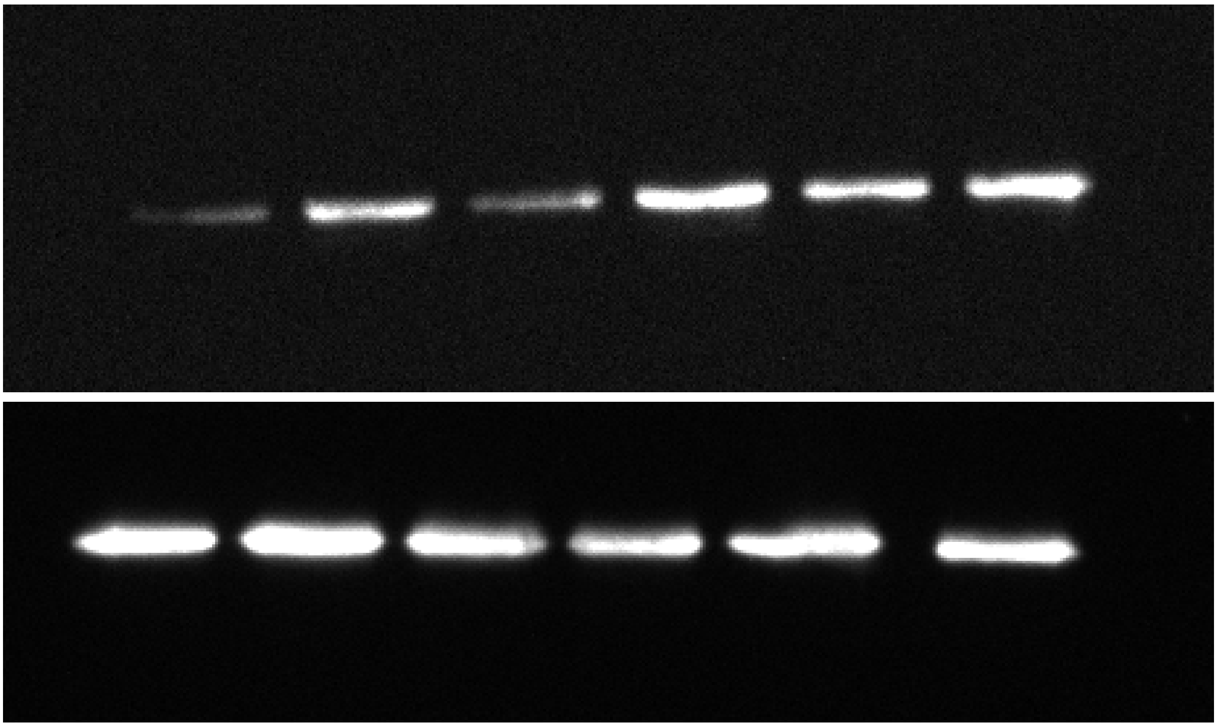

From left: 3 Control, 3 18h HE

AQP4 (2) (38-43kDa):

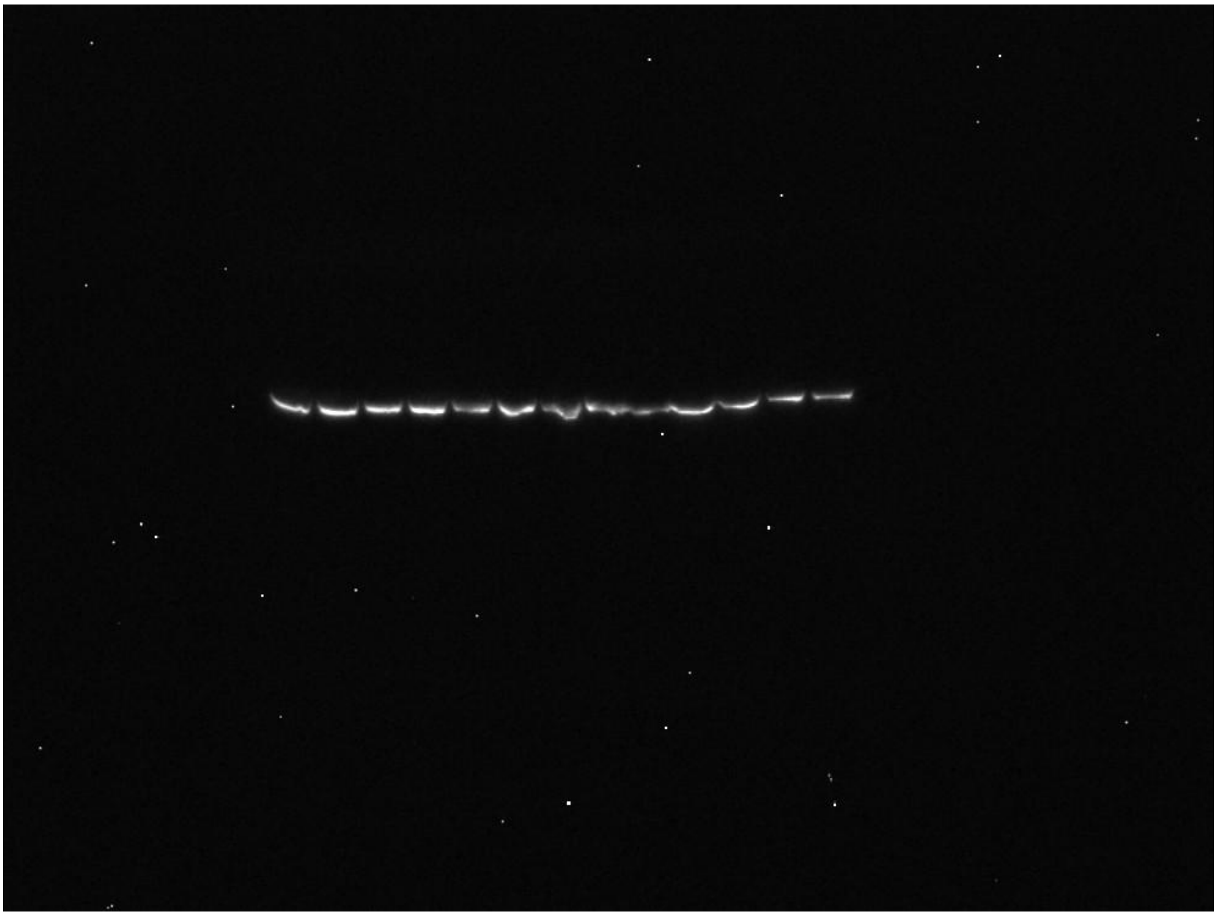

From left: 4 Control, 4 12h HE, 5 24h HE

GAPDH to AQP4 (2)

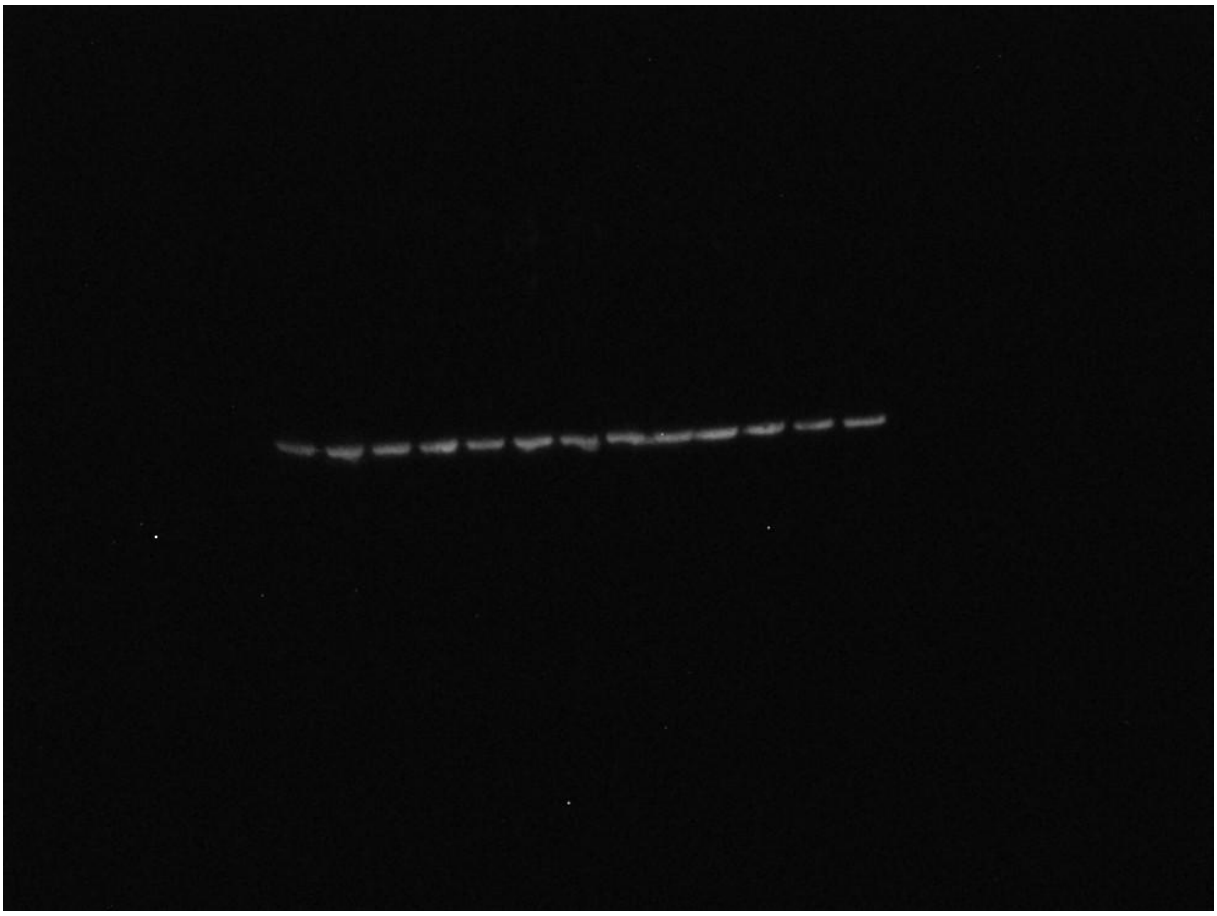

AQP4 (3) (38-43kDa):

Marker

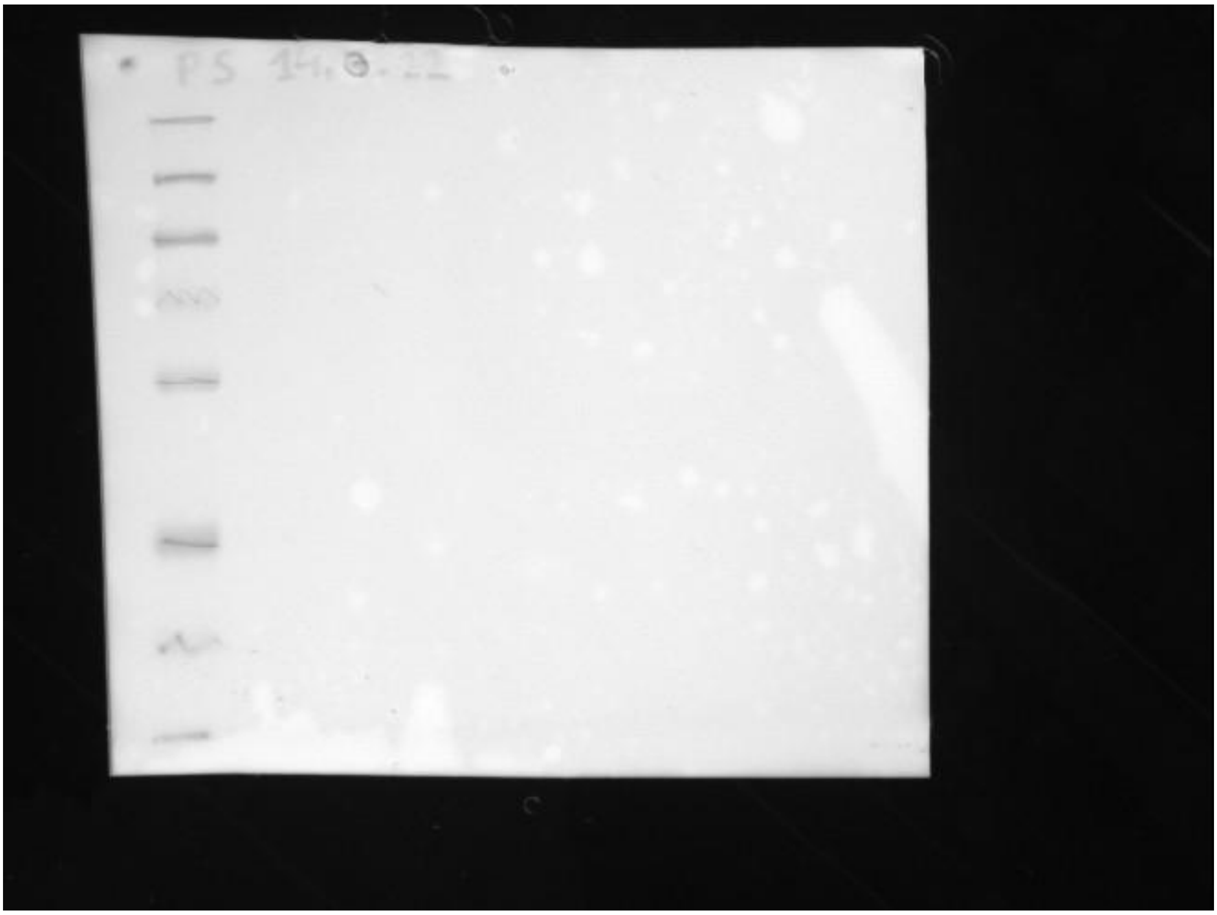

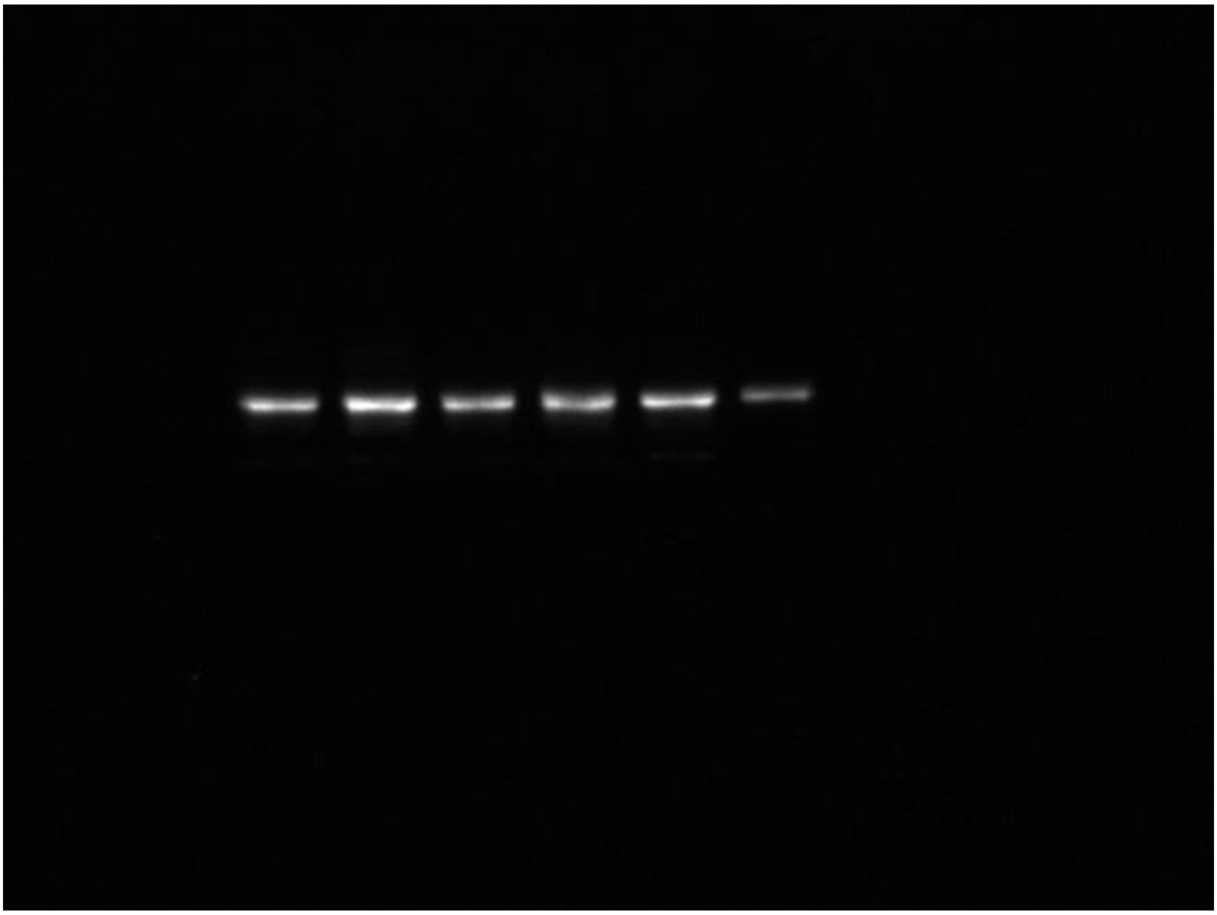

From left: 3 Control, 3 4h HE

GAPDH to AQP4 (3) marker (upper), bands (below):

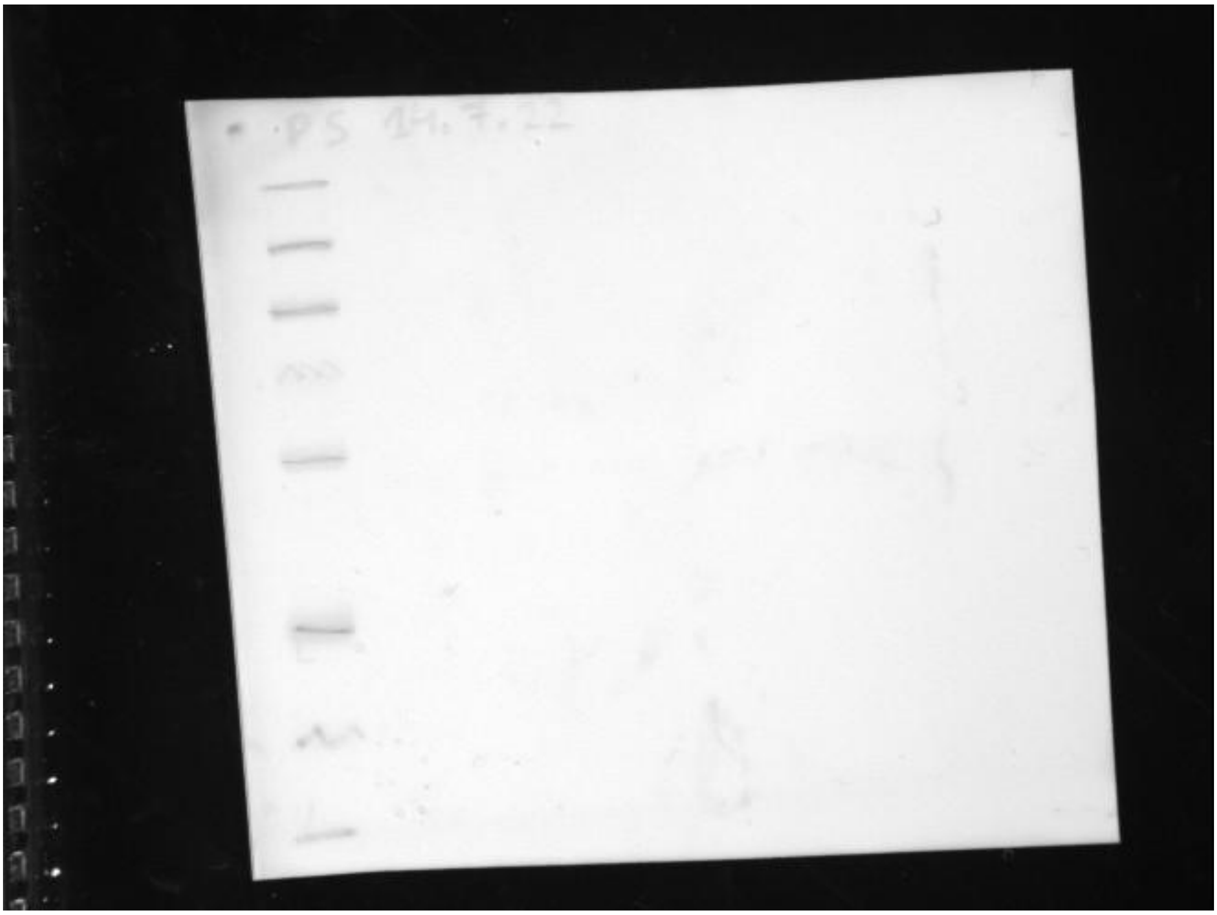
PS 11.7.22

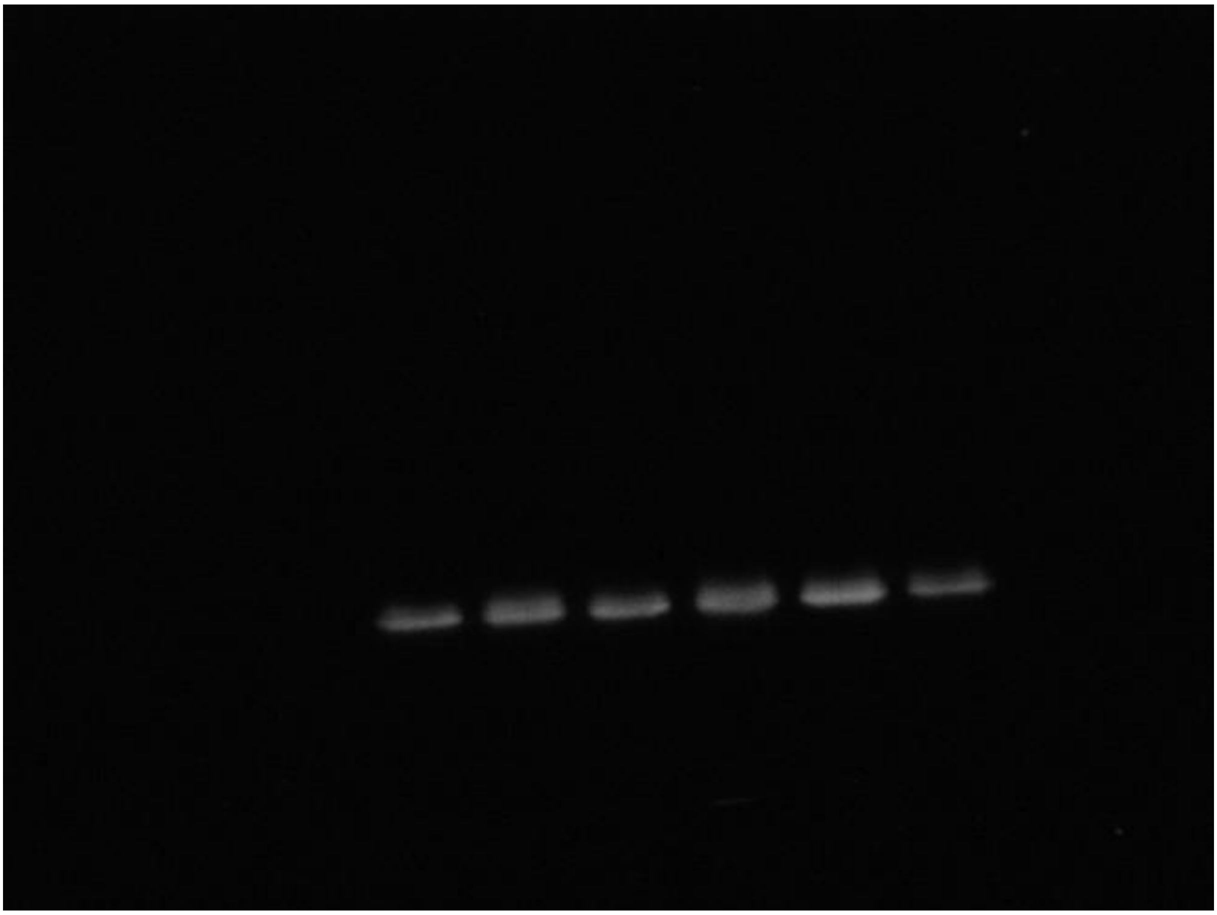

AQP4 (4) (38-43kDa):

Marker

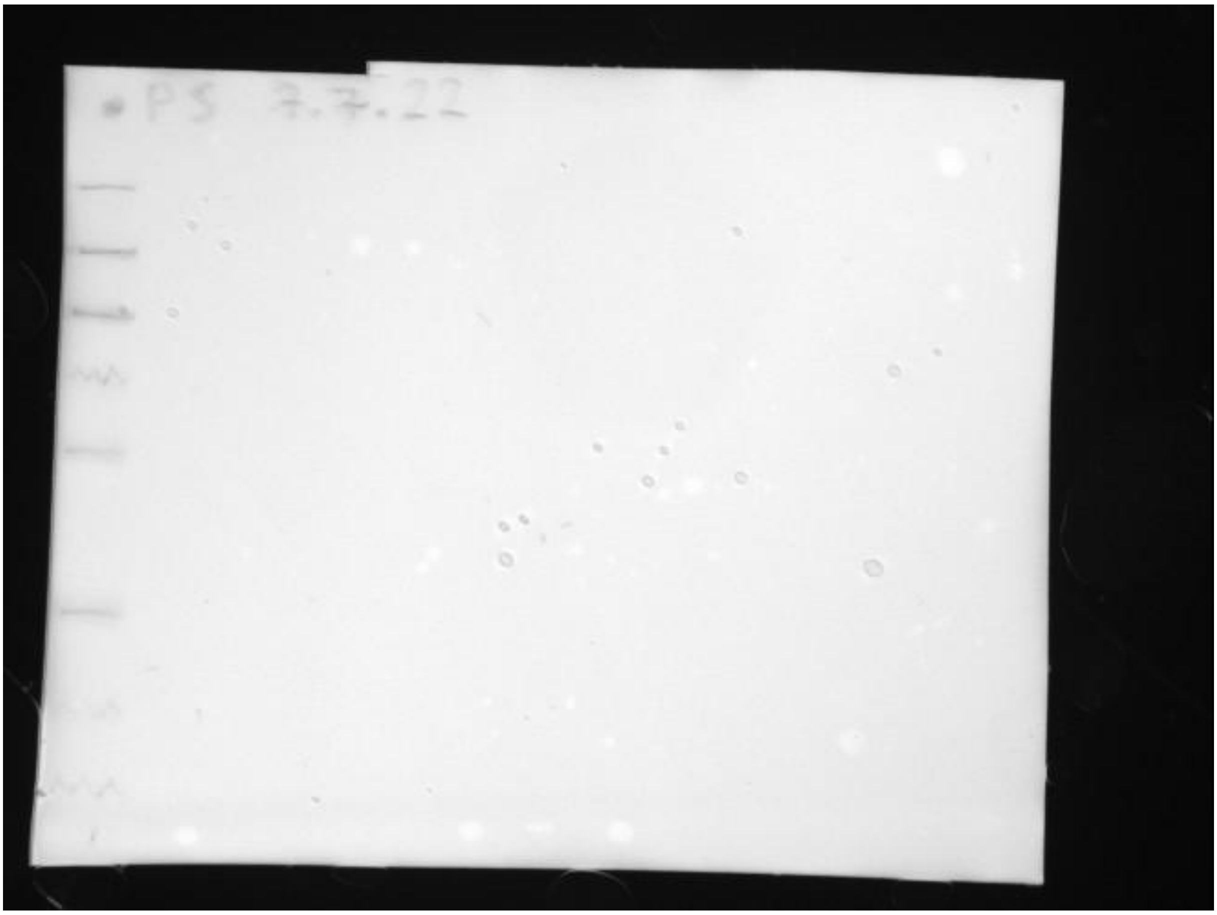

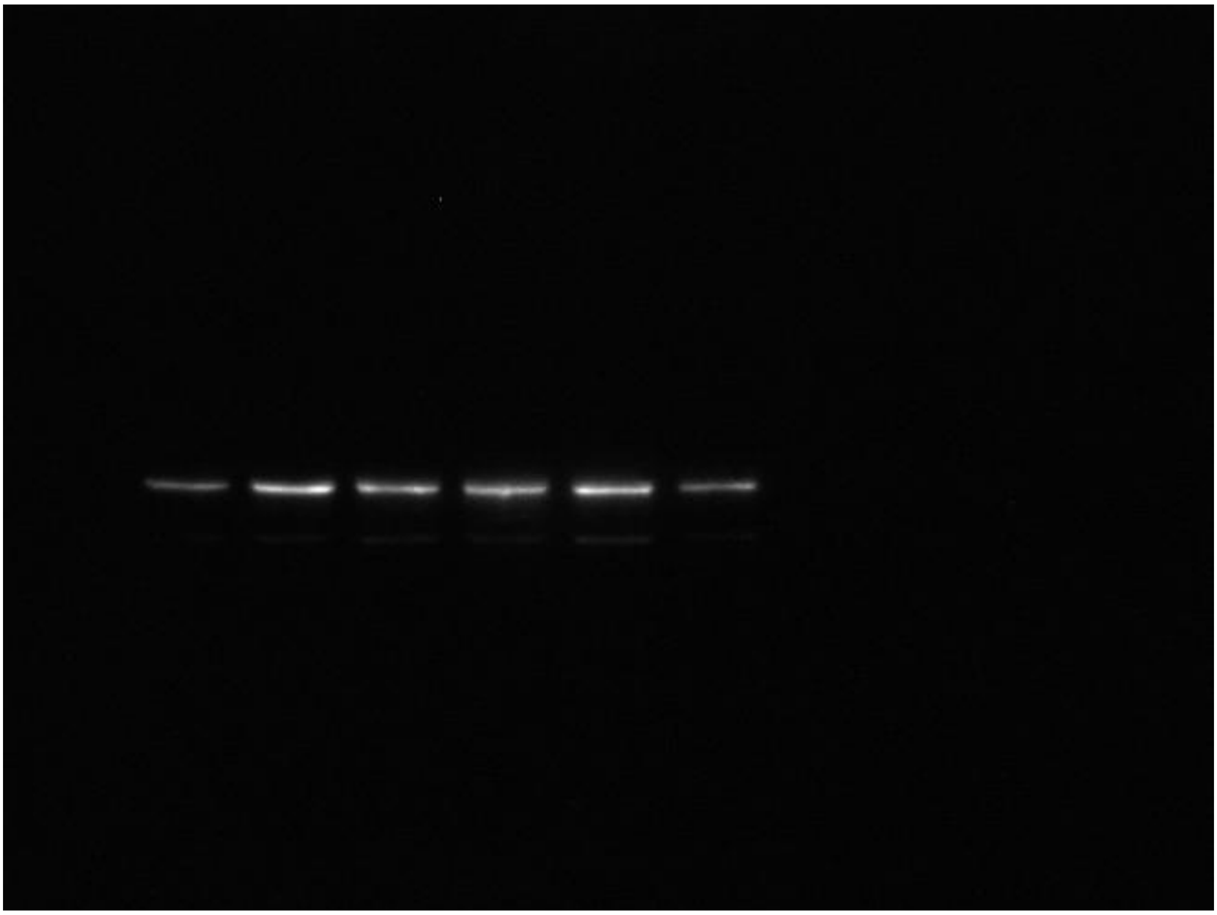

From left: 3 Control, 3 12h HE

GAPDH to AQP4 (4) marker (upper), bands (below):

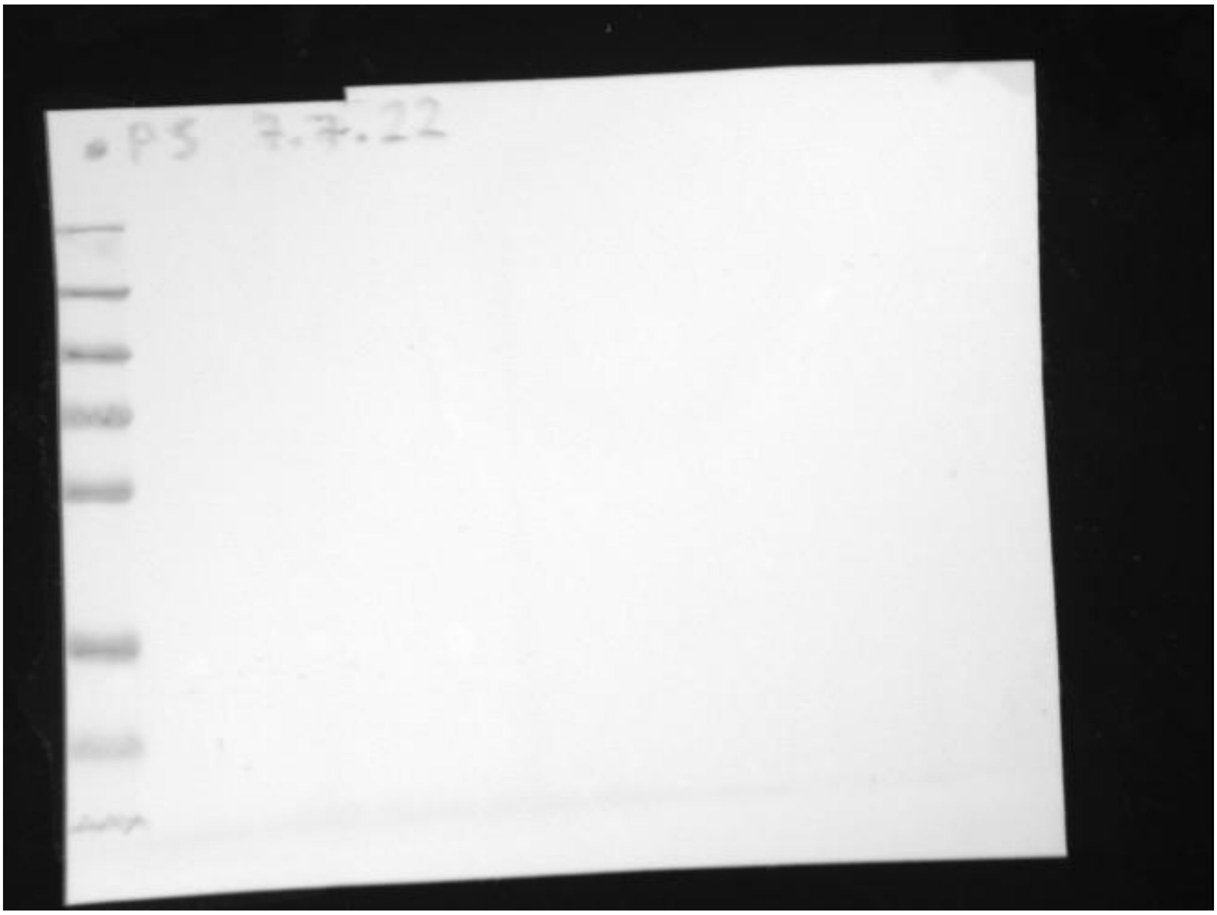
P5 7.7.22

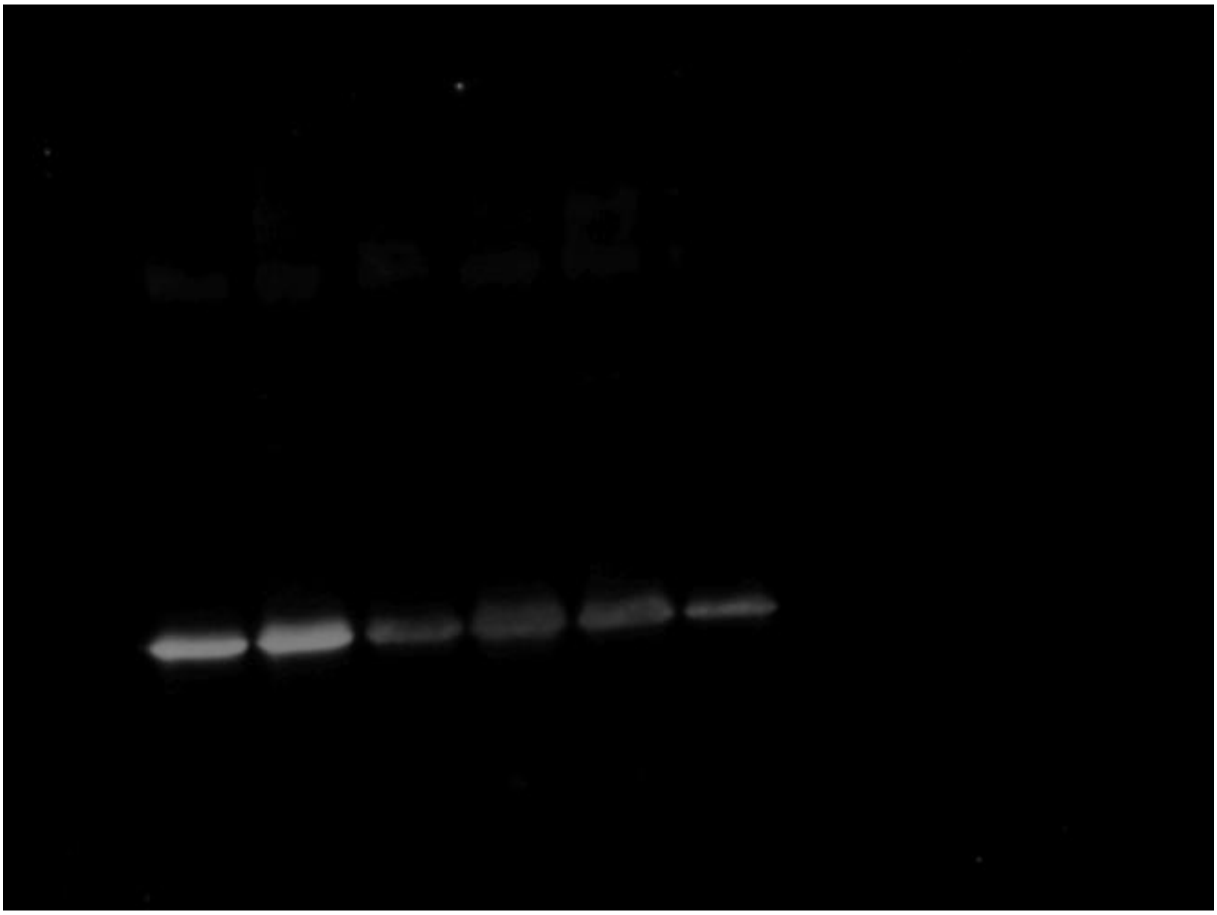

AQP4 (5) (38-43kDa):

Marker

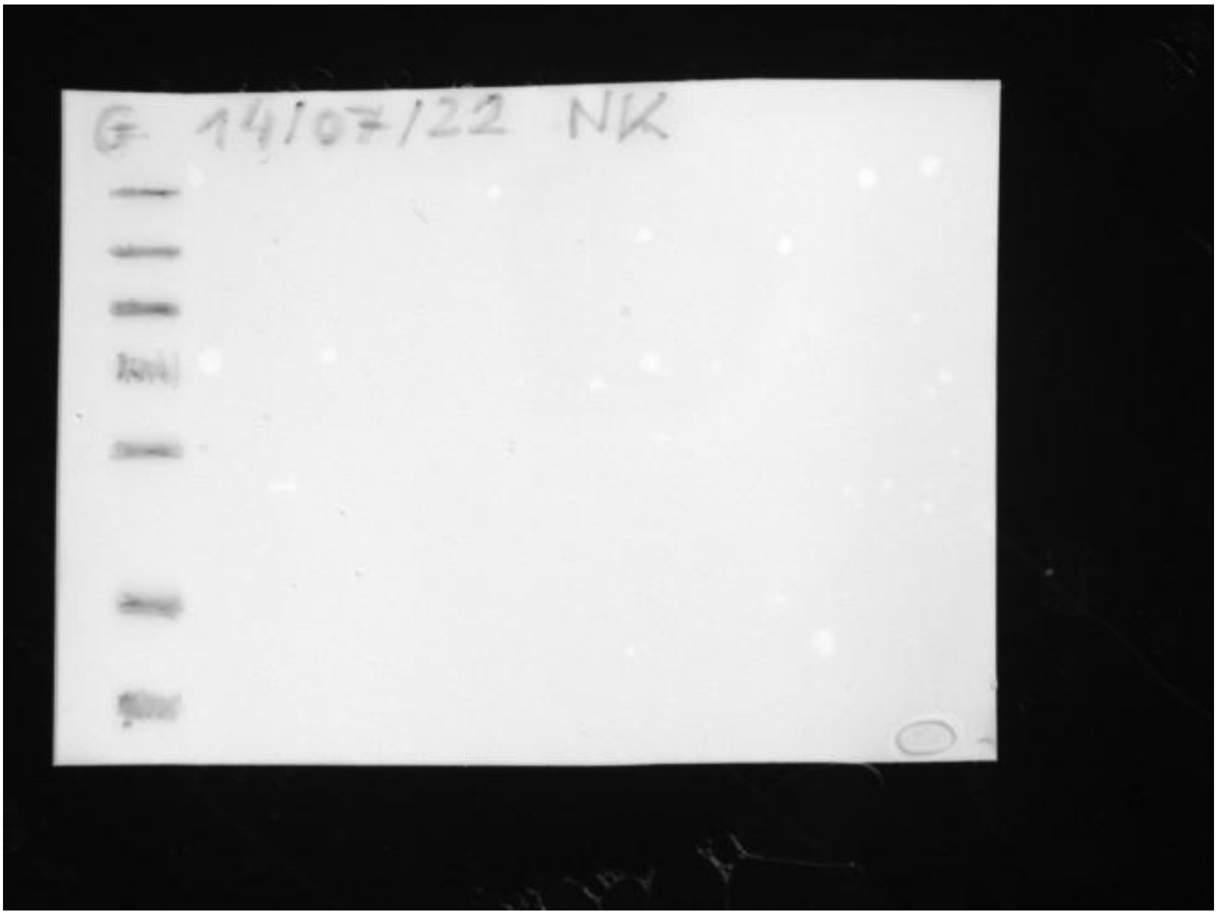

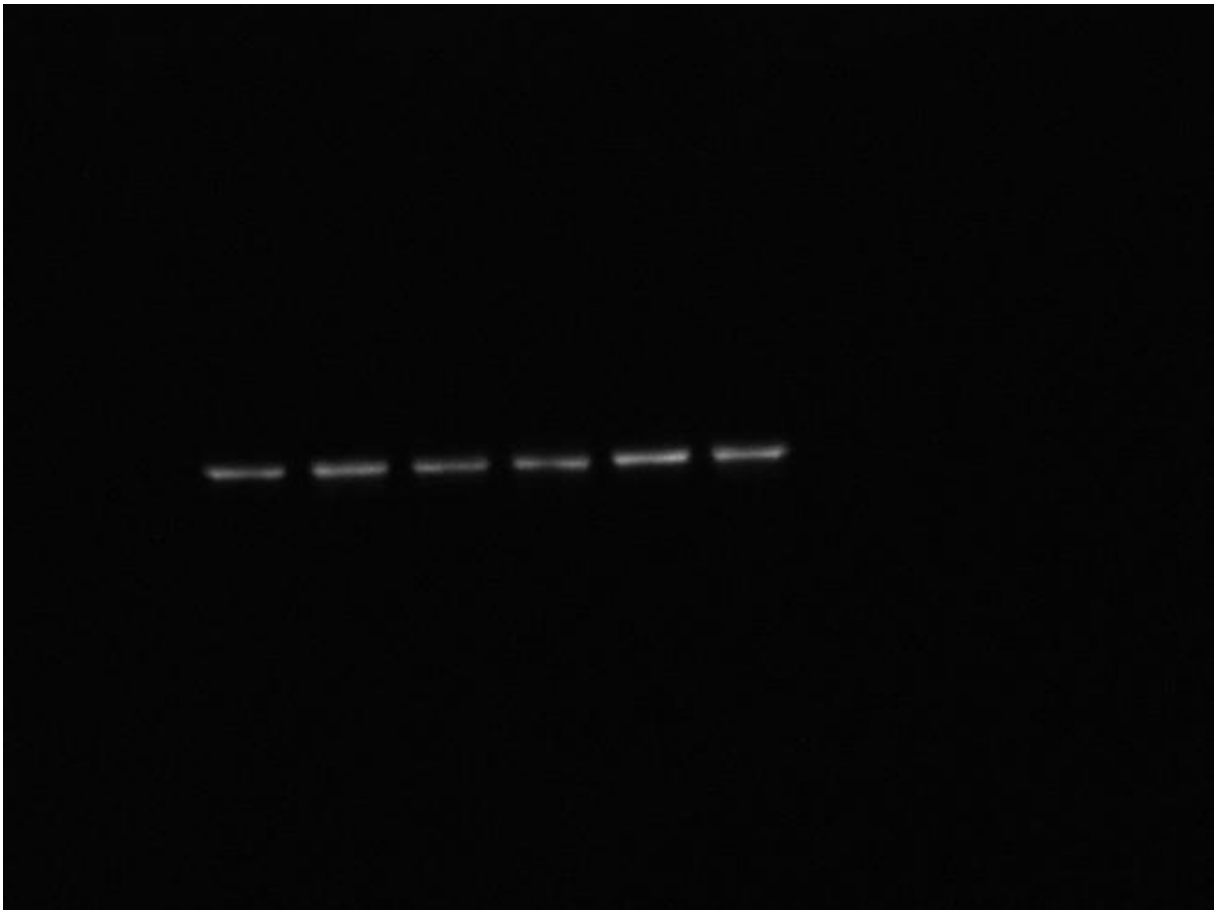

From right: 3 Control, 3 24h HE

GAPDH to AQP4 (5) bands:

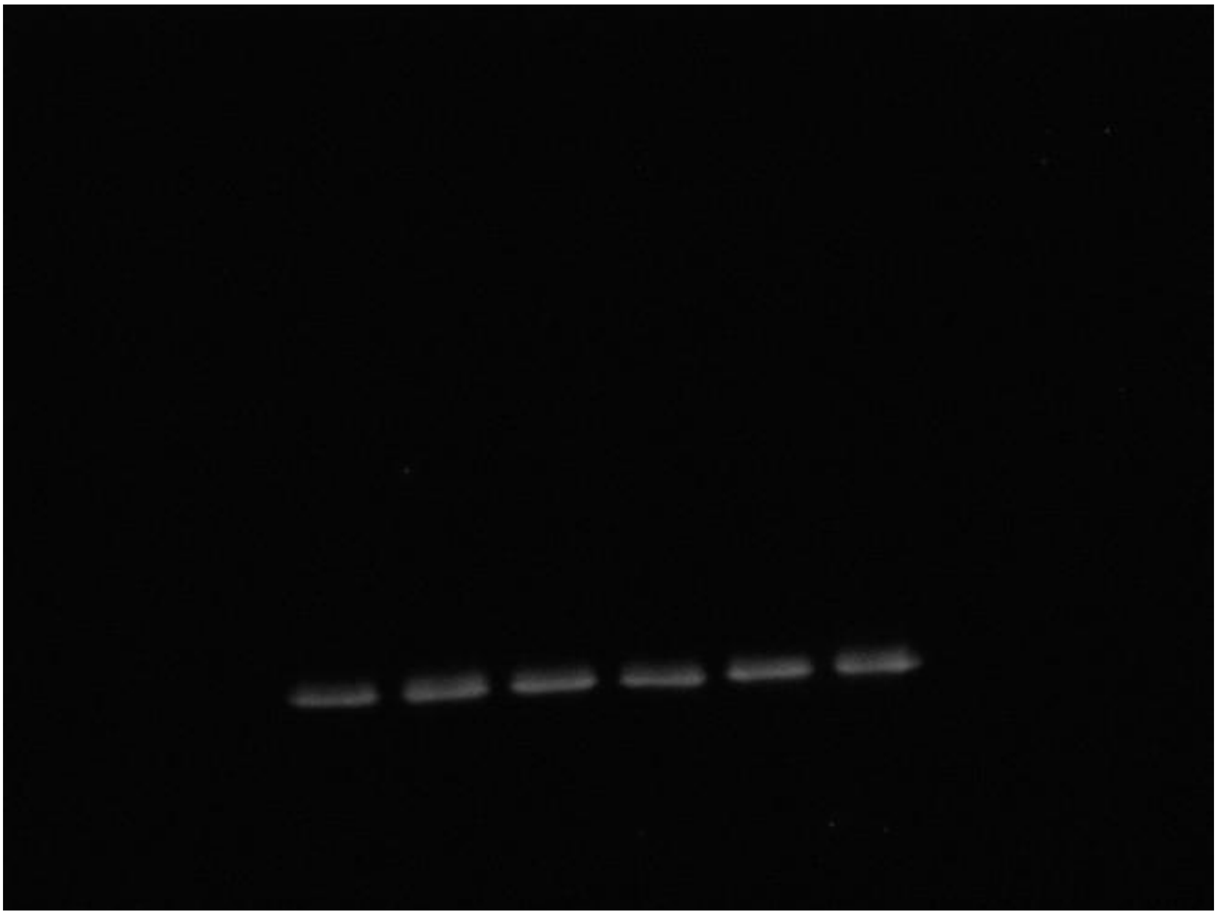

AQP4 (6) (38-43kDa):

Marker

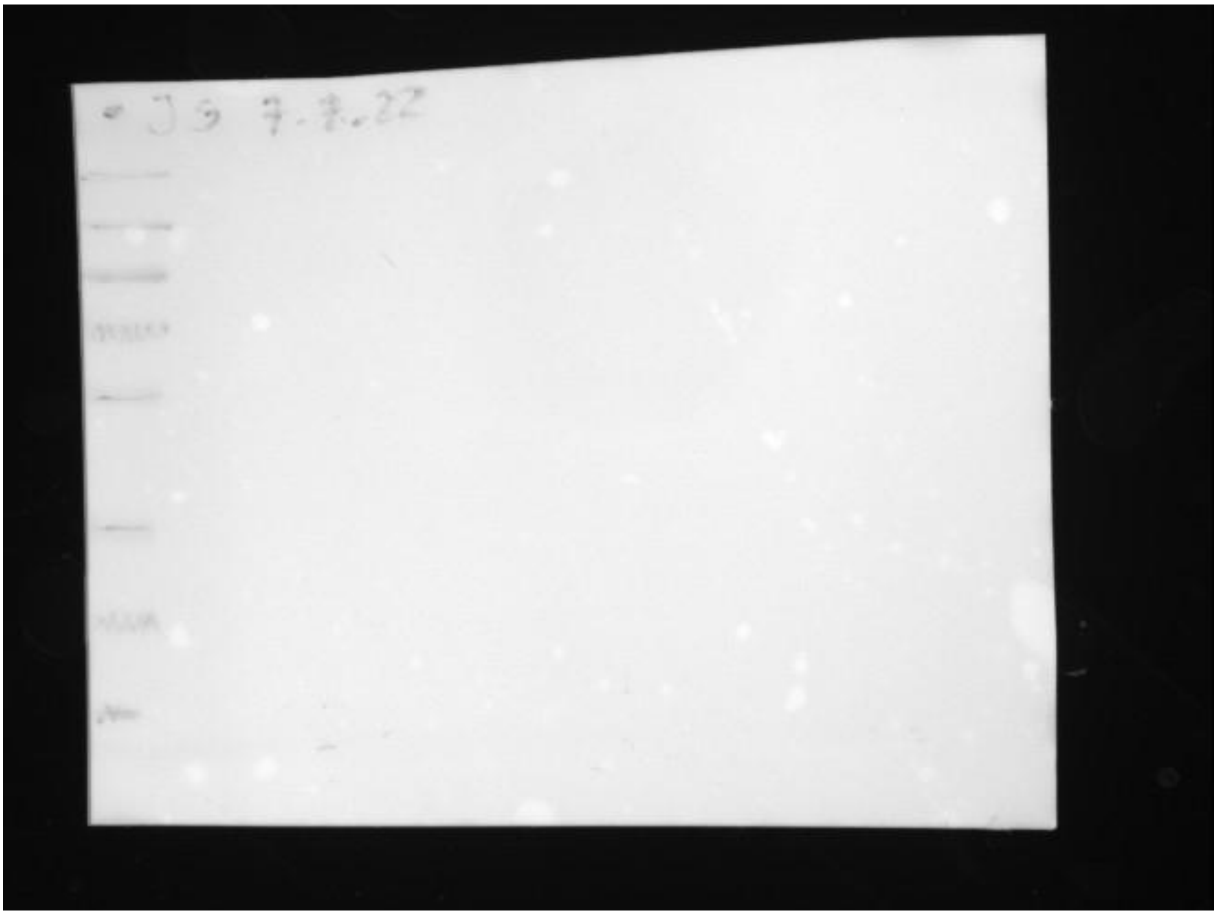

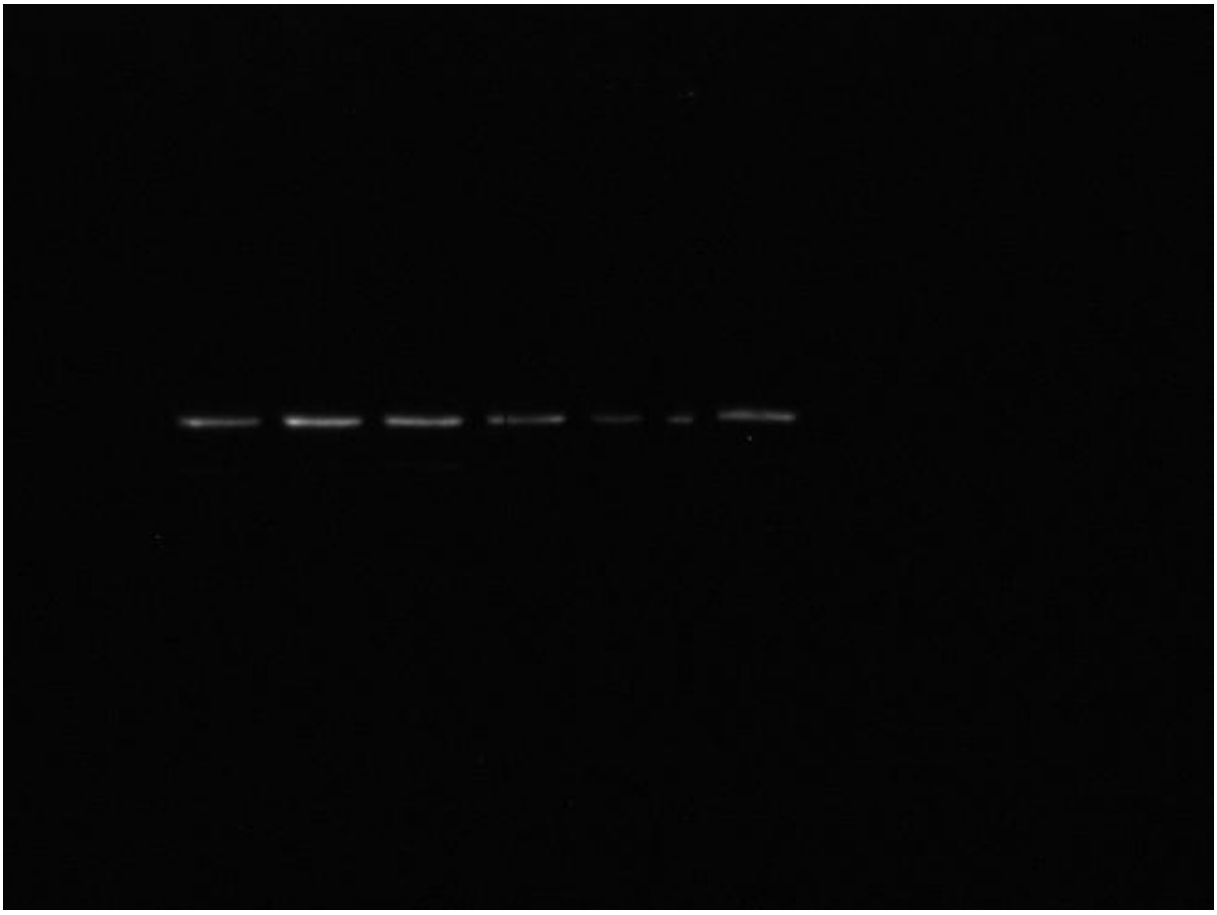

From left: 3 Control, 3 18h HE

GAPDH to AQP4 (6) marker (upper), bands (below)

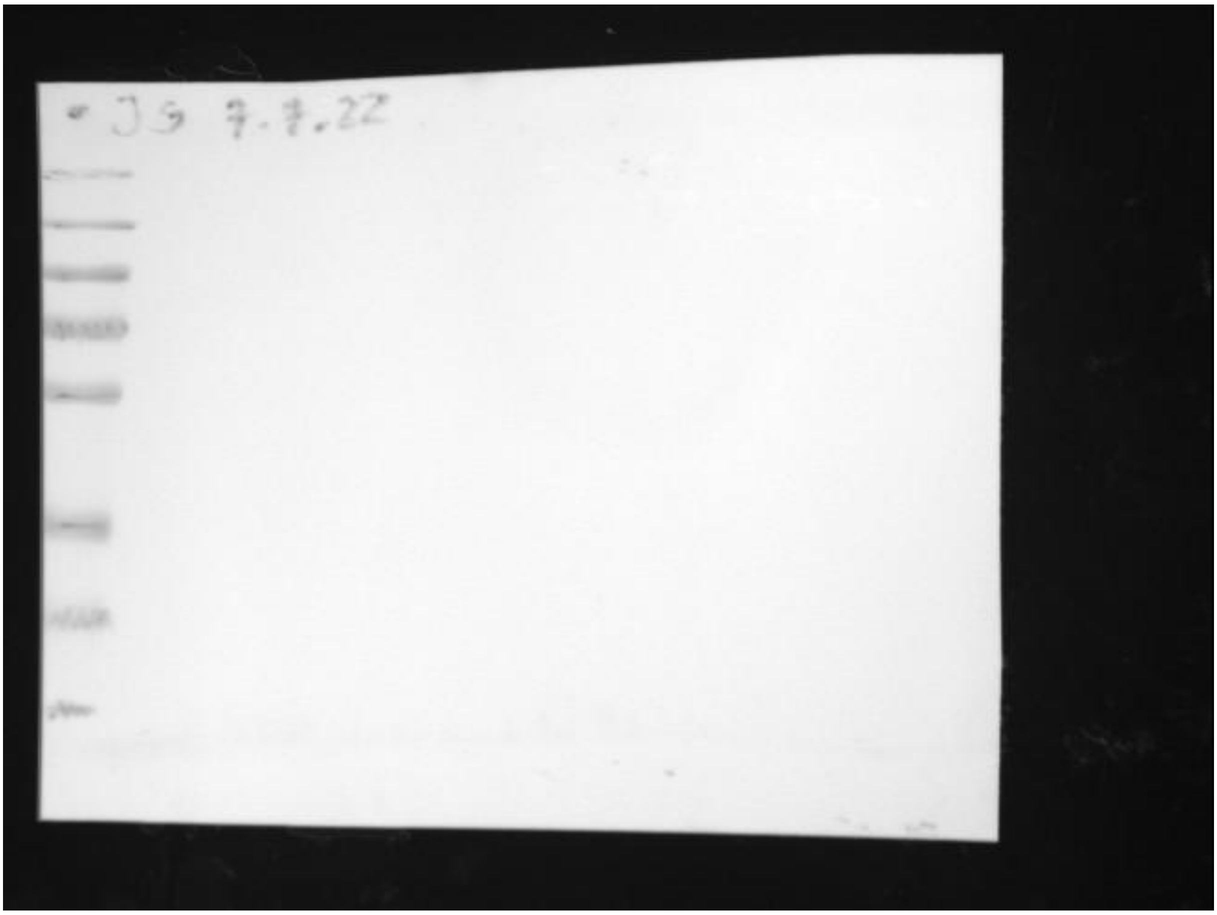
JS 7.7.22

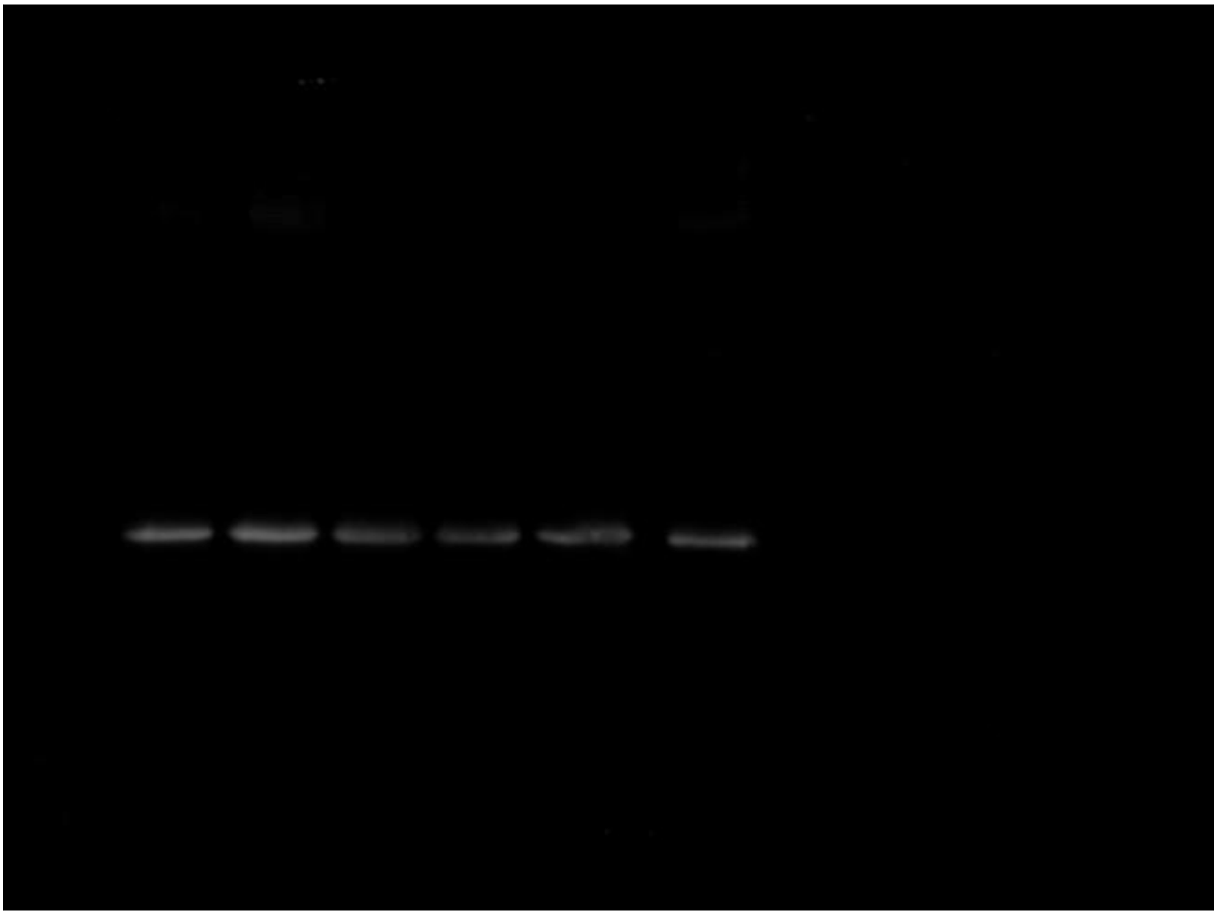

Supplement: Supplementary file 1 — Supplementary Material 1 [file 40478_2025_2045_MOESM1_ESM.pdf]
