# Supplementary material for: Oedematic-atrophic astrocytes in hepatic encephalopathy
Source: Acta Neuropathol Commun. 2025 May 31;13:122. doi: 10.1186/s40478-025-02045-5 (PMC12125882; doi:10.1186/s40478-025-02045-5)
Supplement: Supplementary file 2 — Supplementary Material 2 [file 40478_2025_2045_MOESM2_ESM.docx]

**Supporting Material and Methods**

(complementary to the information in the main text)

**Mouse housing**

Male mice used for this study were hosted under 12 h light/dark cycles with free access to food and water. Specifics of the animal diet composition: SSNIFF rat and mouse maintenance chow, composed of grain and grain by-products, oil seed products, vegetable oils, minerals (calcium, phosphorus, sodium, potassium, magnesium), vitamins (choline chloride, inositol, vitamin E (tocopherol), vitamin B12 (cyanocobalamin), vitamin B1 (thiamine), nicotinic acid, vitamin A (retinol), pantothenate, vitamin B6 (pyridoxine), vitamin K3 (menadione), vitamin B2 (riboflavin), biotin, folic acid), trace elements (iron, magnesium, zinc, copper, iodine, selenium, cobalt). The diet is coated with inert silicon dioxide to reduce the potential for clumping during autoclaving. The bedding was lignocel rettenmair. Caging system: conventional for MMRC PAS colony, IVC (ventilated racks) for the CofNT colony, with autoclaved cages before use. Enrichment used for housing the mice were polycarbonate houses, sterile cellulose tissues, wooden shavings, lignocell string-like strips.

**Azoxymethane model of ALF in mice**

The ALF in mice was induced by a single intraperitoneal (i.p.) administration of hepatotoxin azoxymethane (AOM), (Sigma-Aldrich, A5486, Poznań, Poland) at 100 mg/kg body mass. The body temperature of animals was maintained with the heating pad (37°C), whereas the hypoglycaemia and dehydration were prevented with two (8 and 16 hours post-AOM) i.p. injections of 500 μl of 5% glucose and 0.18% saline solution.

**Serum biochemical analysis**

Concentration of mice serum glucose, ammonium, alanine aminotransferase (ALT), total protein, cholesterol, and alkaline phosphatase (ALP) in Control, 4, 12, 18, and 24 hours post AOM injection were analysed using IDEXX Catalyst One (IDEXX Laboratories, Inc., Westbrook, Maine, U.S.A.). Briefly, blood was immediately collected to heparin tube and according to IDEXX protocols, Chem 15 CLIP were used. Separately, maintaining a 2-hour interval between other sample measurements, the ammonia concentration was measured immediately after plasma collection, using an Ammonia individual slide (IDEXX Laboratories, Inc., Westbrook, Maine, U.S.A.).

**Transmission electron microscopy of human brain tissue**

From 2x2x2 cm blocks of human cortex, four independent and randomly spaced sections were collected from every donors. Sections were fixed in 2% paraformaldehyde and 2.5 % glutaraldehyde in 0.1 M cacodylate buffer, pH 7.4 solution for 24 h (at 4°C) and placed in a mixture of 1% OsO4 and 0.8% K_4_[Fe(CN)_6_]. Astrocytic content in the neuropil of the frontal cortex was estimated from 56 randomly selected sections obtained from each of the four control patients and 55 sections from three HE patients. The average distance between a proximal end of the active zone (AZ) and the nearest astrocytic leaflet was quantified. For image analysis, ImageJ processing software was used. Representative 118 cortical synaptic complexes from control and 118 from HE sections were analysed.

**Transmission electron microscopy of mouse brain tissue**

Anaesthetised mice (2% isoflurane) were perfused through the ascending aorta with 2% paraformaldehyde and 2.5 % glutaraldehyde in 0.1 M cacodylate buffer, pH 7.4. The frontal cortex of control and HE mice (24 h post-AOM injection) was fixed in the mentioned solution for 24 h (at 4°C) and placed in a mixture of 1% OsO4 and 0.8% K4[Fe(CN)6], as previously described (13). Astrocytic content in the neuropil of the frontal cortex was estimated from 15 randomly selected sections obtained from each of the three control mice and 15 sections from four AOM mice. Sections with clearly visible fragments of cell bodies were excluded, but the remaining astrocytic processes were not divided morphologically, apart from the perivascular ones. Following parameters were quantified: astrocytic area content on each section, the average number of astrocytic leaflet apposition to the synaptic cleft, the average distance between a proximal end of the active zone (AZ) and the nearest astrocytic leaflet, the average distance between AZ to nearest AZ and composition of neuropil along AZ to nearest AZ paths. For image analysis, ImageJ processing software was used. Representative sizes and types of 268 cortical synaptic complexes from control and 268 from HE sections were analysed.

**Brain preparation and immunohistochemistry of human brain tissue**

Cortical tissue was cut into 90 µm sections (Leica CM1860 cryostat) and were proceeded according to standard protocol. Briefly, after 90 min of 4% PFA fixation, antigen retrieval was performed using boiling sodium citrate buffer (Bioshop Life Science Products, 10 mM concentration; Tween 20, VWR, 0.05% concentration). Next, slices were blocked for 2h with normal goat serum (10%, diluted with 1% Triton X-100), and incubated overnight with GFAP primary antibody (1:500, sc-33673, Santa Cruz Biotechnology, Texas, USA). Next, goat anti-mouse IgG Alexa Fluor 488 secondary antibody (1:500, Life Technologies) were used. For cell nuclei staining 5 μM Hoechst (1:500, Life Technologies, 33258) was used. Procedures for negative controls were carried out in the same manner except that the primary antibodies were omitted. Z-Stack photos were conducted using a confocal laser scanning microscope (Axio Imager Z2 LSM 700, Carl Zeiss Meditec, Germany).

**Brain preparation and immunohistochemistry of mouse brain tissue**

The brains of C57Bl mice, divided into control and HE (4, 12, 18, 24 h AOM post-injection) groups, were quickly removed, immediately frozen in powdered dry ice, and stored at -80ºC. 20 µm thick frontal cortex sections (Leica CM1860 cryostat) were proceeded according to standard protocol. Briefly, after 20 min of 4% PFA fixation, 1 h blocking with normal goat serum (10%, diluted with 0.1% Triton X-100), overnight incubation with Phospho-Ezrin antibody (Cell Signaling Technology, 3726T, Massachusetts, USA) 1 h incubation with goat anti-rabbit IgG Alexa Fluor 546 secondary antibody (1:500, Life Technologies, Carlsbad, California, USA) were performed. For astrocytes labelling, GFAP primary antibody (1:500, sc-33673, Santa Cruz Biotechnology, Texas, USA) and goat anti-mouse IgG Alexa Fluor 488 secondary antibody (1:500, Life Technologies) were used. For cell nuclei staining 5 μM Hoechst (1:500, Life Technologies, 33258) was used. Procedures for negative controls were carried out in the same manner except that the primary antibodies were omitted. Z-Stack photos were conducted using a confocal laser scanning microscope (LSM 780, Carl Zeiss Meditec, Germany).

For fluorescence intensity analysis of astrocyte processes and quantification of astrocytes single branches and neuronal volume, astrocyte TdT+ mice line was used. Control TdT+ and 24h post AOM injection HE mice were transcardially perfused with 0.1 M PBS, followed by 4% PFA (Sigma Aldrich, 158127) in PBS. Brains were dissected, incubated overnight in 4% PFA, and saturated with 30% sucrose (Merck Life Science, cat. no. 1076515000) in PBS at 4 °C for the next 24 hours. Next, the brains were transferred into O.C.T. (Sakura Finetek Europe B.V., 4583) and frozen in -30°C isopentane (VWR, 24872.298). Frontal cortex 30 μm sections (Leica CM1860 cryostat) were prepared as free-floating sections in anti-freeze solution (30% sucrose (Merck Life Science, 84100); 30% glycerol (VWR, 443320113); 0.1 M PBS, pH 7.4). After PBST washing, antigen retrieval was conducted using 10  mM sodium citrate buffer (Bioshop Life Science Products, CIT001.1) with 0,05% Tween 20 (Sigma Aldrich, P7949). Next, sections were blocked 1h in 10% donkey serum in PBST and incubated overnight with primary antibodies for anti-GFAP (1:250, Merck Life Science, Hpa056030) and anti-MAP2 (1:200, Sigma-Aldrich, M3696). Further, the slices were washed and incubated with goat anti-rabbit IgG Alexa Fluor 488 secondary antibody (1:500, Life Technologies, Carlsbad, California, USA). Finally, slices were mounted on glass slides with Fluoromount G. Z-Stack photos were conducted using an Axio Imager Z2 LSM 700 Zeiss confocal microscope.

The immunofluorescence analysis of Z-Stack images were processed using the ZEN v. 2008 (Carl Zeiss Meditec, Germany). The fluorescence intensity of Phos-ezrin was analysed from the mean grey value of Maximum Intensity Z-projected images using Image J software and normalised to analysed the astrocytes area.

**Hematoxylin and eozin liver staining**

At the selected time points post AOM injections (4, 12, 18, 24h) mice livers were exposed and examined with light microscopy. Subsequently, the liver was subjected to hematoxylin and eozin (H&E) staining. Briefly, tissue was frozen in dry-iced 99.5% acetone (Sigma-Aldrich, Poznań, Poland) at -80°C. Livers were cut into 10 µm thick sections (Leica CM1860 cryostat), and washed with an ethanol gradient (99%-70%) and water. Sections were stained with hematoxylin (Sigma Aldrich, St. Louis, MO, USA) solution and rinsed under running water for 10 min, followed with 4 min incubation with eosin (Sigma Aldrich, St. Louis, MO, USA) solution, and washed with an inverted ethanol gradient. The sections were mounted in DPX medium (Sigma-Aldrich, 06522, Poznań, Poland) and analysed using an Olympus IX71 Inverted Fluorescence Motorised Microscope (Olympus Corporation, Tokyo, Japan).

**Real-time PCR**

In the homogenates of mouse cerebral cortex, at indicated time points after AOM administration, the mRNA level of AQP4 and its isoforms, M23 and M21, was assessed with real-time PCR in Applied Biosystems 7500 Fast Real-Time PCR System (Applied Biosystems, Foster City, CA, USA). Total RNA was extracted according to the phenol-chloroform method (RNA Extracol, Eurx, Gdańsk, Poland). RNA samples (1 μg) were reversely transcribed (High-Capacity cDNA Reverse Transcription Kit; Thermo Fisher Scientific, Waltham, MA, USA) and real-time PCR analysis was performed using 1μl of cDNA in a reaction of 10 μl. Fast SG/ROX qPCR Master Mix and SYBR Green Fast SG qPCR Master Mix (Eurx, Gdańsk, Poland) were used for the analysis of total AQP4 transcript, and M23 and M1 isoforms, respectively. TaqMan assay IDs were Mm00802131_m1 for Aqp4 and Mm00607939_s1 for β-actin (Thermo Fisher Scientific, Waltham, MA, USA), whereas primer sequences for M1 and M23 isoforms were synthesised according to (1). Data are averaged triplicates of mRNA transcript levels, normalised to β-actin expression according to the 2-ΔΔCt method (2).

**Western blot**

Cerebral cortex tissue samples were collected in RIPA buffer (Thermo Fisher Scientific, Waltham, MA, USA), supplemented with 0.05% CHAPS detergent and protease/phosphatase inhibitors cocktail (1:100) (Merck, Darmstadt, Germany), homogenised, sonicated and centrifuged (10 min, 10 000 g, 4 ^o^C). Protein content in the supernatant was quantified using Pierce™ BCA Protein Assay Kit (Thermo Fisher Scientific, Waltham, MA, USA; Cat No.: 23225). Thirty micrograms of protein were run on a 10 % SDS-polyacrylamide gel (Thermo Fisher Scientific, Waltham, MA, USA) and then transferred onto a PVDF membrane with semi-dry Trans-Blot Turbo System (Bio-Rad Laboratories, CA, USA). Blots were blocked in 5% bovine serum albumin (BSA) (BioShop; Lab Empire S.C.) and incubated in 2.5 % BSA, overnight, 4 ^o^C, with primary antibody: Aqp4 (1:1000; PA5-85767, Thermo Fisher Scientific, Waltham, MA, USA), PFN1 (1:1000; P7624, Merck, Darmstadt, Germany), ezrin (1:1000; SAB4200806, Merck, Darmstadt, Germany). Protein bands were detected with host-specific secondary antibodies and developed with a chemiluminescent substrate (GE Healthcare Amersham, Piscataway, NJ, USA). After stripping, the blots were incubated with HRP-conjugated anti-GAPDH antibody (1:8000; HRP-60004, Proteintech, Manchester, UK) and developed. The chemiluminescent signal acquisition and densitometric analysis were conducted using the G-Box system (SynGene) and GeneTools software (SynGene), respectively.

**EEG** **of the freely moving mice**

Mice were anaesthetised with ketamine (75 mg/kg, Vetoquinol) and dexmedetomidine (0.5 mg/kg, Orion Pharma) for stereotactic implantation of electrodes. The 2EEG/1EMG headmounts (EEG/EMG/Plus Mouse Headmount, Pinnacle Technology, Lawrence, KS) were affixed to the exposed skull with stainless steel screws acting as EEG electrodes. The screws were positioned to be resting on the meninges. Two parietal screws were used for EEG recording and one frontal screw for reference and grounding. EMG electrodes were implanted below the neck muscles. The headmount was secured with dental acrylic cement and the skin incision was sutured below and above the headmount. Following the surgery, mice were woken up from anaesthesia with atipamezole hydrochloride (1 mg/kg, Orion Pharma) and treated with ketoprofen (5 mg/kg, Sandoz) once a day for up to 3 days*.*

The EEG recordings were conducted, after 5 days of recovery and acclimatisation, in a transparent, round cage with free access to food and water, to which mice were acclimated for one day. For EEG recording, the mice were connected to the data acquisition system (8401 Data Conditioning & Acquisition System, Pinnacle Technology), with the attachment plug tethered to the swivel at the balanced arm that, together with the revolving of the cage, allowed animals for moving freely during the EEG recording. The 3 – 4 hours recording was acquired, using Sirenia software (Pinnacle Technology), before AOM administration. The recording was carried on within 24 hours of ALF development.

For each EEG data set signal power was calculated in particular frequencies using Butterworth filters (Kadam et al., 2017): total EEG band (0–100 Hz), delta (1–4 Hz), theta (4–8 Hz), alpha (8–13 Hz), beta (13–30 Hz), gamma (30–100 Hz), high-frequency oscillations (HFO) (>80 Hz). Thirty-minute epochs, baseline, and overlapping the 4, 12, 18, and 24 h time points after AOM administration were analysed by Matlab program. The power values were normalised and presented as relative to the baseline.

**Astrocytic processes distribution analysis**

The ring shaped ROIs with 5 µm decrement were applied to Maximum Intensity Z-projected images of astrocytes. The fluorescence intensity of one astrocyte was analysed in each of the 6 ROIs and normalised to the fluorescence intensity of the individual, not-saturated soma. The ROI fluorescence intensity in distance between 15-20µm from the soma was analysed as characteristic of fine processes and considered as a volume fraction (VF) parameter. Analysis was based on the mean grey value of Z-Stack images using Image J software.

**Sholl analysis of astrocytes**

Astrocytes Z-stack images were traced and skeletonised using SNT plug to Fiji. Sholl analysis was performed from soma centre every 2µm and number of intersections was counted. Based on skeletonised model, number of junctions (treated as points from which at least 2 branches originate) and length of the longest branch were calculated.

**Astrocytes and neurons volume quantification**

The volume of astrocyte single branch and neurones in the same ROI was measured using the MeasurementPro, Imaris 8.4.2 software (Bitplane AG). Briefly, three-dimensional surface rendering of individual astrocytes was generated, and within the area occupied by a single astrocytic branch, MAP2-staining-based neuronal surfaces were constructed. Renders were created based on the pixel gradient intensity algorithm, using 488 nm and 546 nm channels. Renders were threshold to ensure all processes of astrocytes were properly reconstructed. Pictures were blinded before analysis.

**Microdialysis of the freely moving mice**

A microdialysis cannula was implanted into the frontal cortex (coordinates: AP +2.0, ML -0.8, DV -1.5) under isoflurane (Aerrane, Baxter) anaesthesia, and mouse recovery time was set as two days. After this time, CMA7 microdialysis probe (2mm, Harvard Apparatus, Massachusetts, United States) with aCSF (composition in mM: NaCl (130), KCl (5), CaCl_2_ (2.5), MgSO_4_ (1.3), KH_2_PO_4_ (1.25), NaHCO_3_ (26), and D-glucose (10)) flowing through at a rate of 2.5ul/min, was implanted to control or AOM-induced mice. Microdialysates from freely moving mice brains were collected every 40 minutes (100 µL) and immediately frozen at −80°C.

**High-performance liquid chromatography**

Glutamate extracellular concentration was measured in brain microdialysates using HPLC with fluorescence detection after derivatisation in a timed reaction with o-phthalaldehyde with mercaptoethanol. Samples (50μL) were then injected into a 5μm Bio-Sil C18 Hl column (250 × 4.6 mm, BIO-RAD), with a mobile phase of 0.075 M KH2PO4 solution containing 10% v/v methanol, pH 6.2 (solvent A), and methanol (solvent B). Concentrations were averaged from the 2 fractions closest to the specified time point.

**Statistical analysis**

All experiments were carried out with replicates depending on the experiment type. When two population groups of responses were examined, depending on the results obtained, the Student’s t-test or the Mann-Whitney U test was applied (see legends to figures). Statistical significance was determined by one-way analysis of variance (one-way ANOVA) followed by Dunnett's post hoc comparisons. Error bars represent the SD, which is specifically indicated, *P < .05, **P < .01, and ***P < .001. If not indicated with asterisk, the analysis did not return values indicating significance. All statistical analyses were performed using GraphPad Prism 7 (GraphPad Software, Inc., USA).
