# Supplementary material for: Oedematic-atrophic astrocytes in hepatic encephalopathy
Source: Acta Neuropathol Commun. 2025 May 31;13:122. doi: 10.1186/s40478-025-02045-5 (PMC12125882; doi:10.1186/s40478-025-02045-5)
Supplement: Supplementary file 3 — Supplementary Material 3 [file 40478_2025_2045_MOESM3_ESM.docx]

Table 1. Information of analyzed human brain tissue.

| **Group** | **Patient number** | **Sex** | **Age** | **Cause of death** | **Type of analysis** |
| --- | --- | --- | --- | --- | --- |
| **Control** | K302 | M | 50 | road accident | Sholl/ultrastructural |
|  | K889 | M | 63 | road accident | Sholl/ultrastructural |
|  | K1296 | M | 25 | road accident | Sholl |
|  | K21/MS | M | 58 | unknown | ultrastructural |
|  | K25/MS | M | 61 | unknown | ultrastructural |
| **HE** | 5587 | F | 48 | IV stage HE | Sholl/ultrastructural |
|  | 5590 | M | 46 | IV stage HE | Sholl/ultrastructural |
|  | 5591 | M | 52 | IV stage HE | Sholl/ultrastructural |
